# Learning radical excited states from sparse data

**DOI:** 10.1039/d5sc04276c

**Published:** 2025-08-12

**Authors:** Jingkun Shen, Lucy E. Walker, Kevin Ma, James D. Green, Hugo Bronstein, Keith T. Butler, Timothy J. H. Hele

**Affiliations:** a Department of Chemistry, University College London Christopher Ingold Building WC1H 0AJ UK t.hele@ucl.ac.uk; b Yusuf Hamied Department of Chemistry, University of Cambridge Cambridge CB2 1EW UK; c Cavendish Laboratory, University of Cambridge Cambridge CB3 0HE UK

## Abstract

Emissive organic radicals are currently of great interest for their potential use in the next generation of highly efficient organic light emitting diode (OLED) devices and as molecular qubits. However, simulating their optoelectronic properties is challenging, largely due to spin-contamination and the multiconfigurational character of their excited states. Here we present a data-driven approach where, for the first time, the excited electronic states of organic radicals are learned directly from experimental excited state data, using a much smaller amount of data than typically required by Machine Learning. We adopt ExROPPP, a fast and spin-pure semiempirical method for the calculation of the excited states of radicals, as a surrogate physical model for which we learn the optimal set of parameters. To achieve this we compile the largest known database of organic radical geometries and their UV-vis data, which we use to train our model. Our trained model gives root mean square and mean absolute errors for excited state energies of 0.24 and 0.16 eV respectively, improving hugely over ExROPPP with literature parameters. Four new organic radicals are synthesised and we test the model on their spectra, finding even lower errors and similar correlation as for the training set. This paves the way for the high throughput discovery of next generation radical-based optoelectronics.

## Abstract 2

The first example of machine learning directly from the excited states of radicals, by training the surrogate model ExROPPP on the structures and experimental spectra of 81 organic radicals.

## Graphical abstract

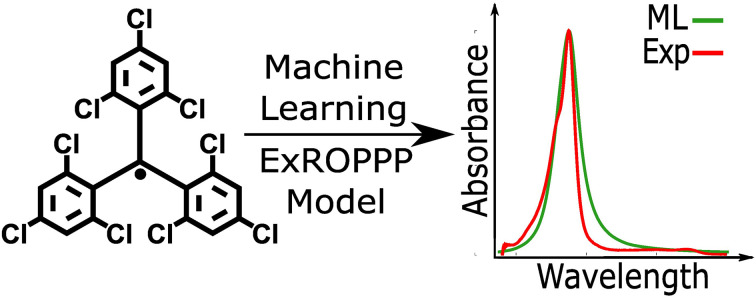

## 1 Introduction

Recent years have shown a great interest in radicals for organic light emitting diodes (OLEDs), which display internal quantum efficiencies (IQE) of near 100% and intense emission in the deep red, near-infrared (NIR) and infrared (IR) spectral regions, features which are unusual and highly desirable.^1–8^ These radical OLEDs, based on organic monoradicals, offer an alternative for the next generation of highly efficient lighting. Furthermore, optical readout of the quartet state of some radicals has potential applications in quantum information science and paves the way for next-generation molecular qubits.^9^ However, historically many organic radicals have been nonemissive,^10^ such that trial-and-error exploration of chemical space is inefficient, and there is therefore a large and unmet need for a method that facilitates the fast, accurate, and spin-pure calculation of the low-lying excited states of a wide variety of radical molecules. Such a computational method would also be invaluable for the high-throughput screening of radicals for their UV-visible spectra. This work focuses on organic monoradicals, *i.e.* molecules with only one unpaired electron, however, it should be noted that the excited state properties of organic biradicals and organic radicals with many unpaired electrons are generally different to those discussed here.^11,12^

Calculating the excited electronic states of radicals is challenging due to spin-contamination and their multiconfigurational character. There exist several highly accurate methods for calculation of the excited states of radicals, such as multiconfigurational self-consistent field (MCSCF), complete active space perturbation theory to the 2nd order (CASPT2) and coupled-cluster theory. However, these methods are very computationally expensive, making them unsuitable for high-throughput workflows.^13–15^ Moreover, computationally cheaper methods such as conventional Time-Dependent Density Functional Theory (TD-DFT) can lead to spin-contaminated and functional-dependent results for the electronically excited states of radicals.^16,17^ Additionally, it has been shown that for the most accurate calculation of excited state energies one must also include nuclear quantum effects.^18^ Recently, an alternative, semiempirical method was developed—ExROPPP (Extended Restricted Open-shell Pariser–Parr–Pople theory), which is significantly faster, yet as accurate as higher level methods for calculating excited states of hydrocarbon radicals.^19^ ExROPPP is a based on the Pariser–Parr–Pople (PPP) Hamiltonian^20–24^ with a subsequent Extended Configuration Interaction Singles (XCIS)^25^ calculation which ensures spin purity. PPP theory has gained recent popularity for predicting electronic properties at a significantly reduced computational cost.^19,26–30^ Being a semiempirical method, PPP theory and consequently ExROPPP requires parameters which must be specified at the start of a calculation.^20–24^ The carbon atom PPP parameters already existing in the literature have been shown to be successful for predicting excited state energies for hydrocarbons in ExROPPP.^19^ However, emissive radicals commonly contain nitrogen and chlorine atoms, and we are not aware of any consistent, unified and widely-accepted set of parameters for including heteroatoms such as these in PPP theory or ExROPPP.^24,31,32^ The advent of ExROPPP has opened up the possibility of rapid screening of the electronically excited states of radicals, however, extending and generalizing this method requires an optimal set of parameters to be found.

In recent years Machine Learning (ML) has become an indispensable tool for the study of chemical systems.^33^ Such models allow for accurate prediction of chemical and physical properties with huge computational savings compared to methods such as DFT, provided sufficient data are available, and are often seen as an alternative to semiempirical and classical force-field methods.^34^ ML has seen numerous applications in predicting energies, structures and reactivity patterns of molecules and materials.^33–39^ Furthermore it has been applied to calculating the excited states of molecules and simulating excited-state potential energy surfaces.^34,40–46^ However, while a wealth of previous studies have been successful for closed-shell species, we find very few examples of ML for the electronically excited states of radicals. In recent work a machine-learned density functional, ML-ωPBE, previously trained on closed-shell species, was applied to calculate the electronically excited states of radicals in TD-DFT showing good accuracy with a mean absolute error (MAE) for excitation energies of less than 0.3 eV.^47^ However, to the best of our knowledge, learning the excited states of organic radicals directly from their experimental excited state data has yet to be attempted. Furthermore, typical ML models, in which no strong priors about the system are assumed at the outset, generally require large amounts of data, *e.g.* the properties of thousands of molecules or more, in order to be successful^33,34,36^ and we are unaware of any such large datasets for the excited states of radicals. In addition, predicting electronically excited states using ML is challenging as they are a largely non-local property which cannot in general be treated using atom-wise descriptors.^43^ Moreover, the prediction of primary outputs of quantum chemistry such as the *N*-electron wavefunction (and thus the composition of excited electronic states) is a highly desirable feature of an ML model, yet few ML models can predict these quantities for excited states.^43^ There has, however, been much recent interest in leveraging wavefunction-based descriptors in quantum ML models allowing them to retain some of the physical intuition of conventional quantum chemistry, and this work follows in a similar spirit to these developments.^48–52^

Due to the lack of sufficient excited state data available in databases for organic radicals and the aforementioned challenges, adopting a trusted physical model such as ExROPPP and learning its optimal parameters may be a viable alternative to conventional ML for the excited states of radicals.^19,26,27,53–59^ Using such a model also allows for the direct prediction of a variety of primary quantum chemical quantities such as molecular orbitals and transition dipole moments. In this paper we will focus on predicting molecular UV-visible linear absorption spectra and leave the computation of emission spectra, which usually requires excited state geometries that are difficult to acquire, for future research.

The linear UV-visible absorption spectra of organic radicals are usually characterised by two main features. These are an intense absorption (or absorptions) in the UV, usually between 300–400 nm, and a much weaker absorption in the visible (or near infra-red). The weak visible *D*_1_ state has been investigated using various levels of theory (TD-DFT, PPP, MCSCF).^2,9,19^ For alternant hydrocarbons (where the atoms can be divided into two groups such that no two atoms of the same group are directly bonded^60^), *D*_1_ is a minus combination |Ψ_*i*0_^−^〉 of the HOMO–SOMO and SOMO–LUMO excitations (where the SOMO is the singly-occupied molecular orbital) and is essentially dark in the absorption spectrum.^6,19,60–62^ Alternant hydrocarbons are usually non-emissive as non-radiative processes outcompete fluorescence.^6,18^ Conversely, in non-alternant molecules, the *D*_1_ state may have significantly higher absorption intensity. One widely explored class of non-alternant radicals are those with a donor–acceptor structure, such as TTM-1Cz, in which the *D*_1_ state is bright, charge transfer (CT) in nature and is mostly composed of the HOMO–SOMO excitation.^1,2,9^ These radicals are also highly emissive and have been incorporated into high-performing OLEDs.^1,2^ Another class of emissive radicals have recently been discovered which lack a donor–acceptor structure and CT characteristics, but instead employ mesityl groups leading to a large increase in the photoluminescence quantum yield. However, mesityl substitution does not significantly affect the absorption characteristics.^5^

In this paper we learn the excited states of organic radicals directly from their experimental data for the first time. We choose to train on experimental excited state data rather than the results of high-level calculations as we are unaware of any databases of high-level calculations (*e.g.* GMC-QDPT/CASPT2) of organic radicals, and also we aim to directly replicate experimental UV-visible spectra. To achieve this we use a modest amount of published UV-visible absorption data to learn an optimal set of ExROPPP parameters for organic radicals containing carbon, hydrogen, nitrogen and chlorine. Despite only containing a modest amount of data, we believe our compiled database of UV-visible absorption data of π-conjugated organic radicals to be the largest of its kind.^47^ Four new radicals are synthesised and we test our model on their absorption spectra to demonstrate its predictability and transferability. We find that the trained model has a significantly higher accuracy than the model using parameters taken from the literature and is able to make accurate predictions about the electronic excited states of unseen molecules.

## 2 Methodology

### 2.1 Data collection

**Fig. 1:**
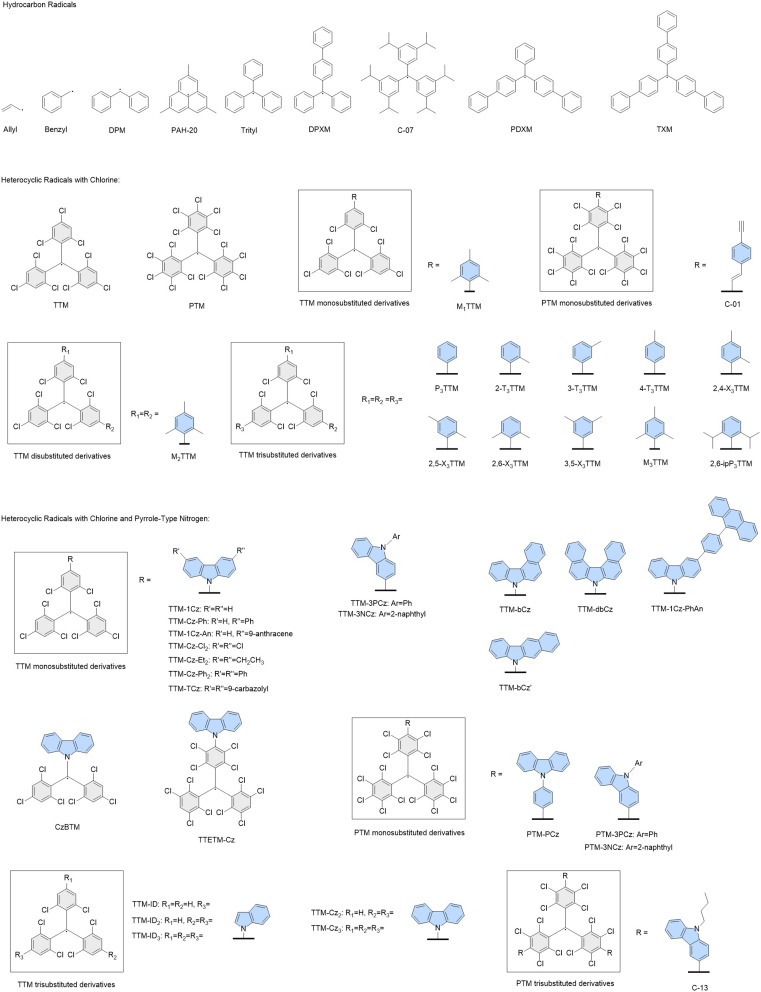
Structures of the molecules in the training set containing carbon, hydrogen, chlorine and pyrrole-type nitrogen. The central TTM/PTM backbone is coloured in grey, and substituents colored in light blue.

**Fig. 2:**
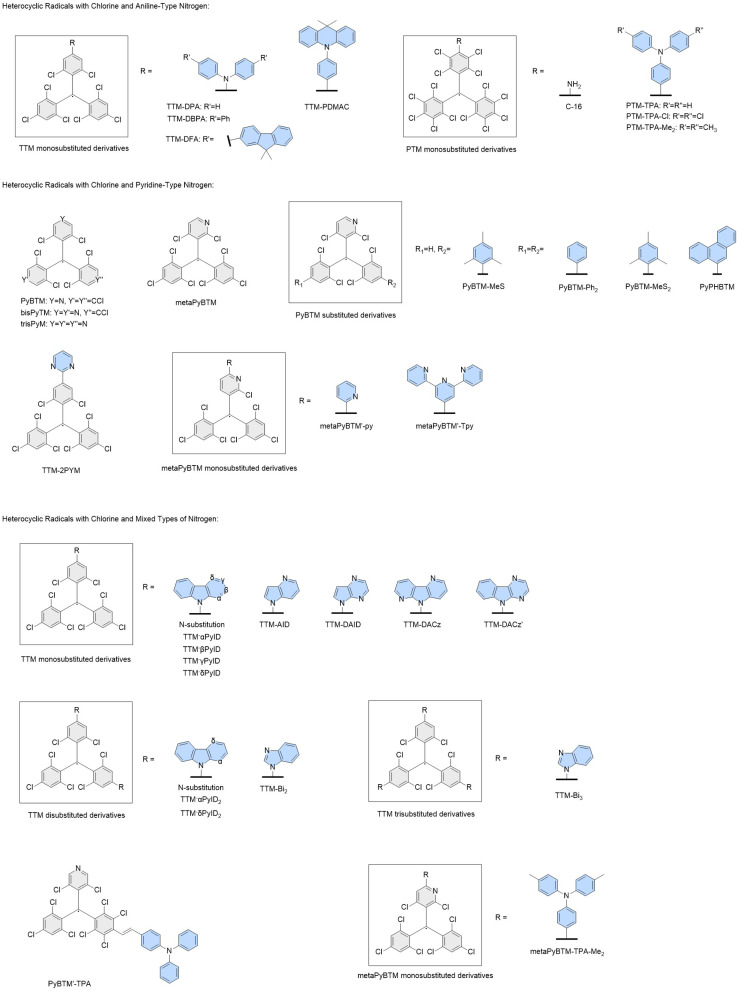
Structures of the molecules in the training set containing aniline, pyridine and multiple types of nitrogen. The central TTM/PTM backbone is coloured in grey, and substituents colored in light blue.

We obtained spectroscopic data for 81 organic radicals from previously published work whose structures are given in Fig. 1 and 2.^2,5–7,9,47,63–71^ In order to compile a database of suitable radicals, we considered all radicals we could find in the literature containing only carbon, hydrogen, chlorine and pyrrole, aniline and pyridine type nitrogen atoms. Those radicals whose spectroscopic absorption data could be found were added to the database along with their data. We also obtained DFT optimised molecular geometries for these molecules from previous studies.^2,5,9,47,64^ However, the molecular geometries for some molecules could not be found in the literature so most of these structures were optimised using unrestricted DFT with a gradient tolerance of 0.0001 E_H_/a_0_ in GAMESS-US.^72^ These data constitute the training set of the ML ExROPPP model. Further details pertaining to the collation of the database and geometry calculations can be found in SI Section I.

The target properties used for training are the energies *E*_*D*_1__ of the first excited doublet states (*D*_1_), the energies *E*_brt_ of the brightest absorptions in the UV-visible spectra, and 
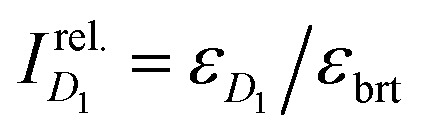
 which is the ratio of the molar extinction coefficients of these the two absorptions, extracted from linear UV-visible absorption spectra. These target properties are similar to those previously used in ML of molecular spectra.^34^ Exceptions are made for TTM-1Cz-An and TTM-1Cz-PhAn whereby due to their unusual electronic structure, their first excited doublet state (*D*_1_) is a dark triplet-coupled doublet state and the lowest energy bright doublet state is *D*_2_ which is by majority composed of the carbazole HOMO–TTM SOMO excitation (same orbital parentage as *D*_1_ in a typical donor–acceptor radical such as TTM-1Cz).^9^ Therefore, for these exceptional molecules we fit the *D*_2_ (instead of *D*_1_) energy and oscillator strength to the corresponding lowest energy *D*_2_ absorption seen in experiment. For these two molecules, we will group this state (*D*_2_) in with the *D*_1_ states for all the other molecules when performing the statistical analyses.

### 2.2 Training

We train the ExROPPP model on experimental UV-visible data of known organic radicals, using a fitness function of the computed energies and intensities compared to those obtained from experiment, which quantifies how well the predictions of the ExROPPP model fit with the experimental data. The fitness function takes the form1
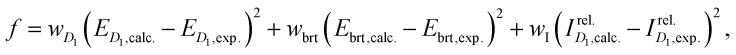
where *w*_*D*_1__, *w*_brt_ and *w*_*I*_ are the weights of the three respective terms in eqn (1). The weights of the first two terms have units of eV^−2^ and *w*_*I*_ is dimensionless such that *f* is a dimensionless quantity. While there are many other fitness functions which we could use, such as those based on the theory of optimal transport (between experimental and calculated spectra), we choose to use the above function as it encapsulates the essential spectral information of organic radicals which we believe is most important to be able to predict and only requires a small amount of raw spectral data for each radical.^73^

**Fig. 3:**
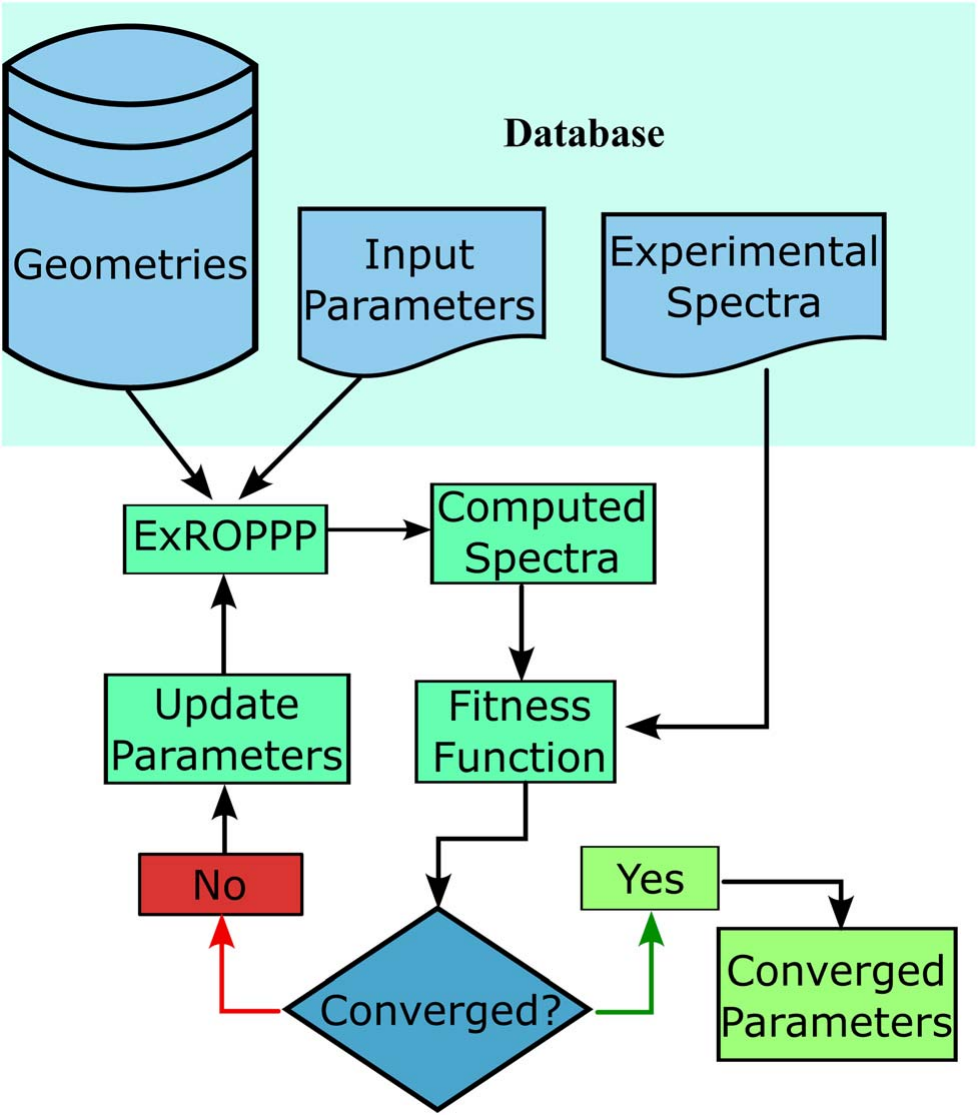
A flow diagram illustrating our method for training our ExROPPP model on the experimental absorption data of organic radicals.

Training is achieved by finding a set of ExROPPP parameters which minimises this fitness function utilising the derivative-free Nelder–Mead optimiser in Python as shown in Fig. 3.^74^ The algorithm first reads in the initial parameters, molecular geometries and experimental absorption data for all training molecules and classifies the molecules into hydrocarbons or heterocycles, which are treated slightly differently. For hydrocarbons, the fitness function comprises of only energy terms, with *w*_*D*_1__ and *w*_brt_ set to 1 eV^−2^ and *w*_*I*_ set to zero as the *D*_1_ state for hydrocarbons gives a very weak absorption in experiment and in ExROPPP has zero oscillator strength.^61^ For heterocycles, *w*_*D*_1__ and *w*_brt_ are set to 1 eV^−2^ and *w*_*I*_ set to 1 (except for a few molecules whose bright state data could not be found in which case *w*_*I*_ set to zero, see radicals-spreadsheet.xlsx in Data availability). Then the parameters are iteratively varied and the fitness calculated on each iteration until convergence.

We found that pre-training separately on subsets of molecules of different heteroatom types to obtain better initial guess parameters before training on all molecules for all parameters, an approach which we call ‘stratified’ training, leads to lower errors than not including these pre-training steps. The results of the trained model presented in the next section are obtained using this stratified approach. Details of the training process are discussed further in SI Section II. These calculations are parallelised for maximum efficiency.

### 2.3 Testing on novel radicals

Four new radicals: M_2_TTM-4T, M_2_TTM-3PCz, M_2_TTM-3TPA and M_2_TTM-4TPA, shown in Fig. 6 were synthesised, their UV-visible absorption spectra were measured, and their minimum energy geometries were obtained using DFT. The spectroscopic data (*E*_*D*_1__, *E*_brt_ and 
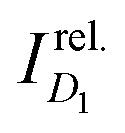
) and molecular structures of these new molecules form a testing set for the ML ExROPPP model. The molecular geometries and extracted UV-visible absorption data of all molecules as well as initial and optimised sets of parameters are available on the UCL Research Data Repository (see Data availability).

### 2.4 Statistical analysis

We calculate root mean-squared errors (RMSE), mean absolute errors (MAE), coefficients of determination *R*^2^ and Spearman's rank correlation coefficients (SRCC) between the experimental data and the simulated data for the training and testing set (see SI Section IV C).

### 2.5 PPP theory

Here we briefly introduce Pariser–Parr–Pople (PPP) theory before describing its extension to the ExROPPP method used as a surrogate model in this article.^20–22,75^

The PPP model extends semi-empirical Hückel theory by explicitly including π electron correlation, thereby reproducing the low-lying excited states of conjugated chromophores with markedly greater fidelity.^20–22,75^ The three core approximations that underpin PPP are: (i) σ–π separation, which treats the σ framework as an inert, non-polarisable core; (ii) zero differential overlap (ZDO), approximating the overlap matrix **S** as the identity matrix and neglecting three- and four-centre two-electron terms; and (iii) nearest-neighbour approximations, whereby the one-electron hopping integral *t*_*μν*_ is non-zero only for bonded pairs. To obtain the orbitals, Roothaan's equations are solved self consistently, which under ZDO reduce to:

2 **FC** = **CE**

with **F** the Fock matrix, **C** the molecular-orbital coefficient matrix, and **E** the diagonal orbital-energy matrix.^76^ Pople's formulation of PPP theory uses:^22^3
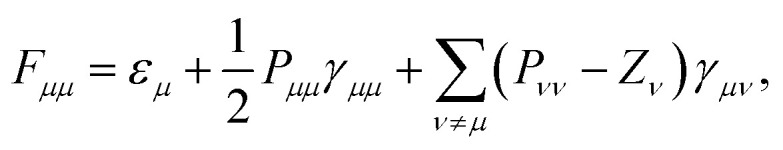

4 *F_μ_*_*ν*_ = *t_μ_*_*ν*_ − ½*P_μ_*_*ν*_*γ_μ_*_*ν*_.

where *ε_μ_* is the one-electron on-site Coulomb term (Hückel α) that captures the interactions of the π electron with the frozen σ core, *t_μ_*_*ν*_ is the hopping (resonance) integral (Hückel β) between neighbouring p orbitals, *γ_μμ_* and *γ_μ_*_*ν*_ are the one- and two-centre electron repulsion integrals that depend on the distance between atoms *μ* and *ν*, and *Z*_*v*_ is the effective nuclear charge.

### 2.6 PPP parameterisation

We employ largely the same functional form of PPP theory as in previous work.^19,24,26,27^

#### 2.6.1 Coulomb term

In this work, the Pariser–Parr form for *ε_μ_* is used, where it is initially taken as^20^

5 *ε_μ_* = −IP*_μ_*

where IP_*μ*_ is the valence-state ionization potential of atom *μ*.

#### 2.6.2 Two-electron integral

To parameterise the two-electron integral, the Mataga–Nishimoto form expressed in atomic orbitals is invoked:^24^6
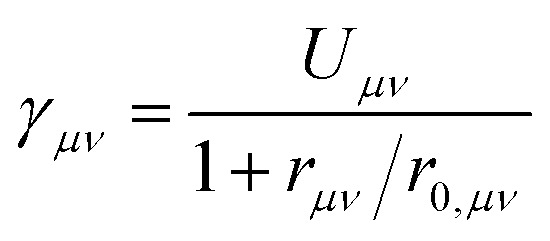
with *U_μ_*_*ν*_ = ½(*U_μμ_* + *U*_*νν*_) and *r*_0,*μν*_ = ½(*r*_0,_*_μμ_* + *r*_0,*νν*_). Here, *U*_*μ*_*_μ_* is the Hubbard on-site repulsion and *r_μ_*_*ν*_ is the scalar distance between atoms *μ* and *ν*. This formulation was used previously to describe the two-electron integrals in PPP theory for both closed-shell^26,27^ and radical^19^ systems.

#### 2.6.3 Hopping integral

Here we use a similar form of the hopping integral *t_μ_*_*ν*_ as used in previous studies,^19,26,27^ however unlike ref. 19, 26 and 27, but similar to ref. 77 we elect to use an exponentially decaying function for the hopping term. This term is also scaled by the cosine of the dihedral angle *θ* similar to ref. 19,^26^ and^27^ to accommodate non-planar geometries,

7 *t_μ_*_*ν*_ = *A_μ_*_*ν*_ exp(−*b_μ_*_*ν*_*r_μ_*_*ν*_)cos *θ*,

where *A_μ_*_*ν*_ and *b_μ_*_*ν*_ are parameters which are different for different pairs of atom types (see SI Section II).

#### 2.6.4 Effective nuclear charge

The PPP parameter *Z_μ_* is the effective nuclear charge (of the nuclei and core electrons) experienced by the π electron(s) on atom *μ*.^20,26^ We employ effective nuclear charges *Z_μ_* of *e* for carbon and pyridine type nitrogen as they each contribute one electron to the π-system, and 2*e* for chlorine and pyrrole/aniline type nitrogen as they each contribute two electrons to the π-system, where *e* is the electron charge.

#### 2.6.5 Atom specificity

The PPP parameter *ε*_*X*_ is atom-specific, whereas *t*_*XY*_ is atom pair-specific (where *X* and *Y* are now used to represent atom types rather than *μ* and *ν* which are atom indices). It would seem like *U*_*XY*_ and *r*_0,*XY*_ are also pair-specific, however, the convention of ref. 24 used here is that two-electron interactions between dissimilar atoms use arithmetic averages, *e.g. U*_*XY*_ = ½(*U*_*XX*_ + *U*_*YY*_) so there is only one unique *U*_*XX*_ and *r*_0,*XX*_ for every atom type *X*.

Different parameter sets are adopted for pyridine *versus* pyrrole/aniline nitrogens to reflect their different π–electron counts. Carbons and pyridine nitrogens donate one π electron, whereas chlorine and pyrrole/aniline nitrogens donate two *via* lone pairs. For further discussion of historical parameterisation strategies and alternative functional forms, see SI Section V.

### 2.7 ExROPPP method

Conceptually, ExROPPP combines the PPP Hamiltonian^20–22,75^ with a restricted open-shell ref. 60 and an extended configuration interaction singles (XCIS) scheme^25^ to rapidly calculate spin-pure electronically excited states with appreciable accuracy, as demonstrated for hydrocarbon radicals.^19^

#### 2.7.1 Restricted open-shell reference

Starting from a single-open-shell determinant |Ψ_0_〉 (with one Singly Occupied Molecular Orbital, SOMO), the molecular orbitals are optimised by minimising the total energy with respect to mixing of singly excited configurations,^19,59,60^ yielding the restricted open-shell Fock operator8
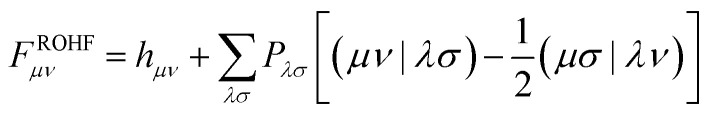
where *h_μ_*_*ν*_ is the one-electron integral and (*μν*|*λσ*) a two-electron integral in the atomic orbital basis. The density matrix elements are9
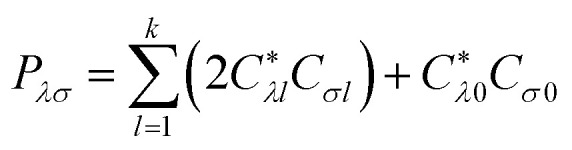
with *C*_*σl*_ the coefficient of atomic orbital σ that contributes to molecular orbital *l*, where *l* = 0 corresponds to the SOMO, *l* = 1 to the HOMO and so on.^19,59^ Diagonalising *F*^ROHF^ provides the optimised orbitals {*ϕ*_*p*_} used in the PPP ground-state calculation and subsequent integral evaluations.

#### 2.7.2 Spin-adapted excitation basis

Spin-pure doublet and quartet configurations are generated by linear combinations of single 
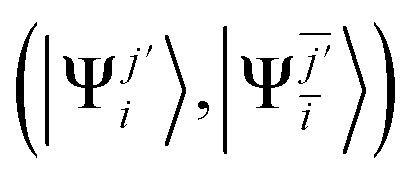
 and double 
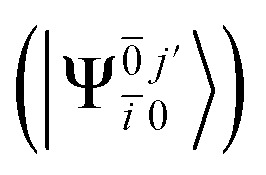
 excitations that are eigenfunctions of *Ŝ*^2^. For monoradicals with one unpaired electron in the SOMO, the spin-pure basis comprises:^19,59,60^

10 {|Φ_*I*_〉} = {|^2^Ψ_0_〉, |^2^Ψ_0_^*j*′^〉, |^2^Ψ^0^_*ī*_〉, |^2S^Ψ_*i*_^*j*′^〉, |^2T^Ψ_*i*_^*j*′^〉, |^4^Ψ_*i*_^*j*′^〉},

where |^2S^Ψ_*i*_^*j*′^〉, |^2T^Ψ_*i*_^*j*′^〉, |^4^Ψ_*i*_^*j*′^〉 are, respectively, the singlet-coupled, triplet-coupled and quartet states obtained by diagonalizing *Ŝ*^2^ matrix in the subspace spanned by 
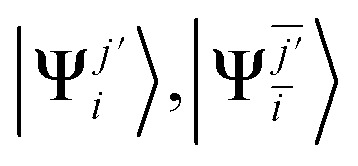
 and 
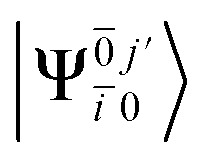
.

#### 2.7.3 XCIS Hamiltonian

Within this spin-pure basis the excited-state Hamiltonian is

11 **H**^XCIS^_*IJ*_ = 〈Φ_*I*_|*Ĥ*_PPP_|Φ_*J*_〉,

where *Ĥ*_PPP_ is built from the same one-electron on site terms *ε_μ_*, hoppings *t_μν_*, and two-electron Coulomb terms *γ_μ_*_*ν*_ as defined in Section 2.6. Block-diagonalisation of **H**^XCIS^ yields excitation energies and eigenvectors. See the SI eqn (5)–(8) for calculation of the transition dipole moments and oscillator strengths in the simulated spectra.

#### 2.7.4 Typical workflow

ExROPPP (i) solves the restricted open-shell Roothaan equations with the PPP parameters of Section 2.5 to obtain {*ϕ*_p_} and *E*_0_; (ii) constructs the spin-adapted XCIS manifold; (iii) forms and diagonalises **H**^XCIS^; and (iv) evaluates transition dipole moments. Absorption spectra are generated by applying Lorentzian broadening (FWHM 20 nm at a reference wavelength of 300 nm) to the discrete **H**^XCIS^ excitation energies.

## 3 Results

### 3.1 Training and validation

**Fig. 4:**
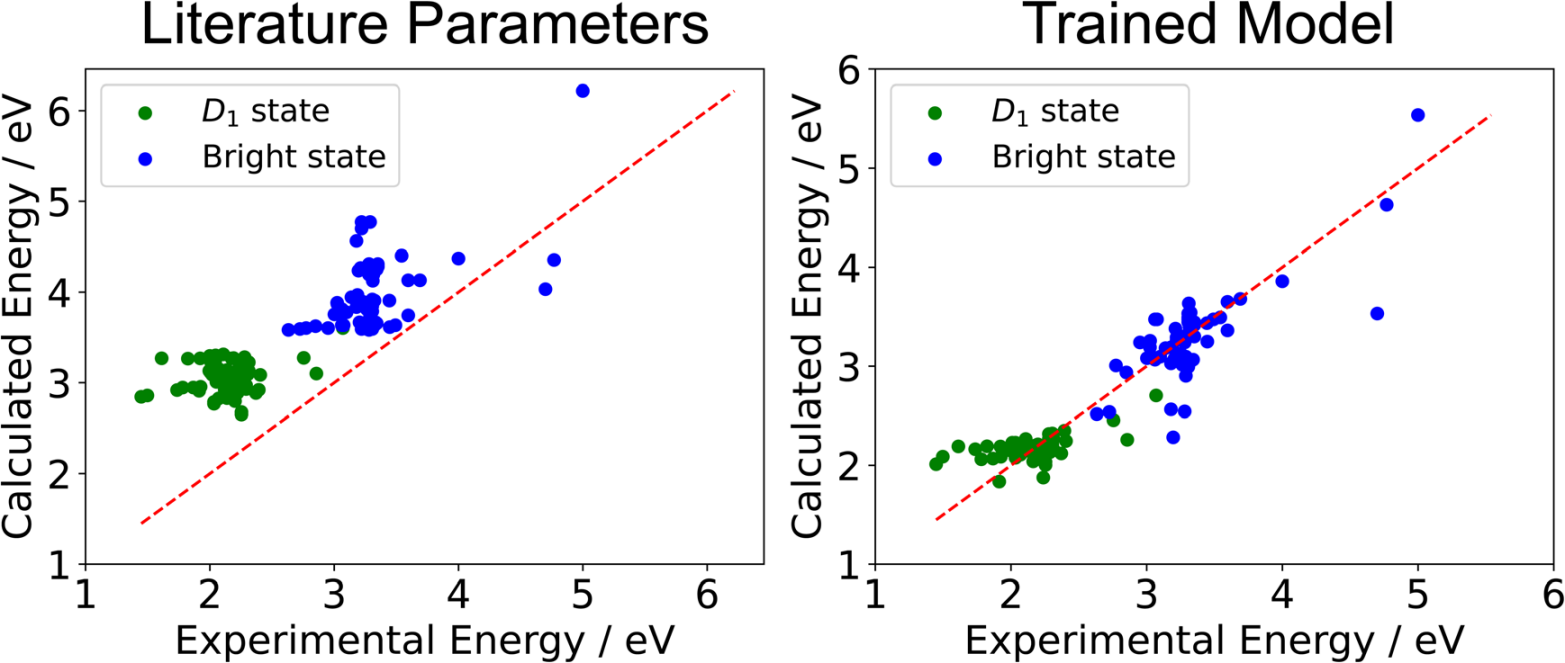
Regression plots of the excited state energies of the 81 molecule training set calculated by ExROPPP compared with experimentally determined energies, using parameters obtained from the literature (left) and those of the trained ExROPPP model (right). The trained model predicts the energies of UV/visible absorptions much closer to experiment (red line) than do the literature parameters.

The results of training the 81 molecule model are summarized in Fig. 4 and Table 1. We find that the accuracy of the simulated excited state energies improves significantly on training. The RMSE reduces from 0.86 eV with literature parameters to 0.24 eV for the trained model and the MAE reduces from 0.80 eV with literature parameters to 0.16 eV for the trained model. In terms of correlation, we find a marked improvement in *R*^2^ from −0.71 to 0.87 and a smaller improvement for SRCC going from 0.79 to 0.88 in the trained model compared with the literature parameters.

**Table 1:** Total fitness, RMSE, MAE, *R*^2^ and SRCC for the trained ExROPPP model and for the K-fold validation (total fitness for all folds, errors averaged across all folds) are compared to ExROPPP with parameters obtained from the literature, calculated for all states in the training set of 81 organic radicals. For further details on K-fold validation see SI Section III B

	Literature parameters	Trained model	K-fold model	Target
Total fitness	117.44	10.00	15.21	
RMSE (all states)/eV	0.86	0.24	0.27	<0.3
MAE (all states)/eV	0.80	0.16	0.18	<0.3
*R* ^2^ (all states)	−0.71	0.87	0.84	Close to 1
SRCC (all states)	0.79	0.88	0.86	Close to 1

**Fig. 5:**
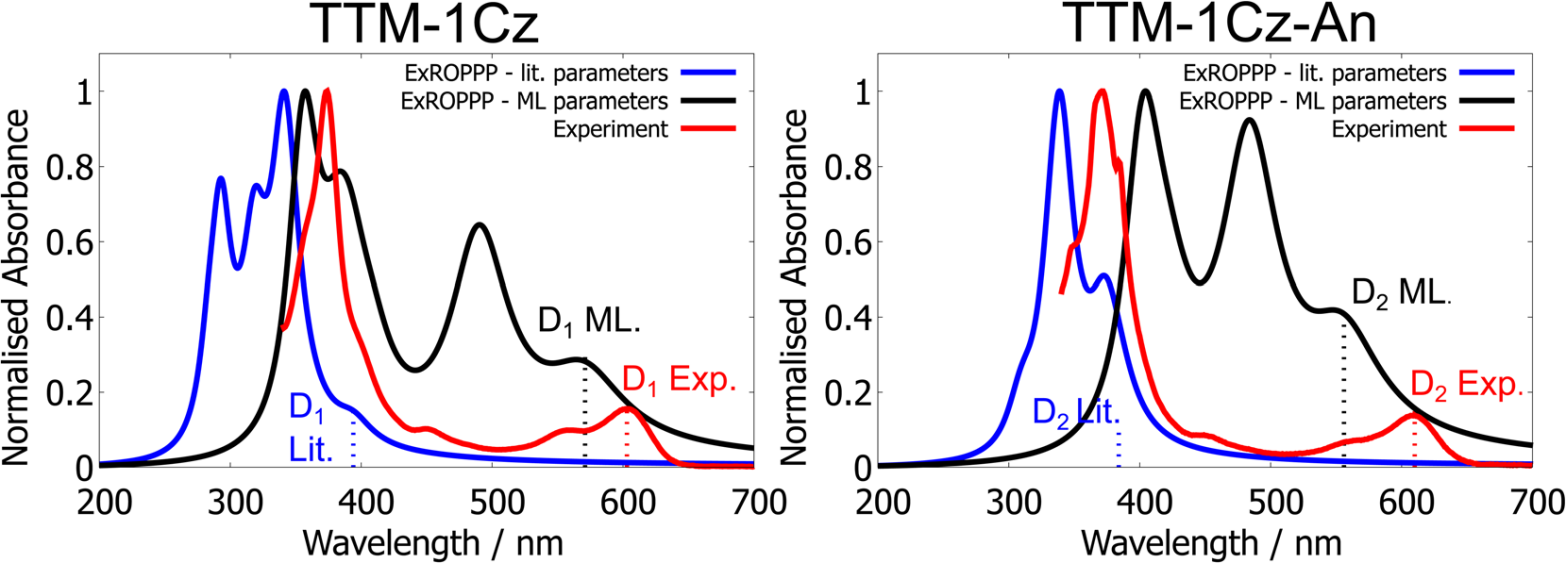
UV-visible absorption spectra of TTM-1Cz and TTM-1Cz-An measured in 200 μM toluene solution at room temperature (red), simulated using ExROPPP with literature parameters (blue) and with the trained 81 molecule model (black).^9^ The trained model substantially improves on the literature parameters in both cases.

The simulated spectra of the training set are more accurately reproduced by the trained model than by the model with literature parameters. To illustrate this we have included the spectra of two emissive radicals in the training set which are relevant to optoelectronics: TTM-1Cz and TTM-1Cz-An (see Fig. 5). TTM-1Cz is a prototypical and widely-studied emissive radical which has been implemented in functioning OLEDs, and is a good reference point for the trained ExROPPP model.^1^ On the other hand, TTM-1Cz-An is an atypical organic radical which has a complex and unusual electronic structure owing to its first excited state being a quartet and should be a challenging test case for ExROPPP. TTM-1Cz-An has been investigated for potential applications in quantum information technology.^9^

We find that the trained model reproduces the *D*_1_ (*D*_2_ for Cz-An) energies of these molecules significantly more accurately than does the literature parameters. The accuracy for the bright states also improves with the trained parameters. Furthermore, ExROPPP predicts that the quartet state *Q*_1_ of TTM-1Cz-An is lower in energy than the lowest energy bright state *D*_2_ in both sets of parameters, in line with experimental data and higher-level calculations in the literature. However, the energy of the *Q*_1_ state of TTM-1Cz-An in trained ExROPPP (1.10 eV) is quite far from the value obtained from higher-level calculations (2.26 eV in NEVPT2) which is not surprising given that ExROPPP was never trained on quartet state energies.^9^

Trained ExROPPP does produce an extra absorption around 500 nm for both molecules which is not seen in experiment, and this absorption arises due to ground state mixing of excited configurations (which is allowed for radicals as the form of ROHF we use does not exactly satisfy Brillouin's theorem).^19,25,59,60^ When this mixing is set to zero in the calculation, the states responsible for these extra absorptions have similar energies but now have almost zero intensity in the spectrum (see SI Fig. 3 and 4). Ground state mixing was not set to zero for the training process as the original implementation of ExROPPP included all Hamiltonian mixing terms and gave accurate energies and intensities for the excited states of hydrocarbon radicals.^19^ This being said, it is common practice in XCIS to set these elements to zero and training could be repeated without ground state mixing for more accurate intensities.^25^ It is also possible that the choice of fitness function allows a minimum point in the parameter space to be reached where the ground state mixing is too large and leads to inaccuracies in the predicted intensities of certain states in the UV-visible spectra. The fitness only depends on the energies and relative intensities of the *D*_1_ (*D*_2_ for Cz-An) state and the intense bright state and thus does not take into account other states in the spectrum. We leave further investigation of these spectral features for future research.

Despite the apparent inaccuracy for intensities, the ability of the trained ExROPPP model to accurately capture the excitation energies and excited state features of both typical and anomalous radicals shows its flexibility and robustness. In the SI Table XIV, we compute the fitness, MAE, *R*^2^ and SRCC including and excluding the two anomalous molecules (using the converged parameters from ML which includes the anomalous molecules) and find them to be extremely similar, indicating that their inclusion does not significantly affect the computed figures of merit.

In addition, we employed K-fold cross-validation on the 81-radical training set to validate the robustness of the model against data noise, using several different choices of folds. The average errors of the K-fold validation are similar to those for the whole training set in the trained model. In going from the trained model to the average K-fold model, the RMSE only slightly increases from 0.24 eV to 0.27 eV, the MAE goes from 0.16 eV to 0.18 eV, the *R*^2^ decreases slightly from 0.87 to 0.84 and the SRCC also decreases slightly from 0.88 to 0.86 (see Table 1). The data noise is also shown to be small for all trials. Artificially (selected by the user) and randomly distributed folds gave similar errors demonstrating that the distribution of molecules across folds does not significantly affect the model. Also, the K-fold results show that the stratified model has better robustness than the model without pre-training steps. Further details can be found in the SI Section III.

### 3.2 Transferability

Here we briefly consider the extent to which the trained ExROPPP model can reproduce the qualitative orbital structure of radicals. This is a particularly challenging test given that ExROPPP was not trained on orbital data.

A qualitative comparison of the singly occupied (SOMO) and highest occupied (HOMO) molecular orbitals from ExROPPP and Restricted Open-Shell DFT (B3LYP/6-31G(d,p)) demonstrates good overall agreement in both shape and localization, highlighting the transferability of the ExROPPP-based parametrisation. In TTM-1Cz, TTM-3NCz, and M_3_TTM, the SOMOs remain largely centered on the triarylmethyl carbon, with moderate extension onto the phenyl rings, whereas the HOMOs for TTM-1Cz and TTM-3NCz localize more strongly on the carbazole substituent. Notably, the local/global symmetry (*e.g.*, *C*_2_ for TTM-1Cz, *C*_3_ for M_3_TTM) and corresponding irreps are preserved. These observations underscore the physical interpretability and transferability of parameters derived *via* an optimizer-assisted physics-informed model, which can extrapolate untrained properties. Minor discrepancies occur for the M_3_TTM HOMO, where ExROPPP underestimates the small orbital coefficients in the outer ring. Further details and figures illustrating these points are provided in SI Section IV E.

### 3.3 Novel organic radicals

**Fig. 6:**
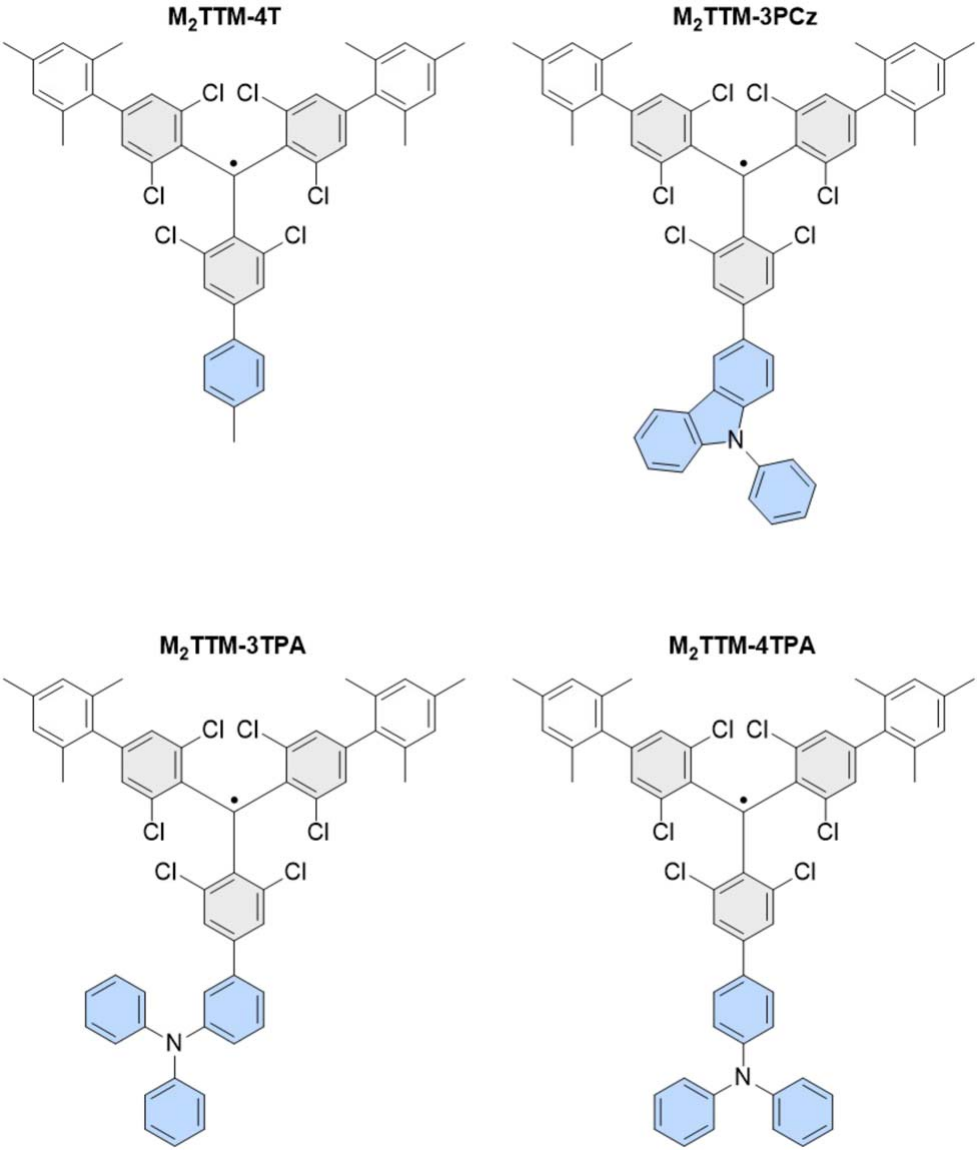
Structures of the four newly synthesised radicals reported in this work: M_2_TTM-4T, M_2_TTM-3PCz, M_2_TTM-3TPA and M_2_TTM-4TPA, which constitute the testing set.

To test our model, we synthesized four novel trityl radicals, specifically designed to probe various state-of-the-art concepts previously identified in mono-radical systems (see Fig. 6). Each radical was based on a mesitylated TTM framework, which has been shown to enhance photoluminescence quantum efficiency (PLQE) by augmenting the radiative decay rate.^5^ To evaluate the ExROPPP model with an asymmetric structure and the absence of charge transfer (CT), toluene was appended to the unsubstituted site of the mesitylated trityl radical core through its 4-position. The three other radicals incorporated CT groups, namely 9-phenylcarbazole (PCz) and triphenylamine (TPA), which contain non-bonding nitrogen lone pairs. These non-bonding electrons have been shown to enhance photoluminescent efficiency through a reduction in excitonic coupling to high-frequency vibrational modes.^78^ Through the inclusion of PCz and TPA moieties, we aimed to test the model across electron-donating groups of varying strengths, with TPA being the stronger donor due to the hybridization of its nitrogen heteroatom which influences lone pair availability. Additionally, TPA units were linked to the trityl radical core through both the 3- and 4-positions to assess accuracy in predicting spectroscopic outcomes for different stereoisomers. The combination of mesitylation and non-bonding CT groups provides a promising strategy for developing highly efficient radical emitters and it is crucial that the ExROPPP method can predict outcomes for these cutting-edge radical designs.

The synthesis of the radical species commenced with the formation of αHM_2_TTM as previously reported by Murto *et al.*^5^ Following this, αHM_2_TTM was reacted with the respective 3- and 4- linked boronic acids of PCz and TPA to form αHM_2_TTM-3PCz and αHM_2_TTM-4TPA. To create the αH precursors for the other two radical species, the remaining para-chlorine of αHM_2_TTM was converted to a boronic ester through a Miyaura borylation before being coupled with 4-iodotoluene or 3-bromotriphenylamine. To convert into their respective radicals, all four αH species were subjected to tetrabutylammonium hydroxide, to form the monoanion, before being oxidised to the radical using *para*-chloranil. M_2_TTM-4TPA, M_2_TTM-3TPA, M_2_TTM-3PCz and M_2_TTM-4T were formed in a 13%, 56%, 86% and 37% yield respectively. UV-vis absorption measurements were carried out for the radicals in a 0.1 mM toluene solution. All four radicals display an intense absorption feature around 370–400 nm, which is characteristic of a local excitation within the TTM radical core. For M_2_TTM-3PCz and M_2_TTM-4TPA, additional absorption peaks can be seen at 590 and 630 nm respectively. These are attributed to CT transitions between the electron donating group and the electron-accepting TTM core.

**Fig. 7:**
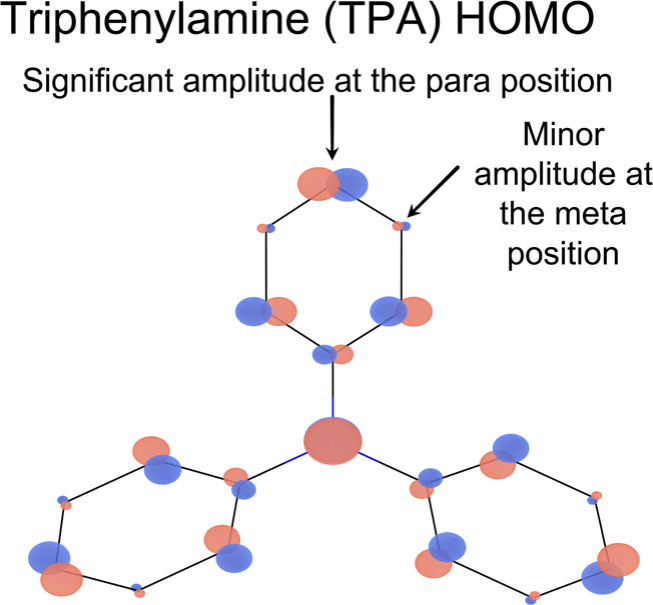
HOMO of TPA calculated by closed-shell PPP (with the optimised parameters obtained from training on 81 radicals). There is significant HOMO amplitude at the *para* (4) position but minimal amplitude at the *meta* (3) position, such that the design rules correctly predict M_2_TTM-4TPA to have a significant low-energy visible absorption and M_2_TTM-3TPA not to have one.

**Fig. 8:**
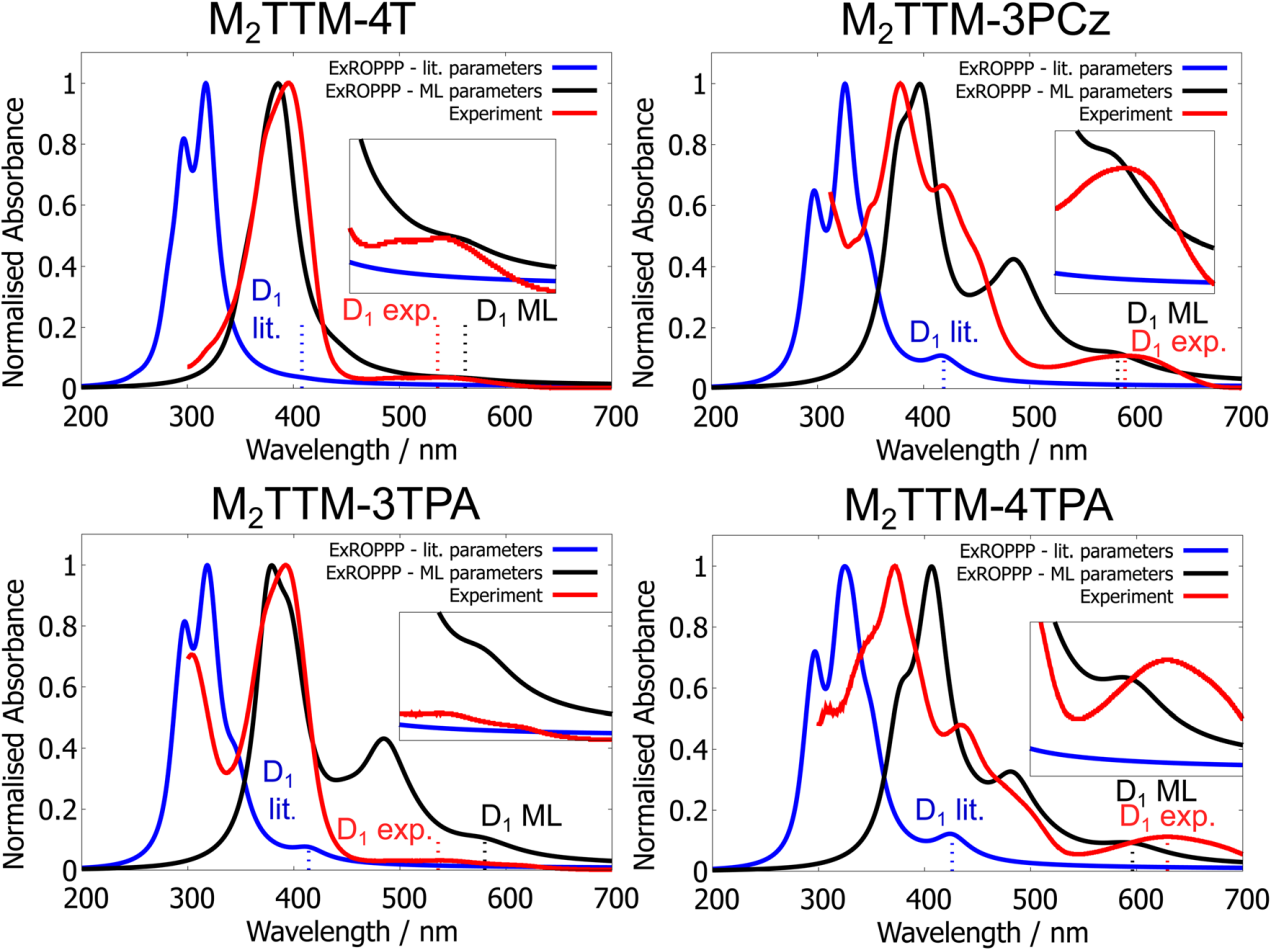
UV-visible absorption spectra of the newly-synthesised M_2_TTM-4T (top left), M_2_TTM-3PCz (top right), M_2_TTM-3TPA (bottom left) and M_2_TTM-4TPA (bottom right) measured in 0.1 mM toluene solution (red), simulated using ExROPPP with literature parameters (blue) and of the trained 81 molecule ExROPPP model (black). Trained ExROPPP (black) reproduces the experimental spectra (red) more accurately than untrained ExROPPP with literature parameters (blue).

We find that the four new molecules confirm the structure–property predictions made in 2020 (ref. 6) that, in order for a significant *D*_1_ absorption the molecule should not be an alternant hydrocarbon and that the HOMO on the donor (4T, 3PCz or TPA in this case) has orbital amplitude on the atom through which it is joined to the acceptor (TTM). M_2_TTM-4T is predicted to have minimal *D*_1_ oscillator strength as it is a *de facto* alternant hydrocarbon, as is observed experimentally. M_2_TTM-3PCz contains a five-membered ring and a nitrogen, both of which break alternacy symmetry leading to a bright *D*_1_ state, as is experimentally observed. A simple PPP calculation on TPA alone finds that the HOMO has significant amplitude at the *para* (4) position but minimal amplitude at the *meta* (3) position, as shown in Fig. 7. This therefore predicts that the TPA to TTM charge transfer excitation will be dark in M_2_TTM-3TPA but bright in M_2_TTM-4TPA, as is observed experimentally in Fig. 8. We believe this is the first direct experimental confirmation of the design rule concerning the HOMO amplitude.

### 3.4 Testing

**Fig. 9:**
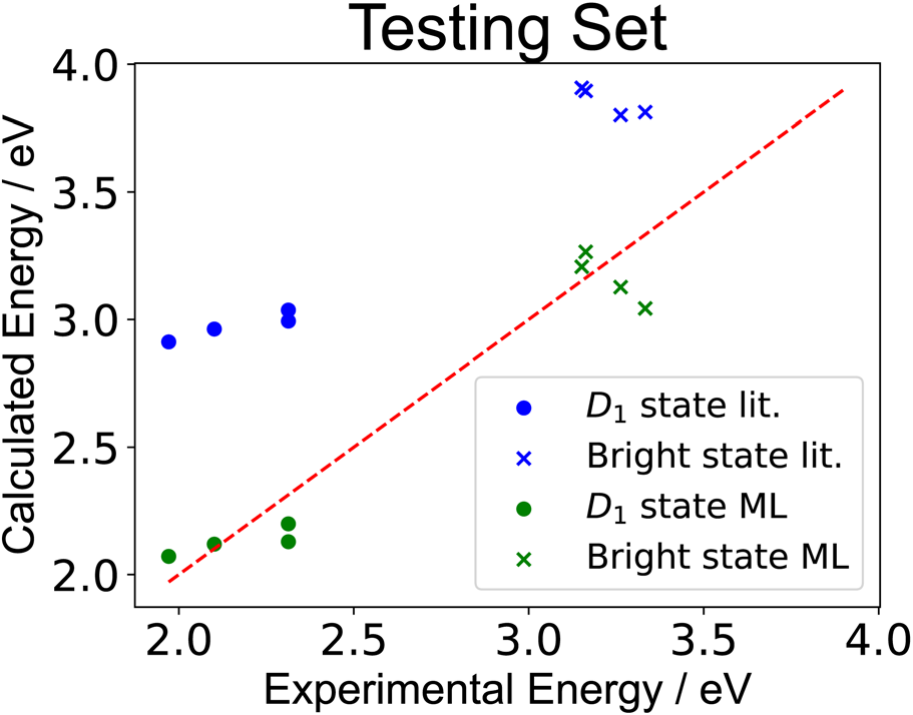
Regression plot of the excited state energies of the radicals in the testing set calculated by ExROPPP and compared with experimentally determined energies. ‘lit.’ refers to the parameters sourced from the literature and ‘ML’ refers to the parameters of the trained 81 molecule model. It can be clearly seen that for this testing set the trained ExROPPP model more accurately reproduces the experimental values than ExROPPP with literature parameters.

We tested the trained 81-molecule model on our four new organic radicals: M_2_TTM-4T, M_2_TTM-3PCz, M_2_TTM-3TPA and M_2_TTM-4TPA shown in Fig. 6, which make up the testing set. The same parameters optimised for the 81 radicals in the training set in Section 3.1 (presented in SI Table XII) are applied to the four testing molecules here, and are not re-trained or altered in any way for the testing set. We find that the trained model performs well on the testing set, predicting both *D*_1_ and bright state energies with a significantly higher accuracy than the literature parameters, as can be seen in Fig. 9. We also calculated the RMSE, MAE, *R*^2^ and SRCC for the testing data, presented in Table 2. We find similar values for the errors and correlation metrics for the testing set as seen previously for the training set, again with RMSE and MAE less than 0.3 eV and *R*^2^ and SRCC of 0.93 and 0.76 respectively. The fact that the errors (RMSE and MAE) for the testing set of the four newly-synthesised molecules in Table 2 (which the model was never trained on) are actually slightly lower than for the training set (both for the learning and cross-validation in Table 1) further indicates that overfitting did not occur.

**Table 2:** RMSE, MAE, *R*^2^ and SRCC for the testing set of four newly synthesised molecules: M_2_TTM-4T, M_2_TTM-3PCz, M_2_TTM-3TPA and M_2_TTM-4TPA, using (i) ExROPPP with literature parameters (ii) ExROPPP with parameters trained on the training set of 81 molecules. For RMSE, MAE and *R*^2^ the trained model vastly improves on the literature parameters, and the SRCC is unchanged

	Literature parameters	Trained model	Target
RMSE (all states)/eV	0.73	0.15	<0.3
MAE (all states)/eV	0.71	0.13	<0.3
*R* ^2^ (all states)	−0.83	0.93	Close to 1
SRCC (all states)	0.76	0.76	Close to 1

We also compare the ExROPPP simulated UV-visible absorption spectra, with both literature and trained parameters, with the experimental spectra for these four molecules as shown in Fig. 8. The simulated spectra of all four unseen molecules are significantly improved after training and overall the trained model accurately reproduces their absorption spectra. However, the spectrum of M_2_TTM-3TPA is slightly less well captured by ExROPPP than for the other three molecules, where it predicts a larger *D*_1_ intensity and lower *D*_1_ energy than seen in experiment. Similar extra absorptions are also seen around 500 nm for M_2_TTM-3PCz, M_2_TTM-3TPA and M_2_TTM-4TPA, as seen for TTM-1Cz and TTTM-1Cz-An, but are less pronounced. This again highlights that while very accurate at reproducing excitation energies, the trained model is less accurate at predicting intensities of the UV-visible absorptions.

## 4 Conclusions

In this article we have presented the first demonstration of learning the electronically excited states of radicals from experimental data. We achieve this by using the spin-pure ExROPPP method as a surrogate model, both to avoid the spin-contamination problem, and to address the limited experimental data in the literature. We find that the trained ExROPPP model performs far better at computing spectral features of organic radicals than the literature parameters. Four new radicals are synthesised and we test our model by comparing computed spectra against experimental data, finding good agreement and demonstrating its wider applicability as a predictive model. While the trained model accurately reproduces excitation energies, it seems to be less accurate at predicting intensities when compared to experimental UV-visible spectra. More generally, this research shows that ML can be effective with sparse data when using a careful choice of surrogate model.

In future work the accuracy for UV-visible intensities predicted by ExROPPP could possibly be improved by removing ground state mixing in the ExROPPP Hamiltonian and employing an alternative fitness function. This model could also be further extended to predicting the emission spectra of radicals, and also to other atoms and groups common in organic radicals such as O, S and F, nitrile, nitro, aminoxyl and trifluoromethyl.^63^ Moreover, while ExROPPP is limited to π-conjugated organic radicals, by incorporating the equations of XCIS into an extended Hückel method such as ZINDO, an analogous but more general approach could be formulated which would allow, for example, for the spin-pure excited states of radicals containing heavy atoms such as transition metal complexes to be rapidly simulated.^79^ Taken together, this work serves as a major step forward for high-throughput screening and inverse molecular design of radicals with applications in OLEDs and qubits.

## 5 Experimental methods

### 5.1 Characterization techniques of organic radicals

NMR spectra were acquired using a 400 MHz Bruker Avance III HD spectrometer (^1^H, 400 MHz; ^13^C, 100 MHz). Chemical shifts are reported in *δ* (ppm) relative to the solvent peak: chloroform-d (CDCl_3_: ^1^H, 7.26 ppm; ^13^C, 77.16 ppm) and dichloromethane-d_2_ (CD_2_Cl_2_: ^1^H, 5.32 ppm; ^13^C, 53.84 ppm). Mass spectra were obtained on a Waters Xevo G2-S benchtop QTOF mass spectrometer equipped with a electrospray ionization (ESI) or an atmospheric solid analysis probe (ASAP). Flash chromatography was carried out using Biotage Isolera Four System and Biotage SNAP/Sfär Silica flash cartridges.

### 5.2 Steady-state UV-visible spectroscopy

UV-visible spectra were measured with a commercially available Shimadzu UV-1800 spectrophotometer.

## Author contributions

TJHH conceived the research idea with assistance from JDG and JS. Computational research for training the ExROPPP model was undertaken by JDG, JS and KM, supervised by TJHH. Organic synthesis, characterization and measurement of UV-visible absorption spectra was performed by LEW, supervised by HB. JS, KM, LEW and JDG all contributed to DFT calculations. The orbital visualiser was written by KM. KB advised on machine learning. The initial draft of the manuscript was written by JDG with assistance from TJHH, JS and KM and the organic synthetic parts written by LEW. All authors contributed to the revising and reviewing of the manuscript.

## Conflicts of interest

There are no conflicts to declare.

## Supplementary Material

SC-OLF-D5SC04276C-s001

## Data Availability

The extended data including the molecular geometries and literature UV-visible absorption data for all molecules, and initial and optimised sets of parameters are available at the UCL Research Data Repository at https://rdr.ucl.ac.uk/articles/dataset/Research_data_for_Learning_Radical_Excited_States_from_Sparse_Data_/29457698/1. Details of database collation, the training and validation processes, optimized parameters, parameterization schemes and experimental synthesis and spectra. See DOI: https://doi.org/10.1039/d5sc04276c.
