# Supplementary material for: Learning radical excited states from sparse data
Source: Chem Sci. 2025 Aug 12;16(37):17356–68. doi: 10.1039/d5sc04276c (PMC12400446; doi:10.1039/d5sc04276c)
Supplement: SC-016-D5SC04276C-s001 [file SC-016-D5SC04276C-s001.pdf]

Supplementary Information (SI) for Chemical Science.
This journal is © The Royal Society of Chemistry 2025

Supplementary Information for: Learning Radical Excited States from Small Data

Jingkun Shen,[1] Lucy E. Walker,[2] Kevin Ma,[1] James D. Green,[1] Hugo Bronstein,[2] Keith T. Butler,[1] and Timothy J. H. Hele[1, a)]

[1)] *Department of Chemistry, Christopher Ingold Building, University College London, WC1H 0AJ, UK.*

[2)] *Yusuf Hamied Department of Chemistry, University of Cambridge, CB2 1EW, UK; Department of Physics, Cavendish Laboratory, Cambridge University, Cambridge CB3 0HF, UK.*

(Dated: 31 July 2025)

[a)] Electronic mail: t.hele@ucl.ac.uk

CONTENTS

I. Database Collation 3

A. Truncation of Geometries 6

II. Training 7

A. Initial Parameters 7

B. Training Strategies 9

C. Optimization Process 11

D. Generating Absorption Spectra for Target Variables 13

1. Details of simulation of spectra 13

2. Extra absorptions in simulated spectra 14

3. Contraction of spectra 15

III. Validation 18

A. K-fold Validations 18

B. K-fold Results 22

IV. Results and Analysis 28

A. Optimized parameters 28

B. Analysis of the optimized parameters 30

1. Optimized ε Terms (Core Hamiltonian) 31

2. Optimized U Terms (On-Site Electron Repulsion) 32

3. Resonance Integrals $t_{\mu\nu}$ 32

C. Mathematical Expressions for Statistical Metrics 33

1. Root Mean Square Deviation (RMSD) 33

2. Mean Absolute Error (MAE) 34

3. Spearman Rank Correlation Coefficient (SRCC) 34

4. Coefficient of Determination (R^2) 35
D. Results of the trained ExROPPP model 36
E. ExROPPP Simulated Molecular Orbitals 39

V. ExROPPP and PPP Parameterisation 42
A. PPP Parametrization Scheme 42
B. One-electron Integrals 43
C. Two-electron Repulsion Integrals 44

VI. Experimental Section 46
A. Materials and synthesis 46
B. NMR Spectra 57

References 88

I. DATABASE COLLATION

In this study, we developed a database of 81 radicals, including their chemical structures, optimized geometries, and experimental spectroscopic data primarily taken from a review by Mizuno et al.[1] and the recent work of Cheng-Wei Ju et al.[2], as well as from several other papers including our recent work on hydrocarbon radicals.[3–16] We initially selected all radicals we could find experimental absorption data for containing hydrogen, carbon, chlorine, and nitrogen atoms typical of pyrrole, aniline, and pyridine. Some radicals were reported in more than one of these papers, and in these cases we chose the paper which contained as much of the desired data for the training process (E_{D_1}, E_{brt}, ϵ_{D_1}, ϵ_{brt}) as possible. Where possible, we extracted data from raw spectroscopic data files (.csv) or from quoted values, however, in some cases where this data was absent but a figure of a UV-visible absorption

spectrum was present in the paper, the data was estimated by reading off from the figure.[1,2,14,17]

There are two sources for the molecular geometries of the radicals: i) they were optimised and reported in other literature, so we used them in our paper citing the relevant papers (the papers from which the optimised geometries were taken are given in the extended dataset in ”lit_exp_uvvis_data.xlxs”, see data availability) or ii) we performed geometry optimisations of these monoradicals using the Unrestricted B3LYP method with a 6-31G(d,p) basis set, maintaining a gradient tolerance of 0.0001 Hartree/Bohr, using the GAMESS-US software.[18] The details of methods and basis sets that were used for all radicals are also included in the ”lit_exp_uvvis_data.xlxs” spreadsheet in the data repository. Exceptions, detailed in Table I, were sourced from existing literature and optimized using the PBE0 method. Some structures required a higher gradient convergence tolerance of 0.001 Hartree/Bohr due to their larger size and complexity, which posed challenges for convergence. The geometry optimisations of some of the radicals (TTM-BiCz, TTM-BiCz$_2$, TTM-3PDMAC, PTM-TPA-MeCl) could not be converged due to their large and highly flexible structures, so they were excluded from the database. A further two molecules (chlorobenzyl and DABCz) were omitted from the database as they suffered from issues during the training process. The peak selection algorithm failed for chlorobenzyl as sets of parameters mid-optimisation led to a spectrum with no absorptions in the region of the spectrum of 250-650 nm being searched, and the SCF procedure would consistently fail to converge for DABCz at all stages of training. This left 81 radicals added to the final database and subsequently used in the training process. Details of the final 81 molecule database, including sources of data for each individual molecule, details of the initial selection process and molecules omitted from the database are also given in the extended dataset (see Data Availability

in the main text).

TABLE I: Exception Geometries

Name	Method	Basis Set	Tolerance Hartree / Bohr
C-07	UPBE0	6-31G(d)	Not reported
PAH-20	UPBE0	6-31G(d)	Not reported
C-01	UPBE0	6-31G(d)	Not reported
TTM-ID_2	UPBE0	6-31G(d)	Not reported
TTM-ID_3	UPBE0	6-31G(d)	Not reported
PTM-3PCz	UPBE0	6-31G(d)	Not reported
C-13	UPBE0	6-31G(d)	Not reported
C-16	UPBE0	6-31G(d)	Not reported
M_1TTM	UB3LYP(D3)	def2-SVP	Not reported
M_2TTM	UB3LYP(D3)	def2-SVP	Not reported
M_3TTM	UB3LYP(D3)	def2-SVP	Not reported
P_3TTM	UB3LYP(D3)	def2-SVP	Not reported
4-T_3TTM	UB3LYP(D3)	def2-SVP	Not reported
3-T_3TTM	UB3LYP(D3)	def2-SVP	Not reported
2-T_3TTM	UB3LYP(D3)	def2-SVP	Not reported

Table I continued on next page ...

Table I continued from previous page ...

Name	Method	Basis Set	Tolerance
3,5-X_3TTM	UB3LYP(D3)	def2-SVP	Not reported
2,6-X_3TTM	UB3LYP(D3)	def2-SVP	Not reported
2,5-X_3TTM	UB3LYP(D3)	def2-SVP	Not reported
2,4-X_3TTM	UB3LYP(D3)	def2-SVP	Not reported
2,6-ipP_3TTM	UB3LYP(D3)	def2-SVP	Not reported
M_2TTM-4Me	UB3LYP(D3)	def2-SVP	Not reported
M_2TTM-3PCz	UB3LYP(D3)	def2-SVP	Not reported
M_2TTM-4TPA	UB3LYP(D3)	def2-SVP	Not reported
M_2TTM-3TPA	UB3LYP(D3)	def2-SVP	Not reported
CzBTM	UM06-2X	6-31G(d,p)	Not reported
TTM-1Cz-An	UM06-2X	6-31G(d,p)	Not reported
TTM-1Cz-PhAn	UM06-2X	6-31G(d,p)	Not reported
TTM-3PCz	UB3LYP	6-31G(d)	Not reported
TTM-3NCz	UB3LYP	6-31G(d)	Not reported
TTM-bCz	UB3LYP	6-31G(d,p)	0.001
TTM-bCz'	UB3LYP	6-31G(d,p)	0.001
TTM-Cz-Ph	UB3LYP	6-31G(d,p)	0.001

A. Truncation of Geometries

As the Pariser-Parr-Pople method is constrained to π-systems only, we truncated sp^3 carbon groups from the optimised structures for 25 of the radicals in the database, which are listed in the data availability file (See Data Availability in the main text).

Even though some of the radicals have the same 2D structure after truncation, the truncated 3D structures are different (e.g. have different dihedral angles) because of the influence of the truncated parts in the geometry optimisation.

II. TRAINING

A. Initial Parameters

A set of initial parameters were chosen by taking the carbon parameters used in previous work[3,19], and using the Beveridge and Hinze[20] core ϵ_X and Hubbard U_{XY} parameters and the Mataga-Nishimoto[21] r_0 parameters. The exponential hopping A_{CC} and b_{CC} parameters for carbon were sourced from Ref. 22. For C-N1 (pyridine-type), C-N2 (pyrrole/aniline-type) and C-Cl bonds, the A_{XY} parameters were fitted to the t_{XY} values obtained from Ref. 21 (for C-N1) and Ref. 23 (for C-N2 and C-Cl) using the same b_{XY} values as for carbon and with average bond lengths of 1.333877 Å, 1.410367Å and 1.749588Å for C-N1, C-N2 and C-Cl bonds respectively.

TABLE II: Literature PPP parameter set used for training using exponentially decaying hopping term.

Parameter	Value	Unit	Reference
ϵ_{C}	0	eV	20
ϵ_{N1}	-2.96	eV	20
ϵ_{N2}	-17.56	eV	20
ϵ_{Cl}	-12.65	eV	20
A_{CC}	-28.08	eV	22
A_{CN1}	-23.54	eV	21,22
A_{CN2}	-22.16	eV	22,23
A_{CCl}	-27.10	eV	22,23
b_{CC}	1.66	Å^{-1}	22
b_{CN1}	1.66	Å^{-1}	22
b_{CN2}	1.66	Å^{-1}	22
b_{CCl}	1.66	Å^{-1}	22
U_{C}	8	eV	3,19
U_{N1}	12.34	eV	20
U_{N2}	16.76	eV	20
U_{Cl}	8	eV	Approximated to be the same as C value
$r_{0,\mathrm{C}}$	1.328	Å	3,19,21
$r_{0,\mathrm{N1}}$	1.115	Å	21
$r_{0,\mathrm{N2}}$	1.115	Å	Approximated to be the same as for N1
$r_{0,\mathrm{Cl}}$	1.987	Å	Approximated using Cl-Cl equilibrium bond length from 24

B. Training Strategies

In the optimization of parameters within the ExROPPP framework for predicting the excited states of organic radicals, three distinct training strategies were employed to determine the best parameters:

Brute Force Training: Parameters were directly optimized using the complete dataset of 81 radicals.

Stratified Informed Training: Our multi-step "stratified informed" strategy begins by training the PPP parameters on a smaller dataset consisting solely of hydrocarbons. Next, we extend the training set to include monoradicals that contain carbon, hydrogen, and chlorine (CHCl). Subsequently, we introduce nitrogen-containing molecules, which we subdivide into three categories: aniline-type, pyridine-type, and pyrrole-type. Each nitrogen subset is trained independently (e.g., via Nelder–Mead), producing three sets of optimized parameters. We then combine these three sets into a single comprehensive parameter set using a stratified weighting scheme:

$$w_i = N \times \frac{\text{number of molecules of type } i}{\text{total number of molecules}}. \tag{1}$$

Here, N is a normalization constant ensuring that $\sum w_i = 1$. The factor w_i represents the weight assigned to the parameters derived from the subset of molecules of type-i. Figure 2 provides an overview of this procedure.

During optimization, we found that including TTM-DAID and TTM-DACz' from the outset consistently led to Fock-matrix convergence failures, which disrupted the Nelder–Mead search. To circumvent these issues, we initially excluded those two molecules from the training set. After stabilizing the parameter refinement in their absence, we tested multiple reintroduction schedules—e.g., after 500, 1000, 1500, 2000, 2500, or 3000 iterations—to determine the optimal balance between robust convergence and retaining their critical data. These schedules are summarized in

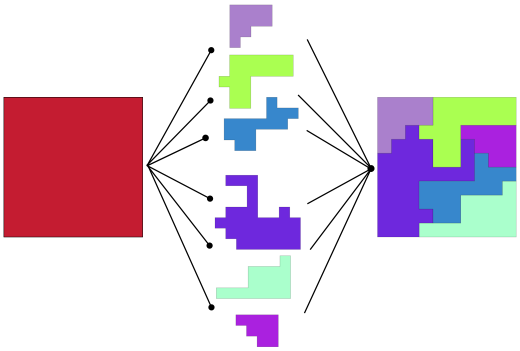

FIG. 1: Schematic representation of the divide and conquer process: Partitioning into six classes (middle), independent optimizations (left), and integration into a unified parameter (right).

Table III.

Divide and Conquer: A complementary "divide and conquer" approach partitions the full dataset into six non-disjoint molecular classes (Figure 1). We first optimize the PPP parameters independently within each class, thus obtaining six class-specific parameter sets P_i. These are then combined into a single unified set P^* according to:

$$P^* = \sum_{i}^{6} w_i P_i \tag{2}$$

$$w_i = \frac{\text{population of non-disjoint type i}}{\text{total population}} \tag{3}$$

We examined two variations on how to finalize P^*. First, each class was assigned an equal weight w_i=1/6, and the resulting aggregate was refined with Nelder-mead optimizer. Second, the weights were taken to be the actual population fractions, ensuring classes with more molecules had proportionally greater influence.

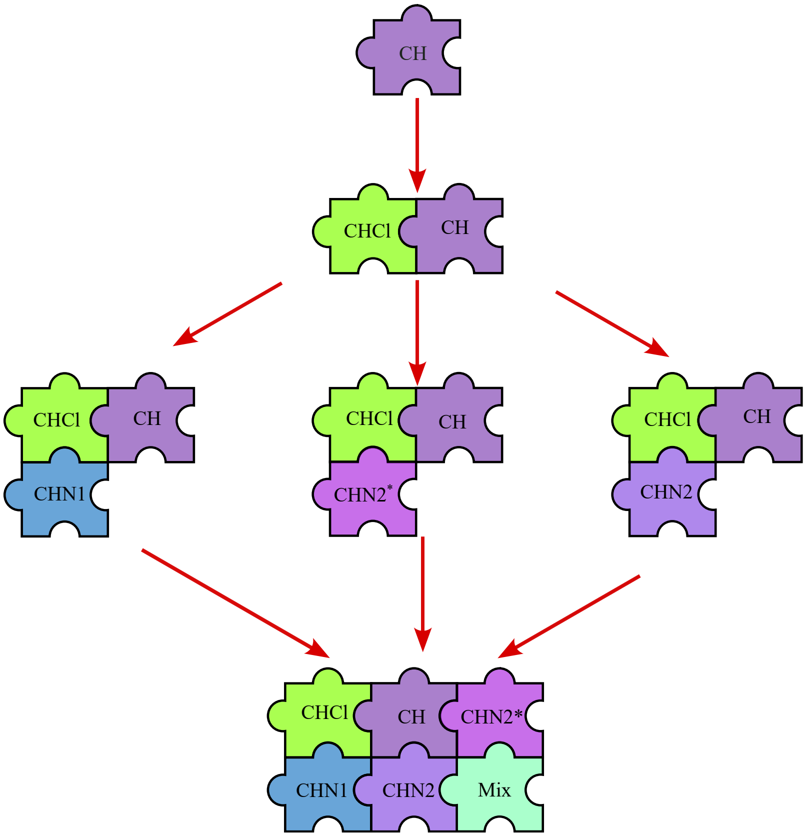

FIG. 2: Stratified informed training strategy: Progressive incorporation of molecular subsets (CH, CHCl, CHN1, CHN2, CHN3) through hierarchical training. Each subset undergoes independent optimization, contributing to the final parameter set. The hierarchical puzzle structure visually represents the merging process, reflecting the stratified weighting applied to ensure full dataset diversity.

C. Optimization Process

We obtain the final PPP parameters by minimizing a loss function

$$f = w_{D_1}(E_{D_1,\mathrm{calc}} - E_{D_1,\mathrm{exp}})^2 + w_{\mathrm{brt}}(E_{\mathrm{brt,calc}} - E_{\mathrm{brt,exp}})^2 + w_I(I^{\mathrm{rel}}_{D_1,\mathrm{calc}} - I^{\mathrm{rel}}_{D_1,\mathrm{exp}})^2, \tag{4}$$

where each squared-difference term represents the deviation between calculated and experimental values E_{D_1} (energy of first excited state), E_{brt} (energy of excited state that has the greatest oscillator strength with ground state), and $I_{D_1}^{\text{rel}}$ (ratio of the oscillator strength of the D_1 state to the brightest state). For hydrocarbons, the weights w_{D1}, w_{brt} were set to 1 eV^{-2} and w_I set to 0. For heterocycles where intensity data for D_1 and bright states could be found, w_{D1}, w_{brt} were set to 1 eV^{-2} and w_I set to 1, otherwise w_{D1}, w_{brt} were set to 1 eV^{-2} and w_I set to 0 (see data availability for the experimental UV-Visible data collected for each radical). Initial parameter guesses were taken from literature (see Table II), and optimized geometries (in `.xyz` format, see Section I and data Availability in the main text) were read into ExROPPP to compute electronic properties of interest. The deviation between computed and experimental values was then encoded in the loss function Equation 4. An iterative loop, updating parameters and re-evaluating f, continued until convergence criteria were met.

The Nelder-Mead and L-BFGS-B methods were used to iteratively minimize Equation 4 by updating the PPP parameters until convergence. Nelder–Mead was selected for most refinements due to its derivative-free nature and straightforward handling of parameter bounds, ensuring physically meaningful values within ExROPPP. Convergence was declared once both the parameter changes δx and the fitness change δf fell below 10^{-6}. In contrast, L-BFGS-B—a gradient-based method—was employed to optimize weighting factors in the "divide and conquer" approach, where the smooth dependence of the loss on those weights made gradient information particularly beneficial. For L-BFGS-B, the convergence criterion replaced the parameter tolerance with a gradient threshold $\|g\| < 10^{-6}$. We found that the lowest fitness of 10.0 was achieved using the stratified informed approach, which only reached the convergence criterion of $< 10^{-6}$ for both δf and δx when re-introducing TTM-DAID and TTM-DACz' after 500 iterations. This model, referred to as stratified informed - 500 in

Table III and simply the "trained model" throughout the main text, was therefore selected as the final trained model for which all results herein and in the main text are based upon, and its parameters are given in Table XII.

D. Generating Absorption Spectra for Target Variables

1. *Details of simulation of spectra*

To calculate the target variables E_{D1} (energy of the first excited state) and E_{brt} (energy of the brightest excited state), the absorption spectra were simulated by plotting the lowest 25 eigenvalues of the ExROPPP excited-state Hamiltonian with their oscillator strengths, broadened using a Lorentzian function. The oscillator strengths f_{0u} for each XCIS state $|\Psi_u\rangle$ (the eigenvectors of the XCIS Hamiltonian) are calculated from their transition dipole moments $\hat{\boldsymbol{\mu}}_{0u}$ using the formula[25]

$$f_{0u} = \frac{2}{3}\big(E_u - E_0\big)\left|\langle\Psi_0|\,\hat{\boldsymbol{\mu}}\,|\Psi_u\rangle\right|^2 \tag{5}$$

in atomic units. The transition dipole moments are calculated using the expression

$$\hat{\boldsymbol{\mu}}_{0u} = \langle\Psi_0|\,\hat{\boldsymbol{\mu}}\,|\Psi_u\rangle = \sum_I\sum_J S_{I,0}S_{J,u}\,\langle\Phi_I|\,\hat{\boldsymbol{\mu}}\,|\Phi_J\rangle \tag{6}$$

where $|\Phi_{I,J}\rangle$ are the XCIS basis states including the ground state

$$|\Phi_{I,J}\rangle \in \{\left|{}^2\Phi_0\right\rangle, \left|{}^2\Psi_{\bar{i}}^{\bar{0}}\right\rangle, \left|{}^2\Psi_0^{j'}\right\rangle, \left|{}^{2\mathrm{S}}\Psi_i^{j'}\right\rangle, \left|{}^{2\mathrm{T}}\Psi_i^{j'}\right\rangle, \left|{}^4\Psi_i^{j'}\right\rangle\} \tag{7}$$

and $S_{I,0}$ and $S_{J,u}$ are the CI expansion coefficients for the XCIS ground state and XCIS state $|\Psi_u\rangle$ respectively, given by

$$\begin{aligned}|\Psi_u\rangle =& S_{0,u}\left|{}^2\Phi_0\right\rangle + \sum_i S_{i0,u}\left|{}^2\Psi_{\bar{i}}^{\bar{0}}\right\rangle + \sum_{j'} S_{0j',u}\left|{}^2\Psi_0^{j'}\right\rangle \\ &+ \sum_{ij'}\big(S_{2\mathrm{S}ij,u}\left|{}^{2\mathrm{S}}\Psi_i^{j'}\right\rangle + S_{2\mathrm{T}ij,u}\left|{}^{2\mathrm{T}}\Psi_i^{j'}\right\rangle + S_{4ij,u}\left|{}^4\Psi_i^{j'}\right\rangle\big).\end{aligned} \tag{8}$$

3

A Lorentzian broadening function was applied to each eigenvalue, with the Full Width at Half Maximum (FWHM) determined based on a 20 nm splitting at a reference wavelength of 300 nm. This method effectively simulates the spectral line shapes by incorporating the natural linewidth of transitions, ensuring alignment with physical observations. By broadening the eigenvalues in energy units, the spectra reflect realistic absorption profiles, capturing both peak intensities and positions for accurate comparison to experimental data.

2. Extra absorptions in simulated spectra

In the ExROPPP calculations, the ground state was allowed to mix with excited configurations (as the form of ROHF we elected to use does not fully satisfy Brillouin's theorem)[3,26–28] and the transition dipole moments were adjusted for this ground state mixing according to Eq. (6). Including ground state mixing in the Hamiltonian was done as it should give the most accurate excited state energies and is the same method used in the original implementation of ExROPPP for hydrocarbon radicals (which gave accurate excitation energies).[3] However, it has been noted previously in the original implementation of XCIS that this ground state mixing is deliberately switched off to maintain the size-consistency of the method.[27] We found that removing the ground state mixing of excited configurations for the trained parameters after training gave cleaner looking spectra with minimal unwanted new absorptions, but with poorer accuracy for the excitation energies compared to the trained parameters *with* ground state mixing turned on (although this is not surprising since the method was not re-trained with ground state mixing turned off). These spectra are presented in Figures 3 and 4. All trained parameters were obtained *with* ground state mixing turned on. It is therefore suggested that a combination

of this ground state mixing and using a fitness function of selected peaks leads to these anomalous absorptions, and in future work one might try to retrain the model with ground state mixing disabled and using a fitness function based on the entire spectrum, such as optimal transport, where full spectra are available, to possibly improve spectra by minimising artificial absorptions.

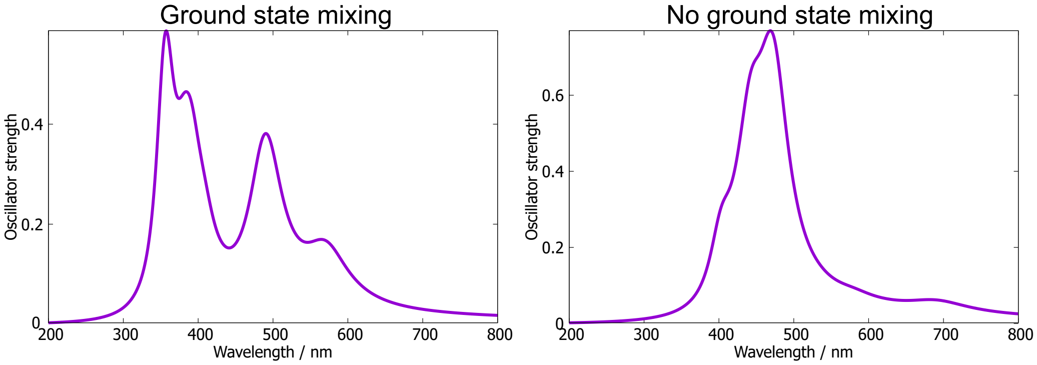

FIG. 3: Influence of ground state mixing on the UV-Visible absorption spectrum of TTM-1Cz simulated by ExROPPP. On the left is the spectrum simulated using the normal code presented in Fig. 5 in the main text. On the right is the spectrum simulated using ground state mixing turned off in the calculation of the Hamiltonian.

3. Contraction of spectra

The ExROPPP simulated spectra, particularly for TTM-1Cz and TTM-1Cz-An in Fig. 5 in the main text, appear to be slightly contracted relative to the experimental spectra. I.e., rather than both the simulated D_1 and bright states being equally red- or blue-shifted from their experimental wavelengths, the simulated D_1 states appear to be more blue-shifted than the simulated bright states relative to experiment, causing a contracted appearance of the simulated spectra. A possible explanation may be

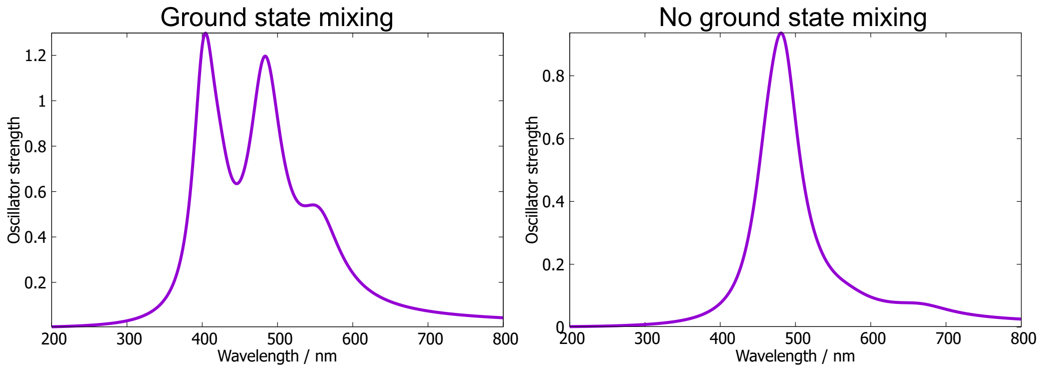

FIG. 4: Influence of ground state mixing on the UV-Visible absorption spectrum of TTM-1Cz-An simulated by ExROPPP. On the left is the spectrum simulated using the normal code presented in Fig. 5 in the main text. On the right is the spectrum simulated using ground state mixing turned off in the calculation of the Hamiltonian.

due to the local nature of PPP theory. Those spectra which appear to be contracted are of molecules which are composed of a chlorinated hydrocarbon radical (TTM) part bonded to a heterocyclic part. The D_1 (D_2 in TTM-1Cz-An) state is an excitation from the HOMO on the carbazole part to the SOMO on the radical part whose energy is strongly affected by the nitrogen parameters, whereas the bright absorption is mainly the local excitation on the radical part whose energy is mainly dependent on the carbon parameters.[7] Therefore, depending on the parameters (specifically the relative size of the ϵ_{C}, ϵ_{N} and U_{C}, U_{N} parameters) the spectra could be contracted or expanded relative to the experimental spectra.

TABLE III: Summary of training strategies.[a] [b]

Training strategy	ExROPPP states	Tolerance	Total fitness	RMSE / eV	SRCC
bruteforce	30	$f6x3$	12.94	0.2752	0.8405
bruteforce	30	$f6x6$	11.54	0.2620	0.8667
bruteforce	25	$f6x6$	12.54	0.2768	0.8386
bruteforce	25	$f6x3$	12.94	0.2763	0.8402
bruteforce	25	$f5x4$	13.04	0.2775	0.8211
bruteforce	25	$f5x3$	13.04	0.2775	0.8401
bruteforce	35	$f6x6$	15.938	0.3026	0.8270
stratified informed - 2500	25	$f6x3$	11.29	0.2555	0.8851
stratified informed - 2000	25	$f6x3$	10.32	0.2435	0.8835
stratified informed - 1500	25	$f6x3$	10.46	0.2447	0.8806
stratified informed - 1000	25	$f6x3$	10.15	0.2426	0.8839
stratified informed - 500	25	$f6x6$	10.00	0.2408	0.8832
stratified informed - 500	25	$f6x3$	11.34	0.2558	0.8857
divide and conquer, weighted	25	$f6x3$	12.55	0.2695	0.8398
divide and conquer, equal	25	$f6x3$	13.13	0.2759	0.8380
divide and conquer, LBFGSB	25	$f6g6$	14.99	0.2991	0.8419

[a] We abbreviate the convergence tolerances of $\delta f < 10^{-n}$, $\delta x < 10^{-n}$ and $\|g\| < 10^{-n}$ as fn, xn and gn for the total fitness change, parameter change and gradient respectively. E.g. $f6x3$ means the optimisation converges when $\delta f < 10^{-6}$ and $\delta x < 10^{-3}$.

[b] 'ExROPPP States' refers to the number of lowest energy eigenvalues of the ExROPPP Hamiltonian solved for.

III. VALIDATION

A. K-fold Validations

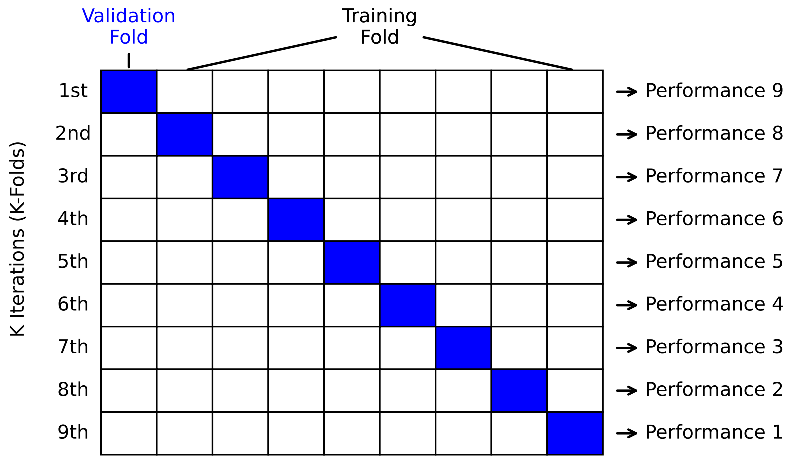

FIG. 5: Schematic depiction of 9-fold cross validation

In this study, we employed 9-fold cross-validation to evaluate the model's robustness against data noise. Specifically, the dataset was split into nine subsets (folds), and in each iteration one fold was withheld from training, while the remaining folds served as the training set. This process was repeated nine times, ensuring that every fold contributed to both training and validation. Finally, the optimized parameters obtained from each fold were tested on the entire dataset (including the previously withheld fold) to assess variability in the total fitness and thus gauge sensitivity to data noise.

Two methods were used to construct the 9 folds. The first was a manual assignment across six non-disjoint molecular groups (hydrocarbons, heterocycles, pyr-

role, aniline, pyridine, and pyridine–pyrrole mixtures), ensuring that each fold contained a representative distribution of all molecule types. The second method relied on `StratifiedKFold` from `scikit-learn`, where each molecule group was given a unique identifier. Stratified splitting then preserved the relative proportions of each group in every fold, using `random_state=42` and `random_state=11` for brute-force training and stratified informed training scheme respectively.

TABLE IV: Initial parameter set for stratified K-fold ML

Parameter	Value	Unit
ϵ_{C}	0	eV
ϵ_{N1}	-3.23	eV
ϵ_{N2}	-17.51	eV
ϵ_{Cl}	-10.47	eV
A_{CC}	-22.76	eV
A_{CN1}	-24.09	eV
A_{CN2}	-23.65	eV
A_{CCl}	-27.05	eV
b_{CC}	1.69	$\mathrm{\AA}^{-1}$
b_{CN1}	1.77	$\mathrm{\AA}^{-1}$
b_{CN2}	1.42	$\mathrm{\AA}^{-1}$
b_{CCl}	1.44	$\mathrm{\AA}^{-1}$
U_{C}	8.17	eV
U_{N1}	12.91	eV
U_{N2}	18.05	eV
U_{Cl}	9.55	eV
$r_{0,\mathrm{C}}$	1.17	Å
$r_{0,\mathrm{N1}}$	1.17	Å
$r_{0,\mathrm{N2}}$	1.14	Å
$r_{0,\mathrm{Cl}}$	2.21	Å

Regarding the initial parameters for the brute force and stratified informed approaches in the K-fold cross-validation, there was a slight difference. For the brute

force method, the starting point was the standard literature parameters in Table II. However, as previously discussed, the parameters from the penultimate learning layer triggered Fock-matrix convergence failures when TTM-DAID and TTM-DACz' were included, rendering them unsuitable for initialization. Instead, we employed the same parameter set, shown in Table IV, that had been used to launch the final training round—i.e., the one obtained after 500 iterations, prior to reintroducing the two problematic molecules. This choice ensured stable convergence and mitigated the issues encountered with the alternative starting point.

B. K-fold Results

TABLE V: 9-Fold Validations generated with arbitary stratified selection

Fold 1	Fold 2	Fold 3	Fold 4	Fold 5
Allyl	Benzyl	DPM	Trityl	DPXM
TTM	PTM	M1TTM	M2TTM	M3TTM
2,6-X3TTM	2,5-X3TTM	2,4-X3TTM	2,6-ipP3TTM	C-01
TTM-ID2	TTM-ID3	TTM-3PCz	TTM-3NCz	C-13
TTM-TCz	PTM-3PCz	PTM-PCz	TTETM-Cz	TTM-Cz-Cl2
TTM-Cz-Ph	PTM-TPA	TTM-DPA	TTM-PDMAC	PTM-3NCz
TTM-DBPA	PyPHBTM	metaPyBTM'-py	metaPyBTM'-Tpy	PyBTM
PyBTM-Ph2	TTM-deltaPyID	TTM-AID	PyBTM-MeS2	TTM-2PYM
TTM-gammaPyID	TTM-alphaPyID2	TTM-betaPyID	TTM-DAID	TTM-DACz
**Fold 6**	**Fold 7**	**Fold 8**	**Fold 9**	
PDXM	TXM	PAH-20	C-07	
P3TTM	4-T3TTM	3-T3TTM	2-T3TTM	
3,5-X3TTM	CzBTM	TTM-1Cz-An	TTM-1Cz-PhAn	
TTM-1Cz	TTM-Cz2	TTM-Cz-Et	TTM-dbCz	
TTM-Cz-Ph2	TTM-bCz'	TTM-ID	TTM-Cz3	
TTM-bCz	PTM-TPA-Cl	C-16	PTM-TPA-Me2	
TTM-DFA	triPyM	metaPyBTM	PyBTM-MeS	
bisPyTM	PyBTM'-TPA	metaPyBTM-TPA-Me2	TTM-alphaPyID	
TTM-DACz'	TTM-deltaPyID2	TTM-Bi2	TTM-Bi3	

TABLE VI: 9-Fold Validation generated with Random Seed 42

Fold 1	Fold 2	Fold 3	Fold 4	Fold 5
PAH-20	Benzyl	DPXM	PDXM	C-07
TTM	4-T3TTM	P3TTM	2,5-X3TTM	M1TTM
C-01	M3TTM	2,4-X3TTM	2,6-ipP3TTM	M2TTM
TTM-1Cz-PhAN	TTM-1Cz-An	TTM-Cz-Ph2	TTM-Cz-Et2	TTM-3PCz
TTM-Cz2	TTM-TCz	TTM-3NCz	PTM-3NCz	TTM-ID3
C-13	TTM-bCz	TTM-bCz’	TTM-DPA	PTM-TPA-Me2
PTM-TPA-Cl	C-16	triPyM	PyBTM-MeS	PyBTM-MeS2
metaPyBTM	metaPyBTM’-py	TTM-2PYM	metaPyBTM’-Tpy	TTM-gammaPyID
PyBTM’-TPA	TTM-Bi2	TTM-alphaPyID	TTM-Bi3	TTM-DACz

Fold 6	Fold 7	Fold 8	Fold 9	
DPM	TXM	Allyl	Trityl	
3-T3TTM	2,6-X3TTM	PTM	2-T3TTM	
3,5-X3TTM	TTM-1Cz	TTM-dbCz	TTM-Cz-Ph	
TTETM-Cz	PTM-3PCz	CzBTM	TTM-Cz-Cl2	
PTM-PCz	TTM-ID	TTM-Cz3	TTM-ID2	
TTM-PDMAC	PTM-DBPA	TTM-DFA	PTM-TPA	
PyBTM-Ph2	PyPHBTM	PyBTM	bisPyTM	
TTM-DAID	TTM-deltaPyID	TTM-DACz’	TTM-betaPyID	
TTM-deltaPyID2	TTM-AID	TTM-alphaPyID2	metaPyBTM-TPA-Me2	

TABLE VII: Results for the 9-fold brute-force validation with arbitrary stratified selection process.

	Testing error	RMSD	MAE	SRCC	R^2
T1	1.36457	0.28023	0.19579	0.83658	0.81754
T2	2.09195	0.28079	0.19512	0.82831	0.81680
T3	0.89577	0.27504	0.19158	0.84307	0.82423
T4	1.80606	0.27953	0.19048	0.85209	0.81845
T5	1.26328	0.28047	0.19950	0.83116	0.81722
T6	0.60115	0.27789	0.19353	0.83774	0.82056
T7	0.82878	0.27976	0.19354	0.83760	0.81814
T8	4.19323	0.27497	0.18494	0.83918	0.82432
T9	2.83541	0.29913	0.20179	0.85813	0.79209
Average	1.76447	0.28087	0.19403	0.84043	0.81659

TABLE VIII: Results for the 9-fold bruteforce validation with random seed.

	Testing error	RMSD	MAE	SRCC	R^2
T1	4.68198	0.29361	0.19790	0.84452	0.79969
T2	3.01976	0.29222	0.19365	0.84609	0.80158
T3	1.13858	0.29526	0.20052	0.84635	0.79744
T4	0.63061	0.29611	0.20213	0.84914	0.79627
T5	2.02445	0.32520	0.23610	0.86480	0.75426
T6	1.41429	0.30307	0.21031	0.84732	0.78658
T7	1.69246	0.29392	0.19792	0.84379	0.79927
T8	1.10362	0.29278	0.20007	0.84924	0.80082
T9	1.46367	0.29246	0.20351	0.85046	0.80125
Average	1.90771	0.29829	0.20468	0.84908	0.79302

TABLE IX: Results for the 9-fold stratified informed validation with arbitrary stratified selection process.

	Testing error	RMSD	MAE	SRCC	R^2
T1	0.98000	0.22485	0.15507	0.87153	0.88253
T2	1.25372	0.25089	0.16999	0.88013	0.85374
T3	1.30707	0.23643	0.16452	0.87152	0.87012
T4	0.89713	0.24283	0.16249	0.87847	0.86299
T5	1.69978	0.23358	0.16069	0.88307	0.87322
T6	0.48069	0.24415	0.16587	0.88197	0.86149
T7	0.92692	0.25489	0.17160	0.88230	0.84904
T8	2.70904	0.23820	0.16408	0.87472	0.86816
T9	1.74562	0.26047	0.17543	0.88595	0.84236
Average	1.33333	0.242921	0.165527	0.87885	0.86263

TABLE X: Results for the 9-fold stratified informed validation random seed.

	Testing error	RMSD	MAE	SRCC	R^2
T1	0.99035	0.27132	0.18136	0.88319	0.82895
T2	2.83746	0.25304	0.17069	0.88123	0.85122
T3	0.43605	0.27844	0.18568	0.89097	0.81985
T4	0.56714	0.27910	0.18900	0.89009	0.81900
T5	1.31757	0.26460	0.17666	0.89014	0.83732
T6	1.43360	0.27893	0.18406	0.88883	0.81922
T7	3.84767	0.26400	0.17406	0.89196	0.83805
T8	1.76147	0.24615	0.16244	0.88835	0.93104
T9	2.58413	0.29626	0.20550	0.85053	0.79601
Average	1.75283	0.27321	0.18834	0.88337	0.82620

TABLE XI: Summarising results for four ML sets

	Total testing errors	RMSD	MAE	SRCC	R^2
Brute-force selection	15.88021	0.28087	0.19403	0.84043	0.81659
Brute-force random	17.16942	0.29829	0.20468	0.84908	0.79302
Stratified selection	11.99997	0.242921	0.165527	0.87885	0.86263
Stratified random	15.77545	0.27321	0.18834	0.88337	0.82620
Average	15.20626	0.26567	0.18263	0.86293	0.83514

IV. RESULTS AND ANALYSIS

A. Optimized parameters

TABLE XII: Optimized PPP parameter set from the stratified informed - 500 model with convergence tolerance $f6x6$, selected as the final trained model (see Table III).

Parameter	Value	Unit
ϵ_{C}	0	eV
ϵ_{N1}	-3.49	eV
ϵ_{N2}	-17.78	eV
ϵ_{Cl}	-10.34	eV
A_{CC}	-22.72	eV
A_{CN1}	-25.23	eV
A_{CN2}	-24.74	eV
A_{CCl}	-26.02	eV
b_{CC}	1.71	Å^{-1}
b_{CN1}	1.77	Å^{-1}
b_{CN2}	1.43	Å^{-1}
b_{CCl}	1.45	Å^{-1}
U_{C}	8.42	eV
U_{N1}	12.81	eV
U_{N2}	17.98	eV
U_{Cl}	9.64	eV
$r_{0,\mathrm{C}}$	1.17	Å
$r_{0,\mathrm{N1}}$	1.20	Å

Table XII continued on next page ...

Table XII continued from previous page ...

Parameter	Value	Unit
$r_{0,\mathrm{N2}}$	1.11	Å
$r_{0,\mathrm{Cl}}$	2.25	Å

B. Analysis of the optimized parameters

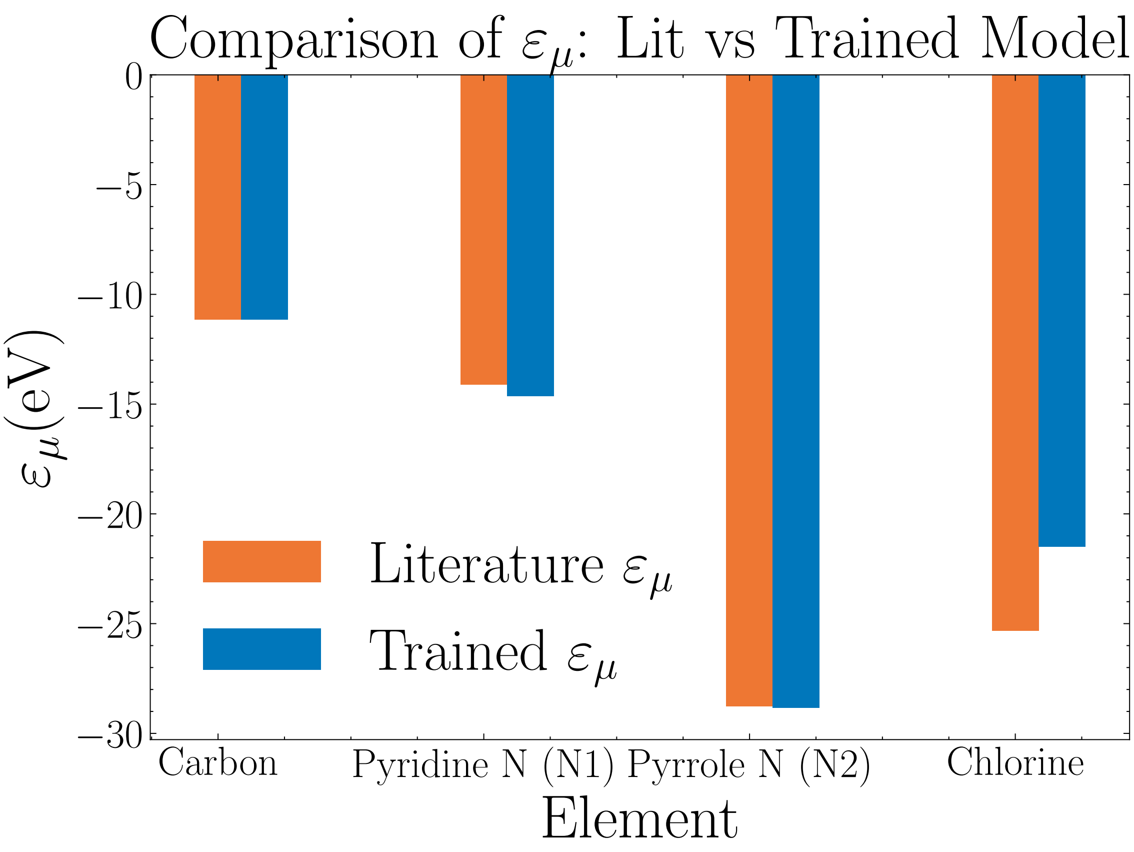

FIG. 6: Comparison of diagonal core Hamiltonian terms between literature and trained model for Carbon, Pyridine Nitrogen (N1), Pyrrole Nitrogen (N2), and Chlorine.

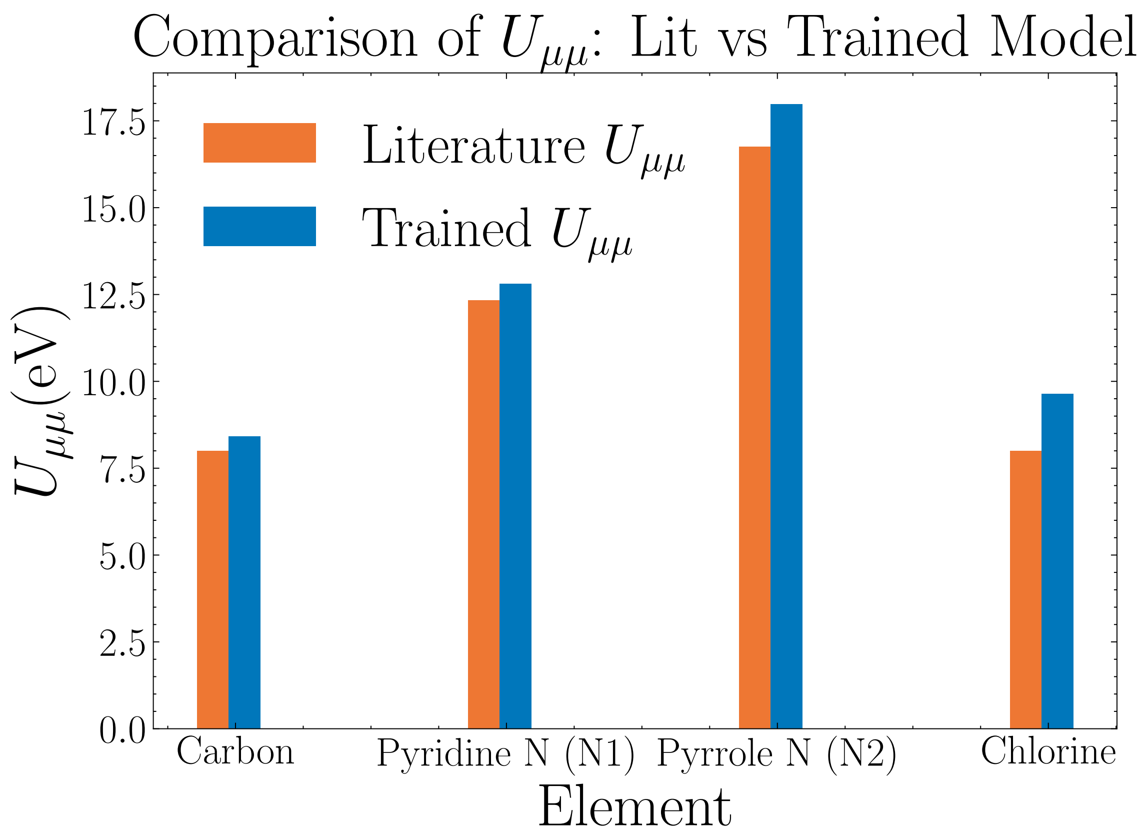

FIG. 7: Comparison of two-electron one-centre repulsion terms between literature and trained model for Carbon, Pyridine Nitrogen (N1), Pyrrole Nitrogen (N2), and Chlorine.

1. *Optimized ε Terms (Core Hamiltonian)*

Although some physical interpretability is sacrificed to achieve better agreement with experimental data, the trained ε parameters (diagonal core energies) largely retain a reasonable ordering consistent with their approximate relation to valence-state ionization potentials. For instance, the values for pyridine-type nitrogen (N1) are less negative than those for pyrrole-type nitrogen (N2), reflecting the higher effective

nuclear charge experienced in the latter (i.e., a "second" ionization scenario). Likewise, chlorine's ε remains more negative than carbon's, aligning with the stronger nuclear charge of chlorine and the correspondingly higher binding of its valence electrons. While the magnitudes differ somewhat from the literature, the overall trend is preserved, maintaining a plausible physical basis.

2. Optimized U Terms (On-Site Electron Repulsion)

The trained U terms (Hubbard parameters) are generally larger than the literature values but mostly preserve their relative ordering. Conceptually, U can be interpreted either as the intra-orbital electron–electron repulsion, which tends to be smaller for more diffuse orbitals, or as a measure of atomic "softness," incorporating both ionization potential and electron affinity. Nitrogen, with its more localized valence orbital, therefore shows the highest U, while carbon remains lower, and chlorine increases substantially—likely reflecting the model's response to chlorine's strong electron-withdrawing effect in conjugated systems.

3. Resonance Integrals $t_{\mu\nu}$

Certain fitted resonance integrals deviate from simple overlap-based expectations, most notably the unexpectedly large t_{CN2}. This appears to compensate for the strong influence of pyrrole-type nitrogen on low-energy transitions (e.g., the D_1 state), as enlarging t_{CN2} helps match experimental intensities in radicals with a donor-acceptor push-pull structure with chlorinated hydrocarbon radical (TTM/PTM) and pyrrole-nitrogen containing heterocyclic (e.g. carbazole) parts. This structural motif is present in around half of the training set. The relationship of the hopping integral ($t_{\mu\nu}$ between chlorinated hydrocarbon radical and heterocyclic groups) with the D_1

intensity in such donor-acceptor radicals has previously been shown by Hele, where an increase in $t_{\mu\nu}$ is shown to lead to a larger D_1 intensity.[7] Meanwhile, the C–Cl hopping integral t_{CCl}, though smaller than its C–C, still exceeds a strictly orbital-based estimate, underscoring how the model balances multiple transitions simultaneously. While such departures reduce straightforward chemical interpretations, the ε and U terms retain trends consistent with ionization potentials and orbital localization.

Additional evidence underscores the model's sensitivity to these resonance parameters. For example, decreasing the pre-exponential factor for the C–N2 resonance from -22.163 to -14.163 (thereby reducing $t_{C\text{–}N2}$ from $-3.275\,\mathrm{eV}$ to $-1.875\,\mathrm{eV}$ at $r_{\mu v} = 1.41\mathring{A}$) markedly alters the predicted spectra when all other parameters are held fixed. The D_1 state of TTM-1Cz blueshifts from $2.171\,\mathrm{eV}$ ($571.09\,\mathrm{nm}$) to $2.228\,\mathrm{eV}$ ($556.48\,\mathrm{nm}$), deviating further from the experimental $603\,\mathrm{nm}$, while the calculated dipole moment drops from 0.10947 to 0.02146. Moreover, the electronic transition's composition changes from including the HOMO–SOMO $\left|\Psi_{\bar{1}}^{\bar{0}}\right\rangle$ excitation to a $\left|\Psi_{\bar{2}}^{\bar{0}}\right\rangle$ excitation. These shifts illustrate how strongly a change in a single resonance integral can influence overall spectral properties, highlighting the complexity of simultaneously fitting multiple excited states and transitions - sometimes at the expense of certain aspects of physical interpretability.

C. Mathematical Expressions for Statistical Metrics

1. Root Mean Square Deviation (RMSD)

The RMSD quantifies the average magnitude of the deviation between calculated and experimental values:

$$\mathrm{RMSD} = \sqrt{\frac{1}{N}\sum_{i=1}^{N}\left(y_i^{\mathrm{calc}} - y_i^{\mathrm{exp}}\right)^2}$$

where:

- y_i^{calc}: Calculated value for the i-th data point,
- y_i^{exp}: Experimental value for the i-th data point,
- N: Total number of data points.

2. Mean Absolute Error (MAE)

The MAE provides the average absolute difference between calculated and experimental values:

$$\text{MAE} = \frac{1}{N} \sum_{i=1}^{N} \left| y_i^{\text{calc}} - y_i^{\text{exp}} \right|$$

where:

- y_i^{calc}: Calculated value for the i-th data point,
- y_i^{exp}: Experimental value for the i-th data point,
- N: Total number of data points.

3. Spearman Rank Correlation Coefficient (SRCC)

The Spearman Rank Correlation measures the monotonic relationship between experimental and calculated values:

$$\rho = 1 - \frac{6 \sum_{i=1}^{N} \left(R_i^{\text{calc}} - R_i^{\text{exp}} \right)^2}{N \left(N^2 - 1 \right)}$$

where:

- R_i^{calc}: Rank of the i-th calculated value,

- $R_i^{\exp}$: Rank of the i-th experimental value,
- N: Total number of data points.

4. Coefficient of Determination (R^2)

The R^2 value quantifies how well the calculated values explain the variability of the experimental values:

$$R^2 = 1 - \frac{\sum_{i=1}^{N} \left(y_i^{\exp} - y_i^{\mathrm{calc}}\right)^2}{\sum_{i=1}^{N} \left(y_i^{\exp} - \overline{y}^{\exp}\right)^2}$$

where:

- y_i^{calc}: Calculated value for the i-th data point,
- $y_i^{\exp}$: Experimental value for the i-th data point,
- $\overline{y}^{\exp}$: Mean of the experimental values,
- N: Total number of data points.

D. Results of the trained ExROPPP model

The global optimized parameters trained on all molecules (trained model) were compared to the parameters obtained from the literature using an exponentially decaying hopping term (see Table II) which were used as the initial guess for training, referred to as the 'literature parameters' here and throughout the main text . Additionally, to more closely compare the trained model (using an exponentially decaying function for the hopping t terms) to the original implementation of ExROPPP (which used a step-function for the hopping t terms with the MN value of $t = -2.4$ eV for carbon double/aromatic bonds), the results using an adjusted exponential pre-factor A_{CC} for carbon are also presented. A_{CC} was set to -24.47749 eV such that the carbon aromatic/double bond hopping term $t_{\mathrm{C,double}}$ equals the Mataga-Nishimoto value of -2.4 at a bond length of 1.4 Å which is typical of CC double/aromatic bonds in conjugated molecules, while keeping the distance scaling parameter b_{CC} the same as before.[21,29,30]. These results are presented in Table XIII and Fig. 8.

The trained parameters are clearly a significant improvement over both the literature parameters in Table II and the literature parameters with $A_{\mathrm{CC}} = -24.47749$ eV with significantly lower RMSE, MAE and fitness, and R^2 and SRCC much closer to the ideal value of 1. Inclusion of this adjusted set of literature parameters shows that, while they are more accurate than the unaltered literature parameters from Table II, these parameters are still far from optimal with almost double the target of 0.3 eV for RMSE and MAE and R^2 of 0.23 which (although at least positive) is still poor. We accept that there may be other PPP parametrizations existing in the literature which we are unaware of at the time which may perform better than the literature parameters presented here. However, the aim of this work is not to compare many different literature parameterizations and evaluate all of them, but instead to show how a far more optimal PPP parameter set for the electronically

excited states of radicals than is typically available in the literature can be learned by training on empirical data.

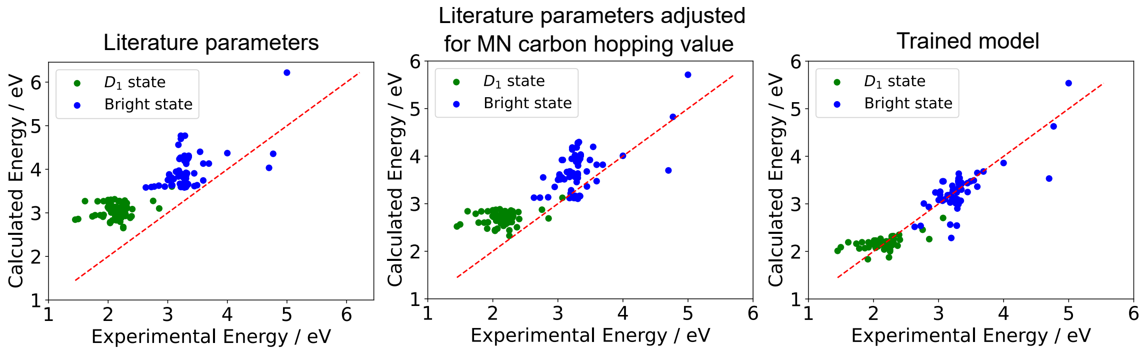

FIG. 8: UV/Visible absorption energies of the 81-radical training set stimulated by ExROPPP and compared to experimental values, using three different sets of parameters — left: literature parameters (Table II), center: literature parameters with $A_{\mathrm{CC}} = -24.47749$ eV fitted to the Mataga-Nishimoto hopping parameter,[21] and right: optimised parameters (Table XII).

TABLE XIII: Root mean-squared errors (RMSE), mean absolute errors (MAE), R^2 and Spearman's rank correlation coefficients (SRCC) for the trained ExROPPP model compared to ExROPPP with two sets of parameters obtained from the literature (see above), calculated for all states in the training set of 81 organic radicals.

	Literature parameters	Literature parameters with $A_{\mathrm{CC}} = -24.47749$ eV	Trained Model	Target
Total Fitness	117.44	53.30	10.00	
RMSE (all states) / eV	0.86	0.57	0.24	< 0.3
MAE (all states) / eV	0.80	0.51	0.16	< 0.3
R^2 (all states)	-0.71	0.23	0.87	close to 1
SRCC (all states)	0.79	0.81	0.88	close to 1

TABLE XIV: Root mean-squared errors (RMSE), mean absolute errors (MAE), R^2 and Spearman's rank correlation coefficients (SRCC) for the trained ExROPPP model with all 81 radicals compared to ExROPPP with 79 radicals excluding two abnormal radicals, TTM-1Cz-An and TTM-1Cz-PhAn.

	Including All Molecules	Only Normal Molecules
Total Fitness	10.00	9.72
RMSE (all states) / eV	0.24	0.24
MAE (all states) / eV	0.16	0.16
R^2 (all states)	0.87	0.87
SRCC (all states)	0.88	0.89

E. ExROPPP Simulated Molecular Orbitals

Here we compare a selection of orbitals computed using ExROPPP and ROHF.

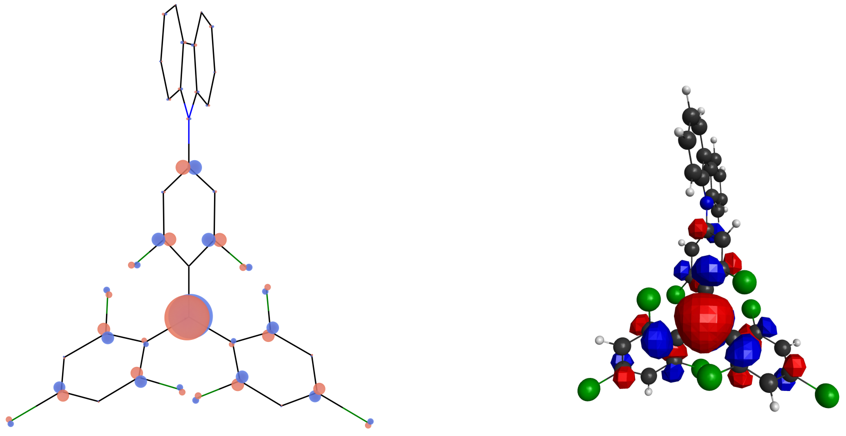

(a) ExROPPP (b) ROHF

FIG. 9: TTM-1Cz SOMO

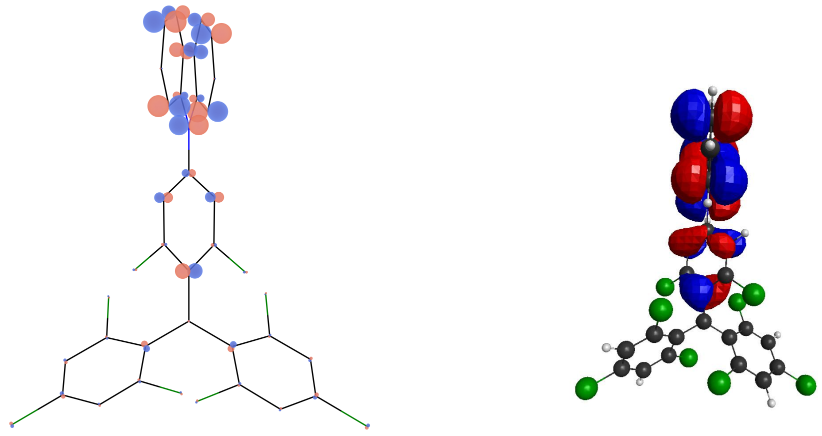

(a) ExROPPP (b) ROHF

FIG. 10: TTM-1Cz HOMO

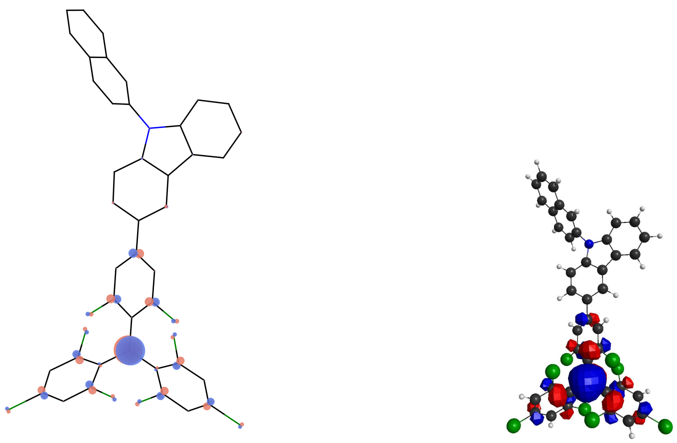

(a) ExROPPP (b) ROHF

FIG. 11: TTM-3NCz SOMO

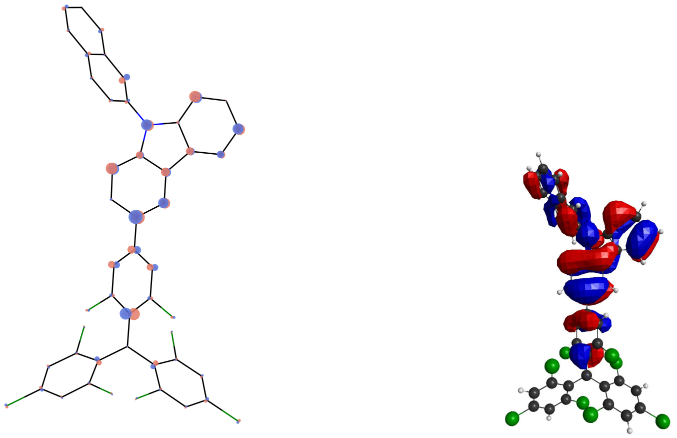

(a) ExROPPP (b) ROHF

FIG. 12: TTM-3NCz HOMO

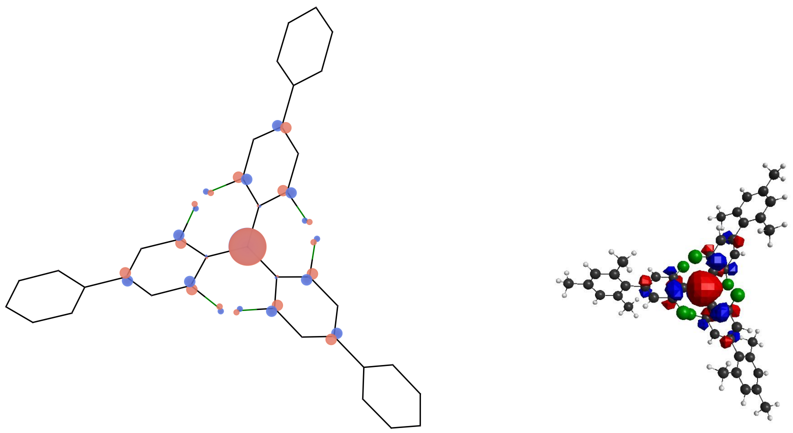

(a) ExROPPP (b) ROHF

FIG. 13: M_3TTM SOMO

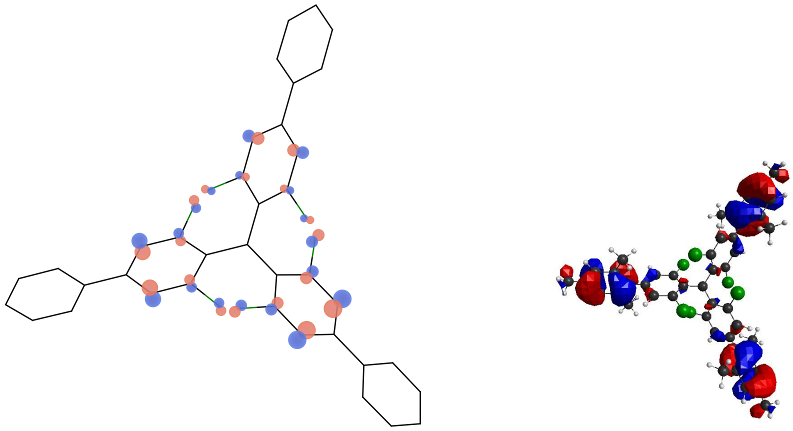

(a) ExROPPP (b) ROHF

FIG. 14: M3TTM HOMO

A closer examination of ExROPPP (using the trained parameters) and Restricted Open-Shell DFT (B3LYP/6-31G(d,p)) orbitals for TTM-based radicals

(TTM-1Cz, TTM-3NCz, and M_3TTM) reveals that the singly occupied molecular orbital (SOMO) is predominantly localized on the central triarylmethyl (TTM) carbon, consistent with the expected radical site. In TTM-1Cz and TTM-3NCz, additional electron density extends onto the adjacent phenyl rings, adopting a nodal pattern reflective of the π-conjugated framework.

For the HOMOs in TTM-1Cz and TTM-3NCz, density starts near the phenyl ring that bridges to the carbazole substituent and then extends further onto the carbazole moiety, displaying the correct nodal arrangement in the connecting region. This orbital structure preserves both local and global symmetry elements: for instance, the SOMO and HOMO of TTM-1Cz belong to the B irrep of the C_2 point group, while in M_3TTM they follow the A irrep of the C_3 point group. ExROPPP successfully reproduces these symmetry-driven distributions.

Although the visual representations of M_3TTM orbitals from ExROPPP appear to lack amplitude on certain peripheral substituents, inspection of the orbital coefficients shows small but nonzero contributions on the order of $\approx$0.007 relatively minor compared to the central values of ≈ 0.7. This difference may stem from a reduced degree of $\sigma - \pi$ separation or the pruned sp^3 carbons in M_3TTM. Nevertheless, the overall radical character and nodal structure remain well-captured.

V. EXROPPP AND PPP PARAMETERISATION

A. PPP Parametrization Scheme

The full realization of PPP method requires specific parameter values for its full implementation. These include the core Hamiltonian elements $\varepsilon\mu$, the two electron repulsion integral $\gamma_{\mu\nu}$, and the resonance integral $t_{\mu\nu}$.

B. One-electron Integrals

Inspired by Goeppert-Mayer and Skla, the Pariser-Parr form is:

$$\varepsilon_\mu = -\mathrm{IP}_\mu \tag{9}$$

where IP_μ is the valence-state ionization potential of the atom of interest. These were taken from literature by Beveridge and Hinze.[20]

Complexity arises in the treatment of resonance integral. Originally, $t_{\mu\nu}$ are handled semi-empirically, with various forms suggested. Pople proposed that $t_{\mu\nu}$ is proportional to the overlap integral,[31] typically involving Slater-type atomic orbitals (AOs) belonging to atoms μ and ν. This relationship can be expressed as follows:

$$t_{\mu\nu} = A_{\mu\nu} S_{\mu\nu} \tag{10}$$

This assumption is physically valid because the resonance integral measures the potential lowering of energy levels when an electron is in the electrostatic field of two atoms, thereby increasing bonding capacity with greater overlap.

The constant A depends solely on the nature of the bonding pairs. To approximate the overlap integral within the Slater-type orbital framework, we employed an exponential form as a first-order approximation, following Pariser and Pople:[32,33]:

$$t_{\mu\nu} = A_{\mu\nu} \exp(-b_{\mu\nu} r_{\mu\nu}) \tag{11}$$

Here, $b_{\mu\nu}$ is the combined Slater exponent, and $r_{\mu\nu}$ is the distance between atoms μ and ν. A further modification involves incorporating a dihedral angle term, scaling the hopping term by $\cos\theta$, where θ is the dihedral angle between two bonds. Usually $\theta \simeq 0$ for conjugated molecules, however, for situations where there are two staggered groups connected by a single bond in e.g. TTM, θ is often non-zero (i.e. the molecule is non-planar) and in which case the dihedral angle term is important to account for the geometry of the molecule.

The aforementioned form of the resonance integral is just one among several approximations that have been proposed. Ensuring compatibility between the exponential scheme of the resonance integral and the Mataga-Nishimoto scheme for two-electron integral parameterization presents a significant challenge. The final form of these parameters is often guided by chemical intuition, particularly the notion that bonding capacity is a continuous and gradually varying function of distance, albeit a simplified perspective.

Identifying a suitable starting point for literature parameters is also challenging. As noted, the parameter A depends on the nature of the bonding. For systems involving carbon, chlorine, pyrrole, and pyridine-type nitrogen, this necessitates technically four different types of exponents (describing C-C, C-N1, C-N2, C-Cl) and pre-exponential factors, making it a non-trivial task. To maintain consistency and comparability, the exponent and pre-exponential for C-C type bonds utilize values from Ref. 22 (see Table II). Unfortunately, no parameterization was provided for C-Cl, C-N1, or C-N2 bonds. Therefore, we kept the exponent fixed and varied the pre-exponential to align with the literature parameters suggested in Mataga et al[21] and Van-Catledge[23].

C. Two-electron Repulsion Integrals

The two-electron integrals depend on the internuclear distances and the nature of atoms μ and ν. Physically, $\gamma_{\mu\nu}$ represents the average electrostatic repulsion between electrons in valence atomic orbitals on atoms μ and ν. Conversely, $\gamma_{\nu\nu}$ describes the repulsion between two electrons on the same atom/orbital, akin to a Hubbard-like term. These integrals written explicitly:

$$\gamma_{\mu\nu} = \iint \phi_\mu^*(1)\phi_\mu(1)\frac{1}{|r_\mu - r_\nu|}\phi_\nu^*(2)\phi_\nu(2)d\tau_1 d\tau_2 \tag{12}$$

Though analytical solutions for Eq. 12 using Slater-type orbitals is plausible, involving complex coordinate transformation, it is non-trivial. Henceforth simplifying this, we consider:

$$\iint \Omega_\mu(1)\frac{1}{|r_\mu - r_\nu|}\Omega_\nu(2)d\tau_1 d\tau_2 \tag{13}$$

which represents classical electrostatic repulsions between charge distributions Ω_μ and Ω_ν. The Mataga-Nishimoto scheme extends this idea by approximating charge distribution interactions as point charges, simplifying the integral to::

$$\gamma_{\mu\nu} = \frac{U_{\mu\nu}}{1 + \frac{r_{\mu\nu}}{r_{0,\mu\nu}}} \tag{14}$$

where

$$U_{\mu\nu} = \frac{1}{2}(U_\mu + U_\nu) \tag{15}$$

$$r_{0,\mu\nu} = \frac{1}{2}(r_{0,\mu} + r_{0,\nu}) \tag{16}$$

Here, U is the Hubbard repulsion of atom μ and $r_{0,\mu}$ is a distance scaling constant. As the separation r approaches zero, Eq. (16) converges to U which is the one-center two-electron integral, arising from the localization of two electrons in the same orbital also known as the Hubbard potential.

VI. EXPERIMENTAL SECTION

A. Materials and synthesis

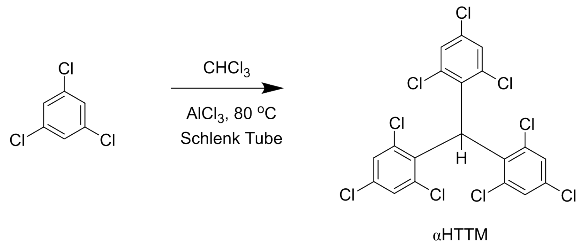

Tris(2,4,6-trichlorophenyl)methane (*α*HTTM). 1,3,5-trichlorobenzene (100 g, 551 mmol, 9 equiv.) was added into a round-bottom pressure flask and purged with Ar gas. To this, anhydrous $AlCl_3$ (8.981 g, 67.4 mmol, 1.1 equiv.) was added followed by further purging with Ar gas for 30 mins. Subsequently, anhydrous $CHCl_3$ (4.9 mL, 61.2 mmol, 1 equiv.) was added before heating the reaction mixture at 80 °C for 3 h. Once cooled to RT, the flask was opened to release the formed HCl gas (warning: highly pressurised gas) and then quenched with ice water (1 × 100 mL). The crude reaction mixture was dissolved using alternating additions of DCM and water before being extracted with water (3 × 200 mL) and dried using $MgSO_4$. The organic phase was concentrated *in vacuo*. The crude product was purified by column chromatography over silica gel (100% hexane), followed by recrystallisation from hexane/THF. The product was obtained as a white solid (15.21 g, 45%). ^{1}H NMR (400 MHz, $CDCl_3$) δ 7.36 (d, J = 2.3 Hz, 3H), 7.24 (d, J = 2.3 Hz, 3H), 6.68 (s, 1H). ^{13}C NMR (101 MHz, $CDCl_3$) δ 138.1, 137.2, 134.0, 134.0, 130.2, 128.6, 50.0. TOF-MS-ASAP$^-$ Calcd. for $[C_{19}H_6Cl_9]^-$: 548.7666. Found: m/z = 548.7674.

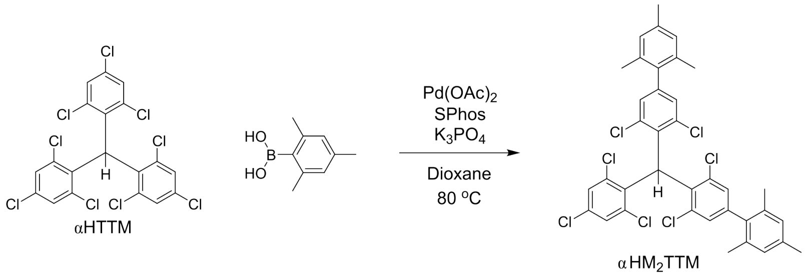

4',4'''-((2,4,6-trichlorophenyl)methylene)bis(3',5'-dichloro-2,4,6-trimethyl-1,1'-biphenyl) (*α*HM$_2$TTM). *α*HTTM (13.02 g, 23.5 mmol, 1 equiv.), mesitylboronic acid (11.554 g, 70.4 mmol, 3 equiv.), Pd(OAc)$_2$ (0.1054 g, 0.470 mmol, 0.02 equiv.), SPhos (0.3856 g, 0.939 mmol, 0.04 equiv.) and K_3PO_4 (23.93 g, 112 mmol, 4.8 equiv.) were added into a microwave vial and the vial was subjected to three vacuum/Ar gas refill cycles. Subsequently, anhydrous 1,4-dioxane (140 mL) was added and the mixture was purged with Ar gas for 15 minutes before being heated at 70 °C for 16 h. After cooling to RT, the mixture was diluted with hexane and extracted with water (3 × 200 mL). The organic phase was dried with $MgSO_4$ and concentrated *in vacuo*. The crude product was purified with column chromatography over silica gel (100% hexane). The product was obtained as a white solid (x g, x%). ^{1}H NMR (400 MHz, $CDCl_3$) δ 7.40 (d, J = 2.1 Hz, 1H), 7.27 (d, J = 2.3 Hz, 1H), 7.16 (dd, J = 6.2, 1.8 Hz, 2H), 7.07 – 7.00 (m, 2H), 6.94 (s, 4H), 6.91 (s, 1H), 2.32 (s, 5H), 2.04 (t, J = 4.0 Hz, 10H). ^{13}C NMR (101 MHz, $CDCl_3$) δ 142.7, 142.7, 138.6, 137.9, 137.9, 137.8, 137.8, 137.3, 137.1, 136.4, 136.4, 136.1, 136.1, 135.5, 134.1, 134.0, 133.8, 131.6, 131.5, 130.5, 129.8, 128.9, 128.7, 128.6, 50.8, 32.1, 23.1, 21.5, 21.0, 21.0, 21.00, 14.6. TOF-MS-ASAP$^-$ Calcd. for $[C_{37}H_{30}Cl_7]^-$: 717.0011. Found: m/z = 717.0038.

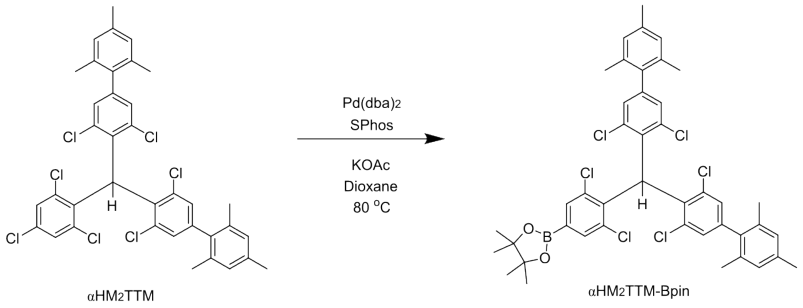

2-(4-(Bis(3,5-dichloro-2',4',6'-trimethyl-[1,1'-biphenyl]-4-yl)methyl)-3,5-dichlorophenyl)-4,4,5,5-tetramethyl-1,3,2-dioxaborolane ($\boldsymbol{\alpha}$HM$_2$TTM-Bpin). αHM$_2$TTM (1.417 g, 1.96 mmol, 1 equiv.), bis(pinacolato)diboron (0.7478 g, 2.94 mmol, 1.5 equiv.), Pd(dba)$_2$ (0.0226 g, 0.039 mmol, 0.02 equiv.), SPhos (0.0403 g, 0.098 mmol, 0.05 equiv.) and KOAc (0.3468 g, 3.53 mmol, 1.8 equiv.) were added into a microwave vial and subjected to three vacuum/ Ar gas refill cycles. Subsequently, anhydrous 1,4-dioxane (21 mL) was added and the mixture was purged with Ar gas for 15 minutes before being heated at 80 °C for 24 h. After cooling to RT, the mixture was diluted with hexane and extracted with water (3 × 100 mL). The organic phase was dried with $MgSO_4$ and concentrated *in vacuo*. The crude product was purified with column chromatography over silica gel (100% hexane). Before loading the crude product, the column was pre-treated using a mixture of 3% (v/v) acetic acid in hexane until the silica was saturated with acid. Residual acetic acid was washed out by passing hexane through the column. Following the removal of solvent, the solids were sonicated in MeOH until the resulting solution appeared colourless. The solvent was removed by centrifuging. The product was obtained as a white solid (0.9499 g, 56%). ^{1}H NMR (400 MHz, $CDCl_3$) δ 7.77 (d, J = 1.2 Hz, 1H), 7.64 (d, J = 1.2 Hz, 1H), 7.14 (dd, J = 11.6, 1.8 Hz, 2H), 7.01 (dd, J = 7.9, 1.8 Hz, 2H), 6.97 (s, 1H), 6.93 (s, 4H), 2.32 (s, 6H), 2.05 (m, 12H), 1.34 (d, J = 5.2 Hz, 12H). ^{13}C NMR (101

MHz, $CDCl_3$) δ 142.1, 139.1, 137.7, 137.7, 137.6, 137.5, 137.0, 136.9, 136.2, 136.2, 135.9, 135.8, 135.8, 134.4, 134.2, 134.1, 131.2, 131.1, 129.4, 129.3, 128.3, 84.6, 51.0, 25.2, 24.9, 21.2, 20.7, 20.7, 20.7. TOF-MS-ASAP$^+$ Calcd. for $[C_{43}H_{43}Cl_6O_2B]^+$: 811.1409. Found: m/z = 811.1413.

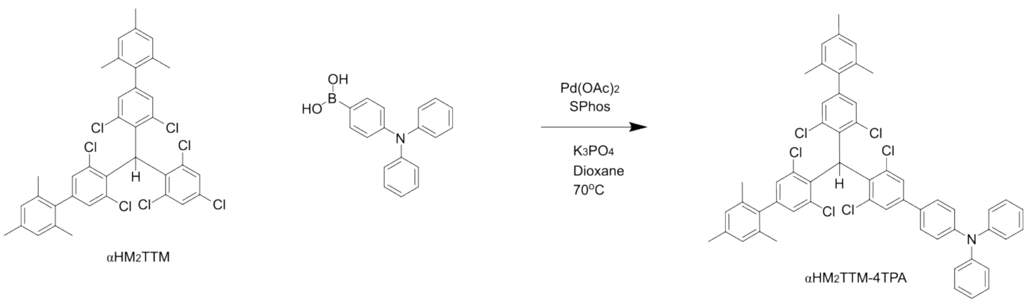

4'-(bis(3,5-dichloro-2',4',6'-trimethyl-[1,1'-biphenyl]-4-yl)methyl)-3',5'-dichloro-N,N-diphenyl-[1,1'-biphenyl]-4-amine (α$\mathbf{HM_2}$TTM-4TPA). **α**HM_2TTM (0.4000 g, 0.554 mmol, 1 equiv.), *p*TPA-$B(OH)_2$ (0.4006 g, 1.385 mmol, 2.5 equiv.), $Pd(OAc)_2$ (0.0025 g, 0.011 mmol, 0.02 equiv.), SPhos (0.0114 g, 0.028 mmol, 0.05 equiv.) and K_3PO_4 (0.2117 g, 0.998 mmol, 1.8 equiv.) were added into a microwave vial and the vial was subjected to three vacuum/Ar gas refill cycles. Subsequently, anhydrous 1,4-dioxane (10 mL) was added and the mixture was purged with Ar gas for 15 minutes before being heated at 70 °C for 16 h. After cooling to RT, the mixture was diluted with hexane and extracted with water (3 × 100 mL). The organic phase was dried with $MgSO_4$ and concentrated *in vacuo*. The crude product was purified with column chromatography over silica gel, by increasing the eluent polarity from hexane to 8% (v/v) DCM in hexane. The product was obtained as a white solid (0.4356 g, 84%). ^{1}H NMR (400 MHz, CD_2Cl_2) δ 7.62 (d, J = 2.0 Hz, 1H), 7.52 – 7.46 (m, 3H), 7.29 (m, J = 8.7, 7.5 Hz, 4H), 7.18 (dd, J = 4.1, 1.8 Hz, 2H), 7.14 – 7.03 (m, 10H), 6.98 (s, 1H), 6.93 (s, 4H), 2.30 (s, 6H), 2.04 (d, J = 2.9 Hz, 12H). ^{13}C NMR (101 MHz, CD_2Cl_2) δ 148.7, 147.8, 142.6, 142.6, 141.6, 138.2, 137.9, 137.8, 137.5, 137.1, 136.4, 136.0, 135.9,

134.5, 134.5, 134.4, 131.6, 131.5, 131.4, 129.8, 129.7, 128.5, 128.5, 128.2, 127.9, 127.6, 126.5, 125.3, 124.7, 124.4, 123.8, 123.5, 123.3, 51.0, 21.2, 20.7, 20.7, 20.7. TOF-MS-ES$^+$ Calcd. for $[C_{55}H_{44}N_1Cl_6]^+$: 927.1527. Found: m/z = 927.1512.

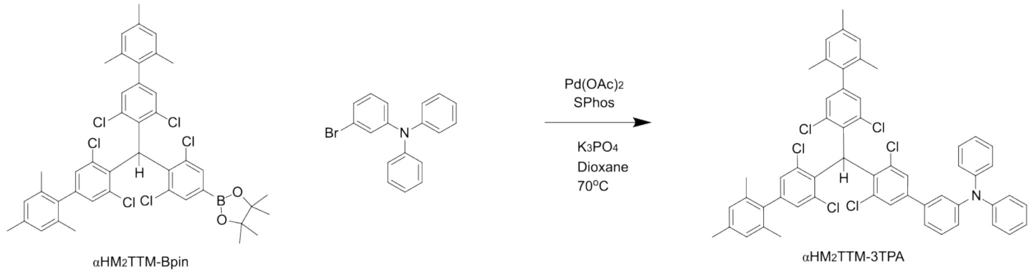

4'-(bis(3,5-dichloro-2',4',6'-trimethyl-[1,1'-biphenyl]-4-yl)methyl)-3',5'-dichloro-N,N-diphenyl-[1,1'-biphenyl]-3-amine (αHM_2TTM-3TPA). M_2TTM-Bpin (0.2000 g, 0.246 mmol, 1 equiv.), Br-*m*TPA (0.1993 g, 0.615 mmol, 2.5 equiv.), $Pd(OAc)_2$ (0.0011 g, 0.005 mmol, 0.02 equiv.), SPhos (0.0050 g, 0.012 mmol, 0.05 equiv.) and K_3PO_4 (0.0940 g, 0.443 mmol, 1.8 equiv.) were added into a microwave vial and the vial was subjected to three vacuum/Ar gas refill cycles. Subsequently, anhydrous 1,4-dioxane (5 mL) was added and the mixture was purged with Ar gas for 15 minutes before being heated at 70 °C for 16 h. After cooling to RT, the mixture was diluted with hexane and extracted with water (3 × 100 mL). The organic phase was dried with $MgSO_4$ and concentrated *in vacuo*. The crude product was purified with column chromatography over silica gel, by increasing the eluent polarity from hexane to 8% (v/v) DCM in hexane. The product was obtained as a white solid (0.1112 g, 47%). ^{1}H NMR (400 MHz, CD_2Cl_2) δ 7.54 (d, J = 2.1 Hz, 1H), 7.40 (d, J = 2.0 Hz, 1H), 7.37 – 7.21 (m, 7H), 7.17 (dd, J = 7.1, 1.8 Hz, 2H), 7.14 – 7.01 (m, 9H), 6.96 (s, 1H), 6.94 (s, 4H), 2.31 (s, 6H), 2.04 (d, J = 3.7 Hz, 12H). ^{13}C NMR (101 MHz, CD_2Cl_2) δ 149.1, 148.1, 142.7, 142.6, 141.9, 139.4, 138.1, 137.8, 137.8, 137.5, 137.1, 137.1, 136.4, 136.0, 135.9, 135.9, 135.9, 135.3, 134.4, 134.3, 131.6, 131.5, 130.3, 129.8, 129.7, 128.9,

128.5, 127.1, 124.7, 124.3, 123.5, 122.5, 121.5, 51.0, 21.2, 20.7, 20.7, 20.6. TOF-MS-ASAP$^+$ Calcd. for $[C_{55}H_{44}N_1Cl_6]^+$: 928.1605. Found: m/z = 928.1592.

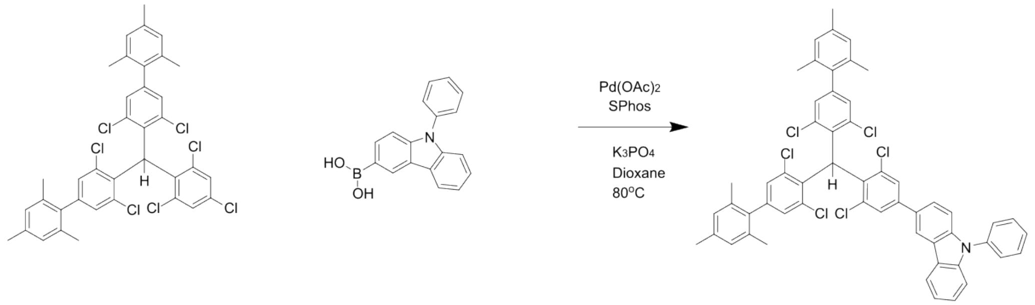

3-(4-(bis(3,5-dichloro-2',4',6'-trimethyl-[1,1'-biphenyl]-4-yl)methyl)-3,5-dichlorophenyl)-9-phenyl-9H-carbazole (αHM$_2$TTM-3PCz). αHM$_2$TTM (0.1770 g, 0.25 mmol, 1 equiv.), (9-phenyl-9H-carbazol-3-yl)boronic acid (0.1056 g, 0.37 mmol, 1.5 equiv.), $Pd(OAc)_2$ (0.0011 g, 0.0049 mmol, 0.02 equiv.), SPhos (0.0040 g, 0.0098 mmol, 0.04 equiv.) and K_3PO_4 (0.0937 g, 0.44 mmol, 1.8 equiv.) were added into a microwave vial and the vial was subjected to three vacuum/Ar gas refill cycles. Subsequently, anhydrous 1,4-dioxane (5 mL) was added and the mixture was purged with Ar gas for 15 minutes before being heated at 80 °C for 16 h. After cooling to RT, the mixture was diluted with hexane and extracted with water (3 × 200 mL). The organic phase was dried with $MgSO_4$ and concentrated *in vacuo.* The crude product was purified with column chromatography over silica gel by increasing the polarity from hexane to 10% (v/v) DCM in hexane. The product was obtained as a white solid (0.1713 g, 75%). ^{1}H NMR (400 MHz, CD_2Cl_2) δ 8.42 (d, J = 1.8 Hz, 1H), 8.20 (d, J = 7.8 Hz, 1H), 7.81 (d, J = 2.0 Hz, 1H), 7.71 – 7.63 (m, 4H), 7.62 – 7.58 (m, 2H), 7.54 – 7.47 (m, 2H), 7.47 – 7.41 (m, 2H), 7.33 (m, 1H), 7.20 (dd, J = 2.7, 1.7 Hz, 2H), 7.07 (dd, J = 3.3, 1.8 Hz, 2H), 7.03 (s, 1H), 6.94 (s, 4H), 2.31 (s, 6H), 2.06 (d, 12H). ^{13}C NMR (101 MHz, CD_2Cl_2) δ 142.9, 142.6, 142.6, 141.9, 141.3, 138.2, 137.9, 137.9, 137.8, 137.8, 137.8, 137.6, 137.2, 136.4, 136.0, 136.0, 135.9, 134.6, 134.5, 134.2, 131.6, 131.6, 130.4, 130.2, 129.9,

129.8, 128.9, 128.5, 128.1, 127.4, 127.1, 126.9, 125.3, 124.4, 123.7, 120.8, 120.7, 119.1, 110.7, 110.4, 51.0, 32.0, 23.1, 21.2, 20.7, 20.7, 14.3. TOF-MS-ES$^+$ Calcd. for $[C_{55}H_{41}Cl_6N]^+$: 925.1370. Found: m/z = 925.1362.

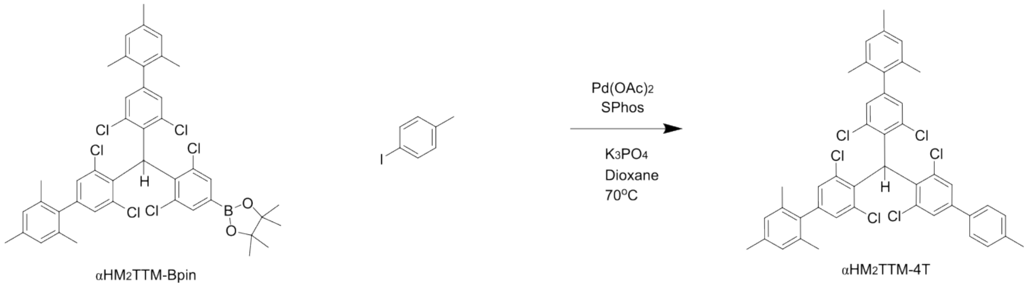

4',4'''-((3,5-dichloro-4'-methyl-[1,1'-biphenyl]-4-yl)methylene)bis(3',5'-dichloro-2,4,6-trimethyl- 1,1'-biphenyl) (αHM$_2$TTM-4T).

αHM$_2$TTM-Bpin (0.2 g, 0.246 mmol, 1 equiv.), 4-iodotoluene (0.1340 g, 0.615 mmol, 2.5 equiv.), $Pd(OAc)_2$ (0.0011 g, 0.005 mmol, 0.02 equiv.), SPhos (0.0050 g, 0.012 mmol, 0.05 equiv.) and K_3PO_4 (0.0940 g, 0.443 mmol, 1.8 equiv.) were added into a microwave vial and subjected to three vacuum/ Ar gas refill cycles. Subsequently, anhydrous 1,4-dioxane (5 mL) was added and the mixture was purged with Ar gas for 15 minutes before being heated at 80 °C for 24 h. After cooling to RT, the mixture was diluted with hexane and extracted with water (3 × 100 mL). The organic phase was dried with $MgSO_4$ and concentrated *in vacuo.* The crude product was purified with column chromatography over silica gel, by increasing the eluent polarity from hexane to 10% (v/v) DCM in hexane. The product was obtained as a white solid (0.1185 g, 62%). ^{1}H NMR (400 MHz, $CDCl_3$) δ 7.60 (d, J = 2.0 Hz, 1H), 7.48 (m, 3H), 7.25 (d, J = 8.1 Hz, 2H), 7.17 (dd, J = 4.6, 1.8 Hz, 2H), 7.03 (dd, J = 3.9, 1.8 Hz, 2H), 6.99 (s, 1H), 6.94 (s, 4H), 2.40 (s, 3H), 2.33 (s, 6H), 2.06 (d, J = 4.3 Hz, 12H). ^{13}C NMR (101 MHz, $CDCl_3$) δ 142.1, 141.6, 138.5, 137.9, 137.7, 137.7, 137.5, 137.3, 137.0, 137.0, 136.2, 135.9, 135.9, 135.8, 135.1, 134.5, 134.3, 134.2, 131.2, 131.1, 129.8, 129.4, 129.4, 128.5,

128.3, 128.3, 126.8, 126.7, 50.7, 21.3, 21.2, 20.7, 20.7, 20.7. TOF-MS-ASAP$^+$ Calcd. for $[C_{44}H_{37}Cl_6]^+$: 775.1026. Found: m/z = 775.1005.

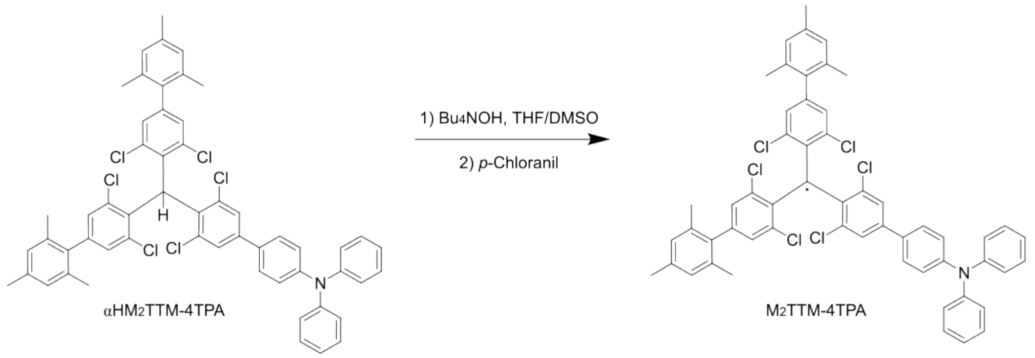

4'-(bis(3,5-dichloro-2',4',6'-trimethyl-[1,1'-biphenyl]-4-yl)methyl)-3',5'-dichloro-N,N-diphenyl-[1,1'-biphenyl]-4-amine Radical (M_2TTM-4TPA). **α**HM_2TTM-4TPA (0.4356 g, 0.468 mmol, 1 equiv.) was added into a one-neck round-bottom flask and subjected to three vacuum/ Ar gas refill cycles. Subsequently, anhydrous DMSO (60 mL) and THF (20 mL) were added and the mixture was purged with Ar gas for 10 minutes before the addition of 40% Bu_4NOH (aq) (1.25 mL, 1.927 mmol, 4 equiv.). The reaction mixture was heated at 35 °C for 16 hours whilst shielded from light. *p*-Chloranil (0.2877 g, 1.170 mmol, 2.5 equiv.) was added and the system left to react for a further 30 minutes. The mixture was diluted with hexane and extracted with water (3 × 100 mL). The organic phase was dried with $MgSO_4$ and concentrated *in vacuo*. The material was purified by prep-HPLC (eluent: DCM/ *n*-hexane 1:9 v/v, flow rate: 20 mL/min) through multiple 2.5 mL injections of a 2 mM solution of the crude product. Finally, the solvent was removed and the solids dried under high vacuum. The product was obtained as a green solid (0.0562 g, 12.5%). TOF-MS-ES$^+$ Calcd. for $[C_{55}H_{42}N_1Cl_6]^+$: 926.1448. Found: m/z = 926.1485.

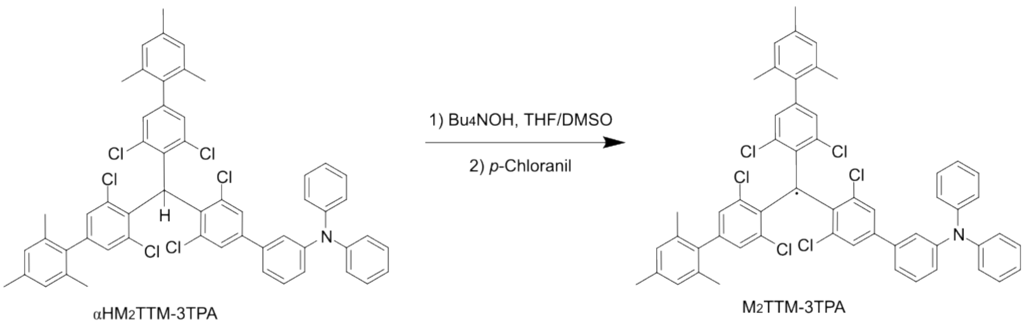

4'-(bis(3,5-dichloro-2',4',6'-trimethyl-[1,1'-biphenyl]-4-yl)methyl)-3',5'-dichloro-N,N-diphenyl-[1,1'-biphenyl]-3-amine Radical (M_2TTM-3TPA). **α**HM_2TTM-3TPA (0.0985 g, 0.103 mmol, 1 equiv.) was added into a microwave vial and subjected to three vacuum/ Ar gas refill cycles. Subsequently, anhydrous DMSO (15 mL) and THF (5 mL) were added and the mixture was purged with Ar gas for 10 minutes before the addition of 40% Bu_4NOH (aq) (0.27 mL, 0.416 mmol, 4 equiv.). The reaction mixture was heated at 35 °C for 16 hours whilst shielded from light. *p*-Chloranil (0.0632 g, 0.2569 mmol, 2.5 equiv.) was added and the system left to react for a further 30 minutes. The mixture was diluted with hexane and extracted with water (3 × 100 mL). The organic phase was dried with $MgSO_4$ and concentrated *in vacuo*. The crude product was purified with column chromatography over silica gel, by increasing the eluent polarity from hexane to x% (v/v) DCM in hexane. Finally, the solvent was removed and the solids dried under high vacuum. The product was obtained as a red solid (0.0614 g, 42%). TOF-MS-ES$^+$ Calcd. for $[C_{55}H_{43}N_1Cl_6]^+$: 927.1527. Found: m/z = 927.1492.

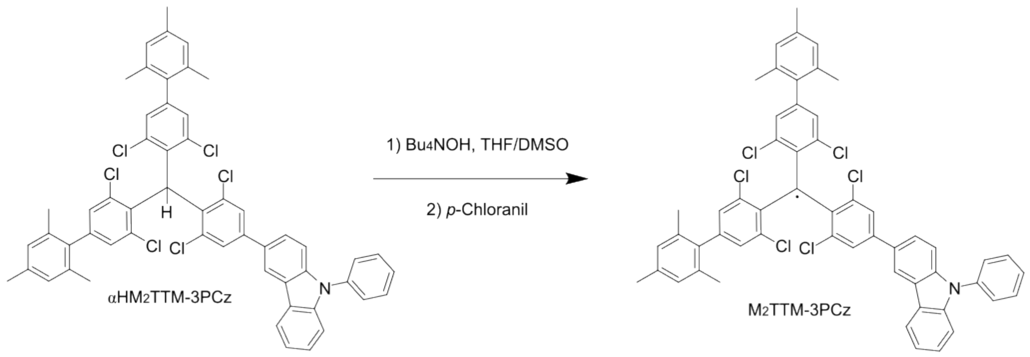

3-(4-(bis(3,5-dichloro-2',4',6'-trimethyl-[1,1'-biphenyl]-4-yl)methyl)-3,5-dichlorophenyl)-9-phenyl-9H-carbazole Radical (M_2TTM-3PCz).

αHM_2TTM-3PCz (0.1563 g, 0.168 mmol, 1 equiv.) was added into a microwave vial and subjected to three vacuum/ Ar gas refill cycles. Subsequently, anhydrous DMSO (24 mL) and THF (8 mL) were added and the mixture was purged with Ar gas for 10 minutes before the addition of 40% Bu_4NOH (aq) (0.44 mL, 0.673 mmol, 4 equiv.). The reaction mixture was heated at 35 °C for 16 hours whilst shielded from light. *p*-Chloranil (0.1035g, 0.421 mmol, 2.5 equiv.) was added and the system left to react for a further 30 minutes. The mixture was diluted with hexane and extracted with water (3 × 100 mL). The organic phase was dried with $MgSO_4$ and concentrated *in vacuo*. The crude product was purified with column chromatography over silica gel, by increasing the eluent polarity from hexane to 10% (v/v) DCM in hexane. Finally, the solvent was removed and the solids dried under high vacuum. The product was obtained as a green solid (0.1342 g, 86%). TOF-MS-ES$^+$ Calcd. for $[C_{55}H_{40}Cl_6N]^+$: 924.1292. Found: m/z = 924.2688.

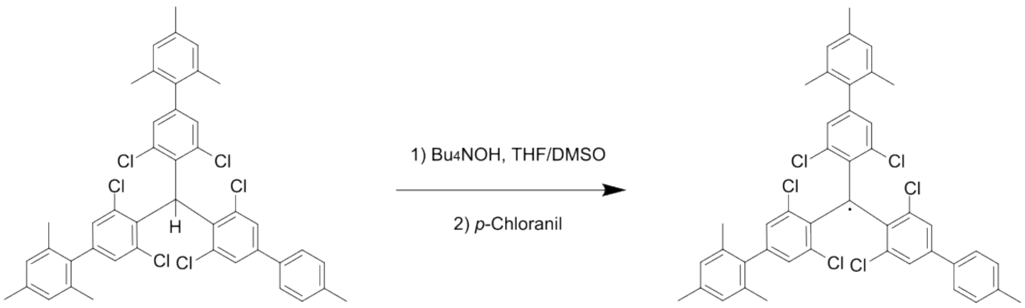

αHM2TTM-4T M2TTM-4T

αHM_2TTM-4T (0.1185 g, 0.143 mmol, 1 equiv.) was added into a microwave vial and subjected to three vacuum/ Ar gas refill cycles. Subsequently, anhydrous DMSO (15 mL) and THF (5 mL) were added and the mixture was purged with Ar gas for 10 minutes before the addition of 40% Bu_4NOH (aq) (0.4 mL, 0.617 mmol, 4.3 equiv.). The reaction mixture was heated at 35 °C for 16 hours whilst shielded from light. *p*-Chloranil (0.0876 g, 0.356 mmol, 2.5 equiv.) was added and the system left to react for a further 30 minutes. The mixture was diluted with hexane and extracted with water (3 × 100 mL). The organic phase was dried with $MgSO_4$ and concentrated *in vacuo*. The crude product was purified with column chromatography over silica gel, by increasing the eluent polarity from hexane to 6% (v/v) DCM in hexane. Finally, the solvent was removed and the solids dried under high vacuum. The product was obtained as a red solid (0.0438 g, 37%).

TOF-MS-ASAP$^-$ Calcd. for $[C_{44}H_{38}Cl_6]^-$: 773.0870. Found: m/z = 773.0871.

B. NMR Spectra

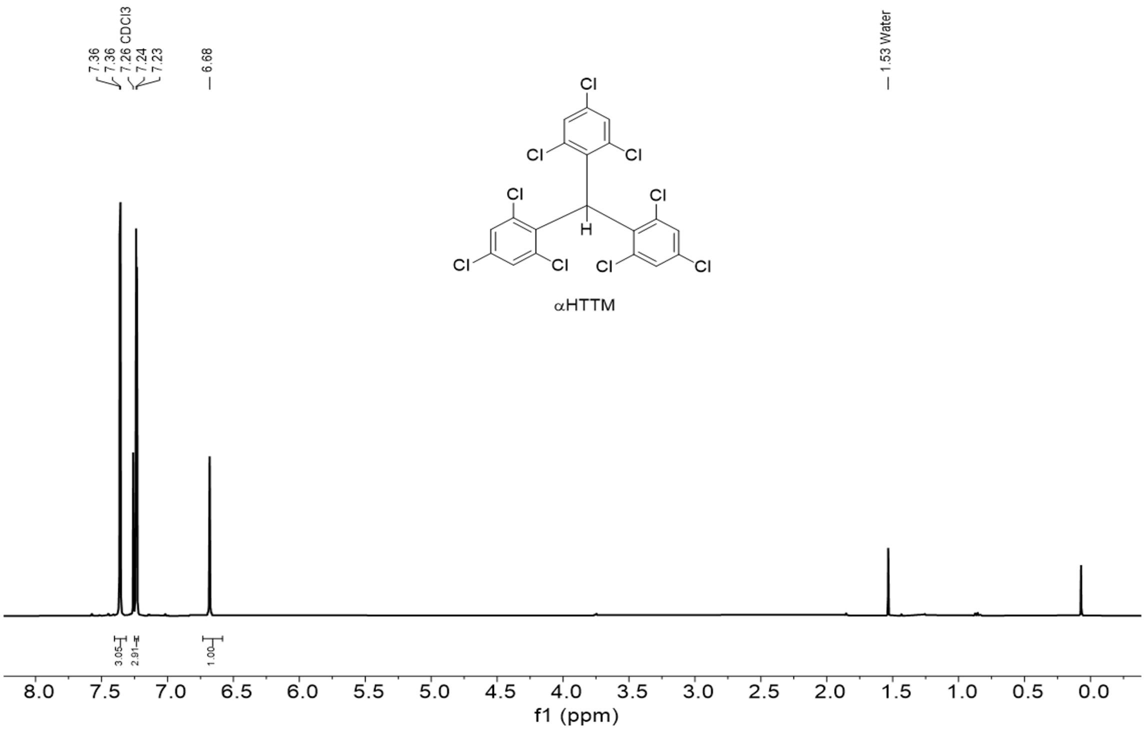

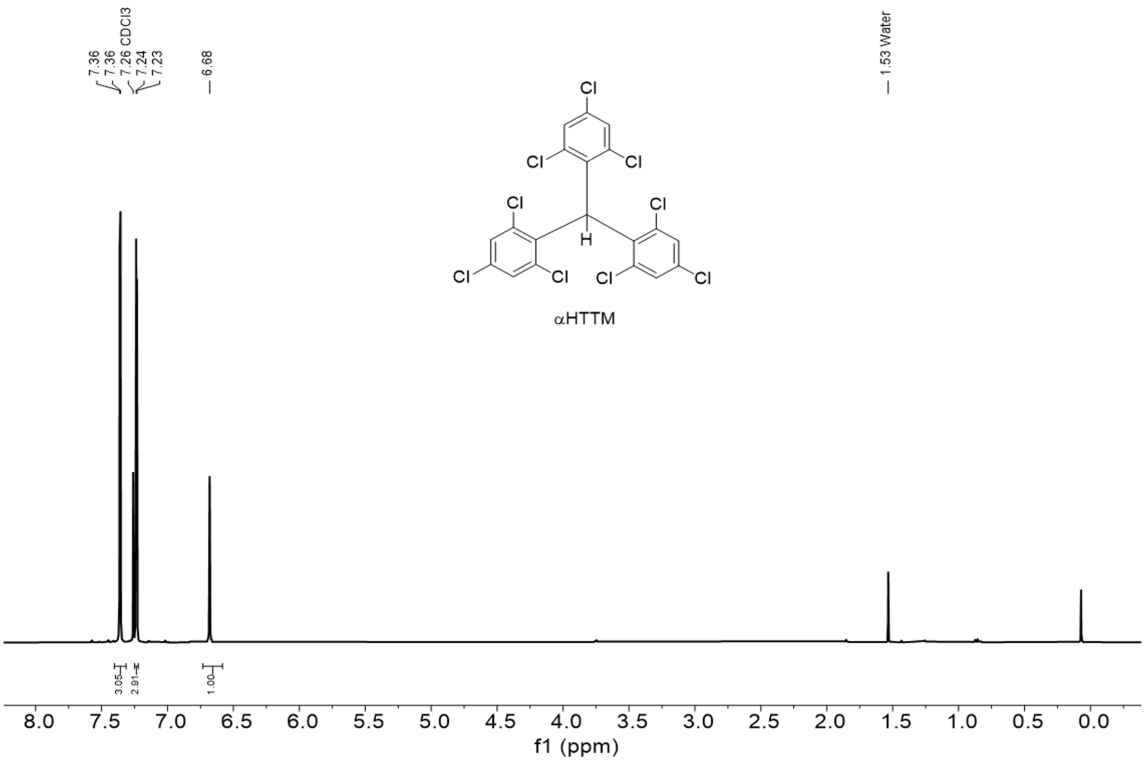
7.36
7.36
7.26 CDCl3
7.24
7.23
6.68
1.53 Water
Cl
H
αHTTM
3.05
2.91
1.00
8.0 7.5 7.0 6.5 6.0 5.5 5.0 4.5 4.0 3.5 3.0 2.5 2.0 1.5 1.0 0.5 0.0
f1 (ppm)

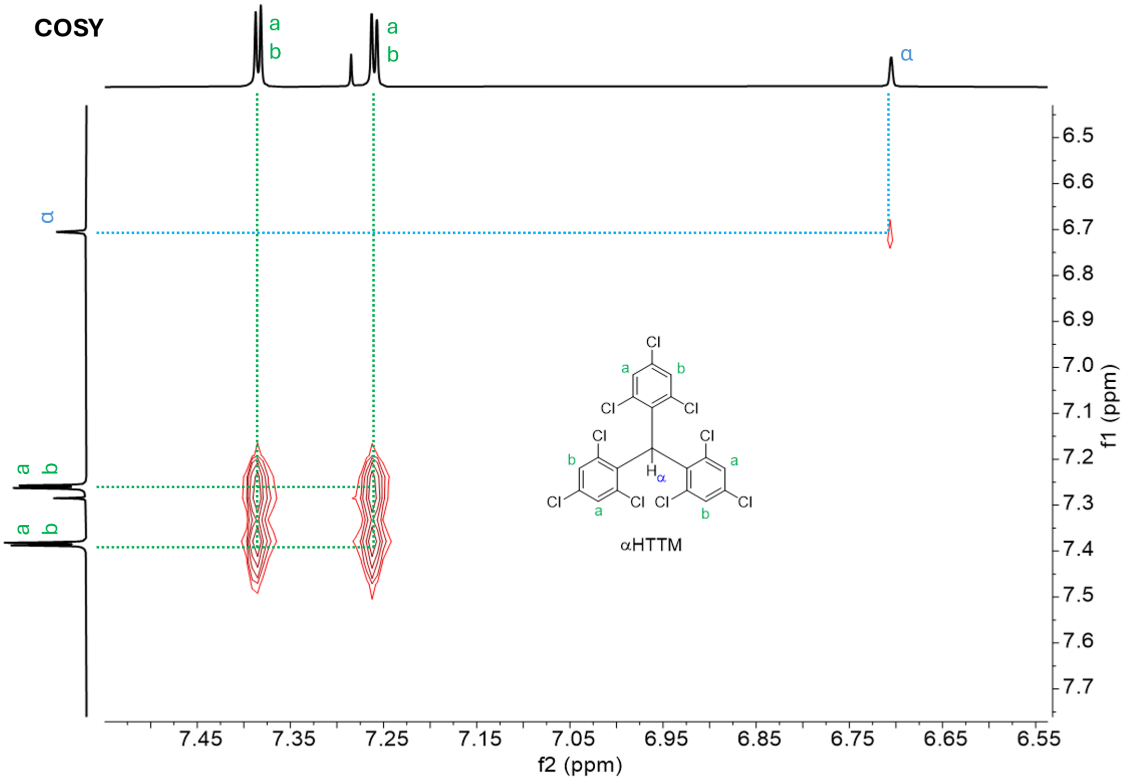
COSY
a
b
α
6.5
6.6
6.7
6.8
6.9
7.0
7.1
7.2
7.3
7.4
7.5
7.6
7.7
f1 (ppm)
7.45
7.35
7.25
7.15
7.05
6.95
6.85
6.75
6.65
6.55
f2 (ppm)
Cl
Hα
αHTTM

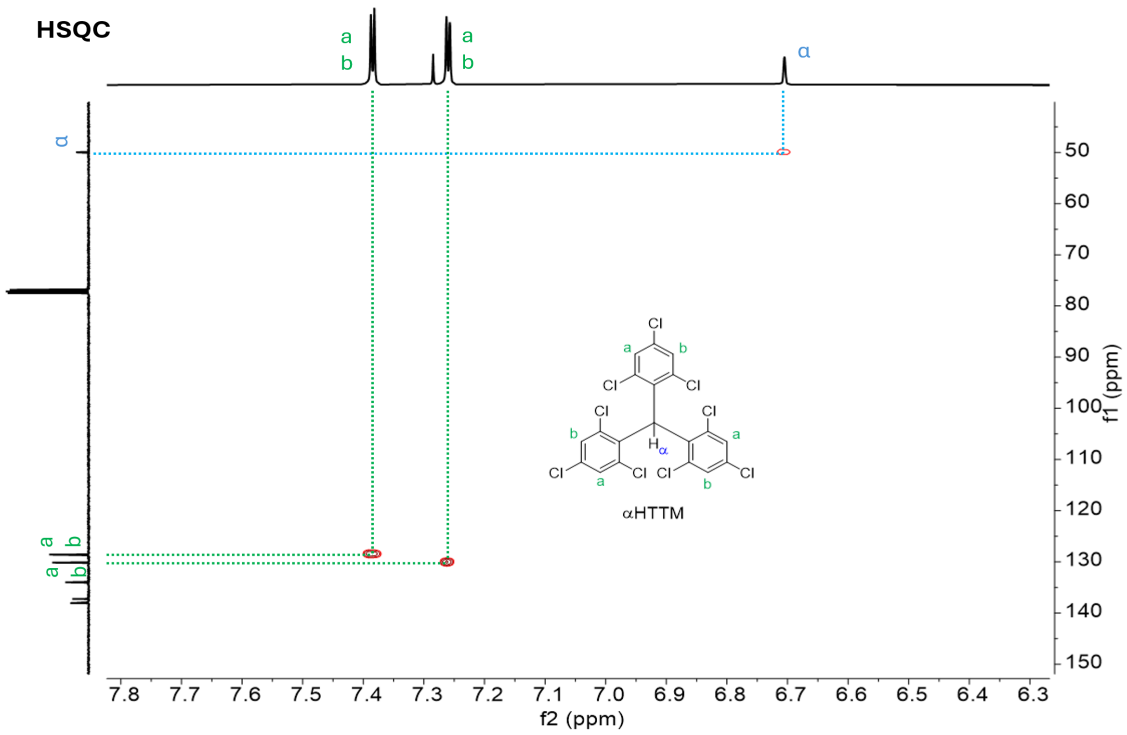
HSQC
a
b
a
b
α
α
a
b
a
b
Cl
a
b
Cl
Cl
Cl
Cl
b
a
Hα
Cl
Cl
Cl
Cl
a
b
αHTTM
7.8
7.7
7.6
7.5
7.4
7.3
7.2
7.1
7.0
6.9
6.8
6.7
6.6
6.5
6.4
6.3
f2 (ppm)
50
60
70
80
90
100
110
120
130
140
150
f1 (ppm)

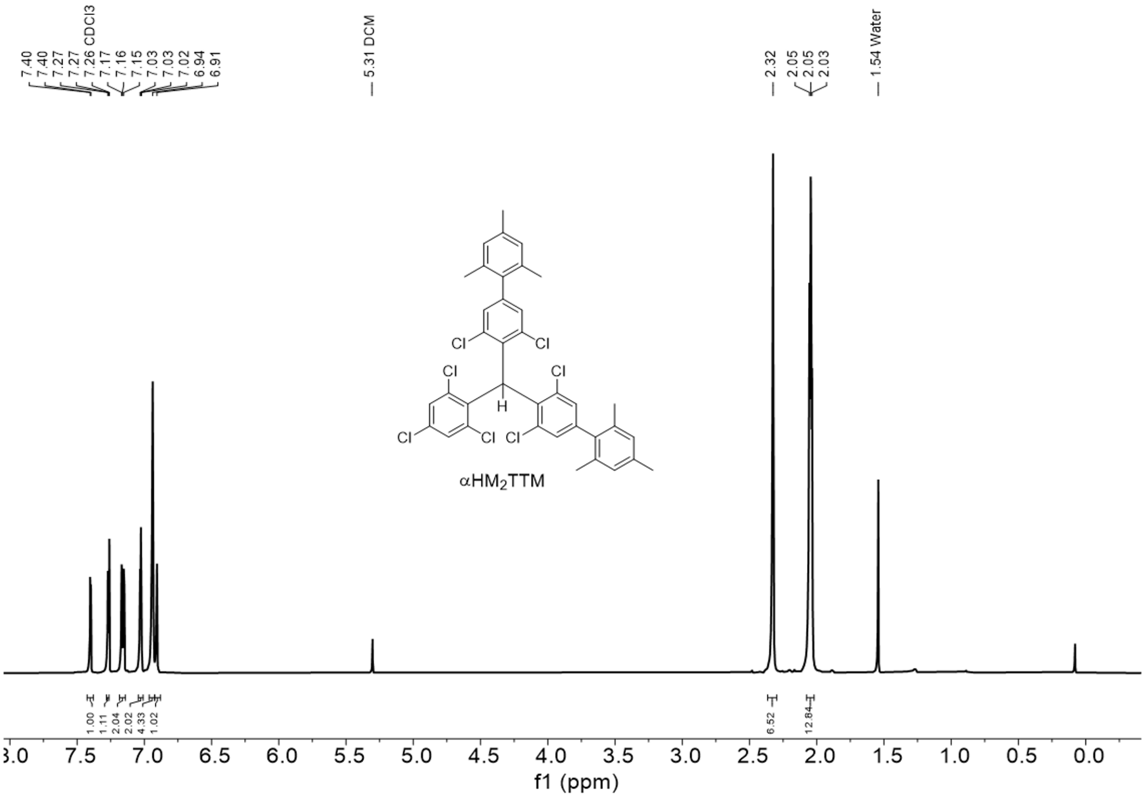
7.40
7.40
7.27
7.27
7.26 CDCl3
7.17
7.16
7.15
7.03
7.03
7.02
6.94
6.91
5.31 DCM
2.32
2.05
2.05
2.03
1.54 Water
Cl
Cl
Cl
Cl
H
Cl
Cl
Cl
Cl
αHM2TTM
1.00
1.11
2.04
2.02
4.33
1.02
6.52
12.84
8.0
7.5
7.0
6.5
6.0
5.5
5.0
4.5
4.0
3.5
3.0
2.5
2.0
1.5
1.0
0.5
0.0
f1 (ppm)

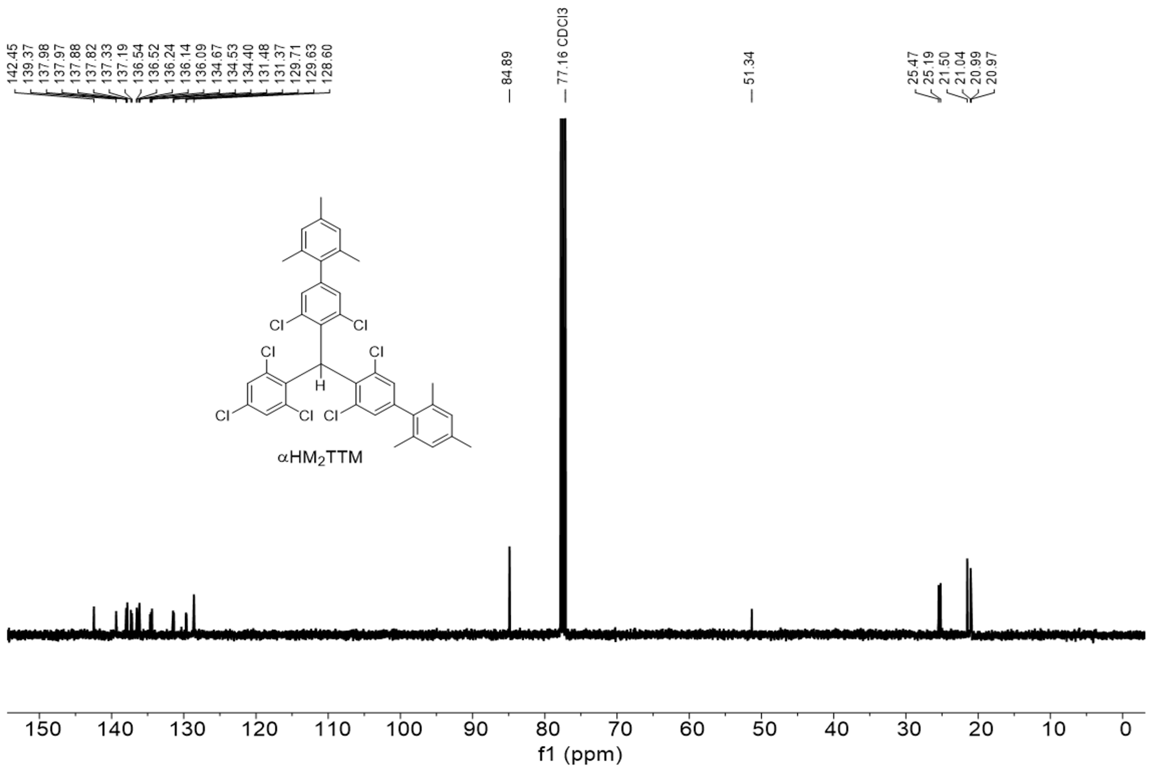
142.45
139.37
137.98
137.97
137.88
137.82
137.33
137.19
136.54
136.52
136.24
136.14
136.09
134.67
134.53
134.40
131.48
131.37
129.71
129.63
128.60
84.89
77.16 CDCl3
51.34
25.47
25.19
21.50
21.04
20.99
20.97
Cl
H
αHM2TTM
150 140 130 120 110 100 90 80 70 60 50 40 30 20 10 0
f1 (ppm)

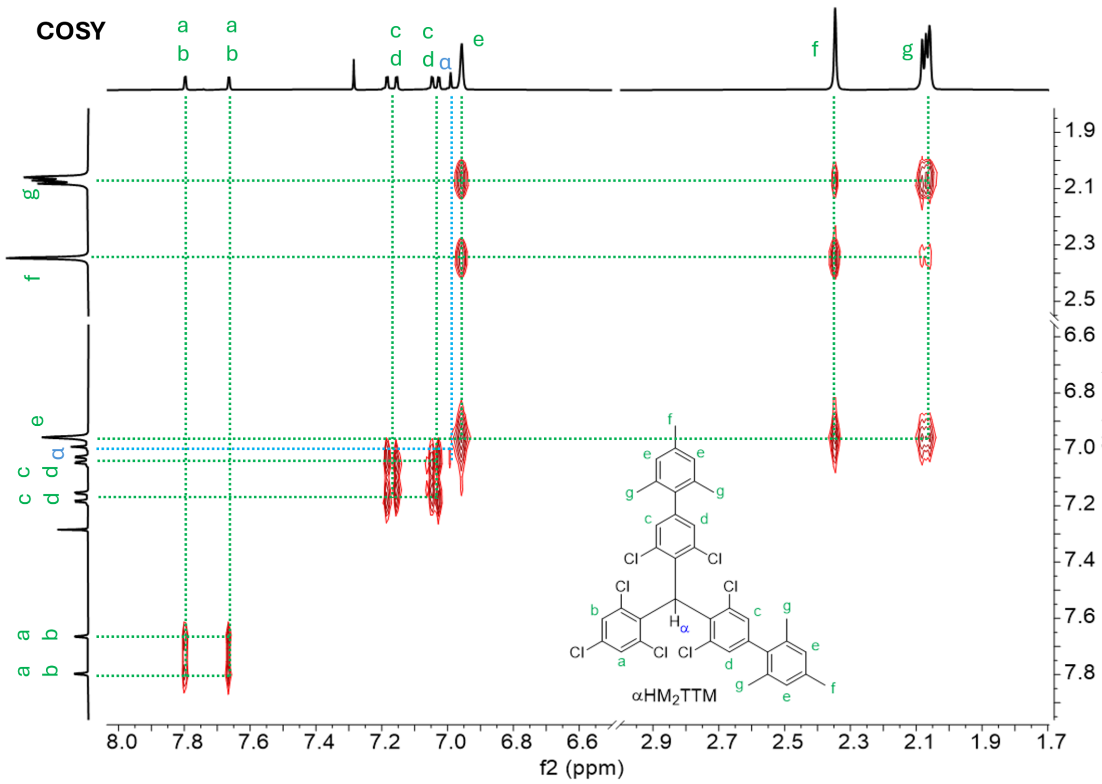
COSY
a
b
c
d
α
e
f
g
Cl
Hα
αHM2TTM
f2 (ppm)
f1 (ppm)
8.0
7.8
7.6
7.4
7.2
7.0
6.8
6.6
2.9
2.7
2.5
2.3
2.1
1.9
1.7

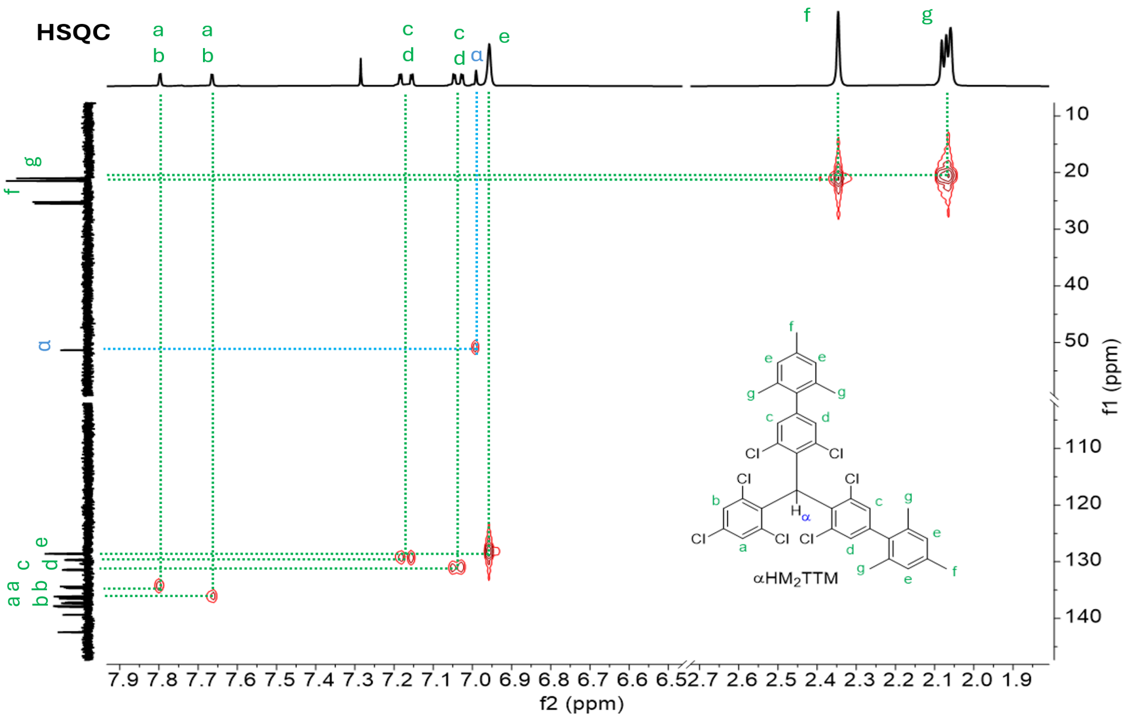
HSQC
a
b
a
b
c
d
c
d
α
e
f
g
f
g
α
e
c
d
a a
b b
αHM2TTM
f2 (ppm)
f1 (ppm)

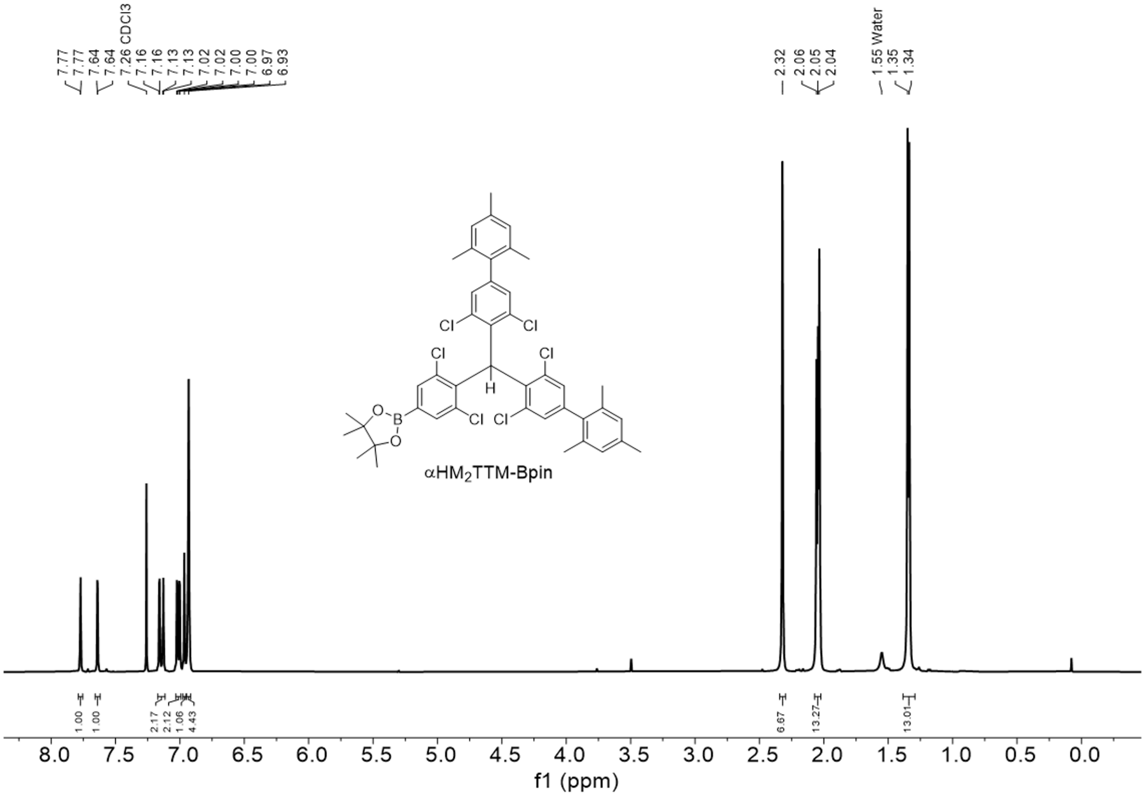
7.77
7.77
7.64
7.64
7.26 CDCl3
7.16
7.16
7.13
7.13
7.02
7.02
7.00
7.00
6.97
6.93
2.32
2.06
2.05
2.04
1.55 Water
1.35
1.34
Cl
H
O
B
αHM2TTM-Bpin
1.00
1.00
2.17
2.12
1.06
4.43
6.67
13.27
13.01
8.0 7.5 7.0 6.5 6.0 5.5 5.0 4.5 4.0 3.5 3.0 2.5 2.0 1.5 1.0 0.5 0.0
f1 (ppm)

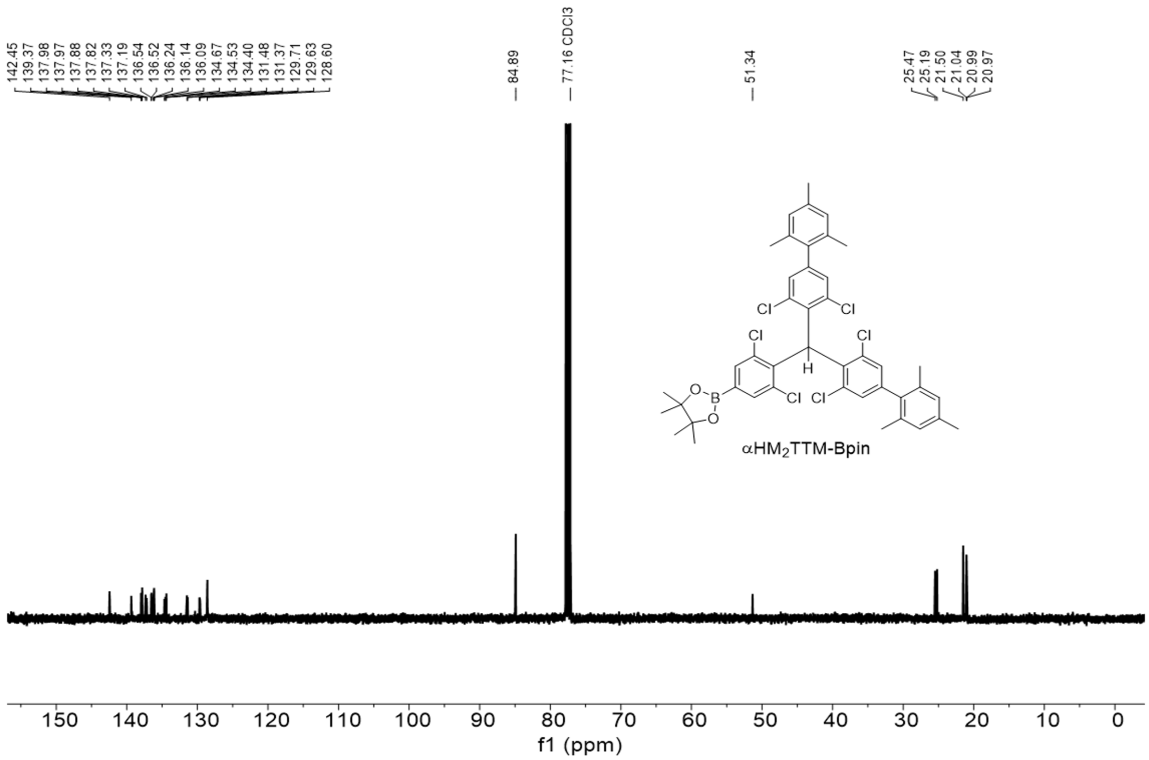

142.45
139.37
137.98
137.97
137.88
137.82
137.33
137.19
136.54
136.52
136.24
136.14
136.09
134.67
134.53
134.40
131.48
131.37
129.71
129.63
128.60
84.89
77.16 CDCl3
51.34
25.47
25.19
21.50
21.04
20.99
20.97
Cl
H
O
B
αHM2TTM-Bpin
150 140 130 120 110 100 90 80 70 60 50 40 30 20 10 0
f1 (ppm)

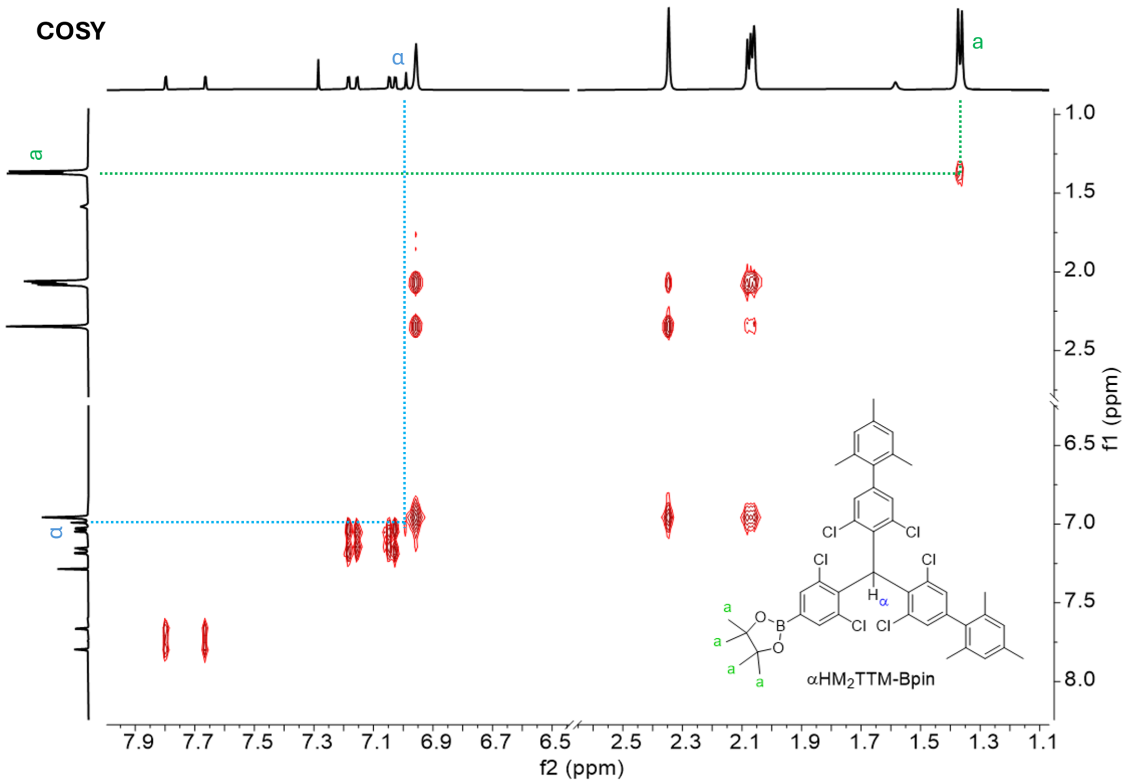

COSY
α
a
f1 (ppm)
1.0
1.5
2.0
2.5
6.5
7.0
7.5
8.0
f2 (ppm)
7.9
7.7
7.5
7.3
7.1
6.9
6.7
6.5
2.5
2.3
2.1
1.9
1.7
1.5
1.3
1.1
Cl
Hα
O
B
αHM₂TTM-Bpin

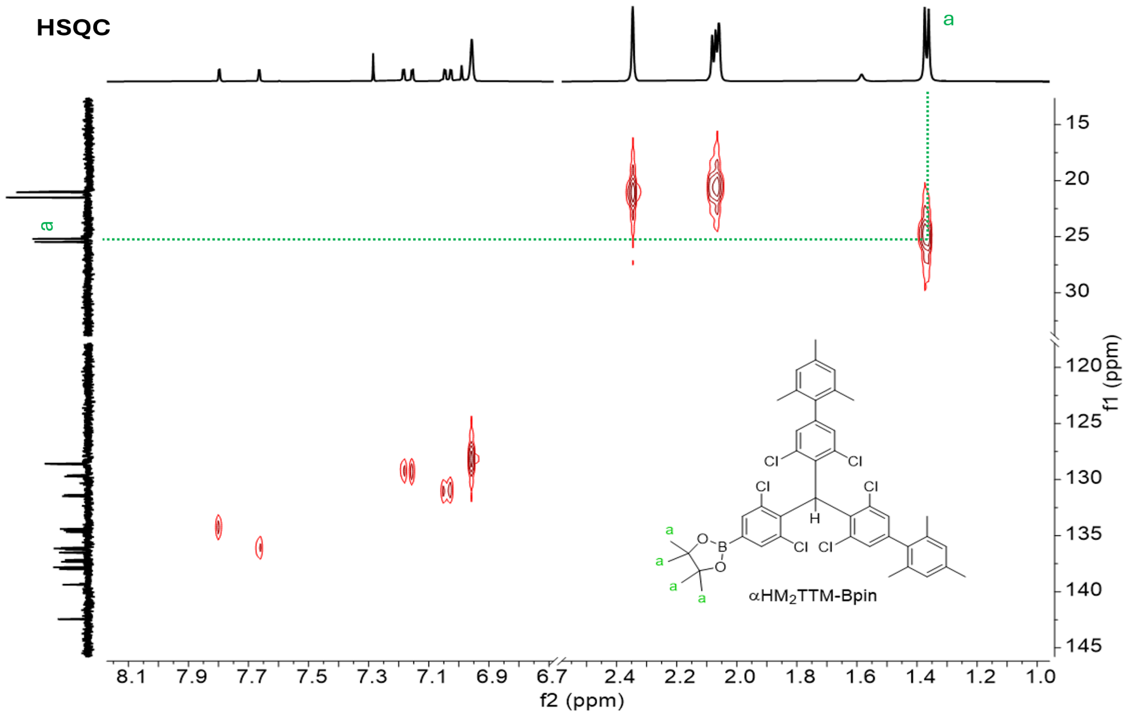

HSQC
a
f1 (ppm)
f2 (ppm)
αHM₂TTM-Bpin

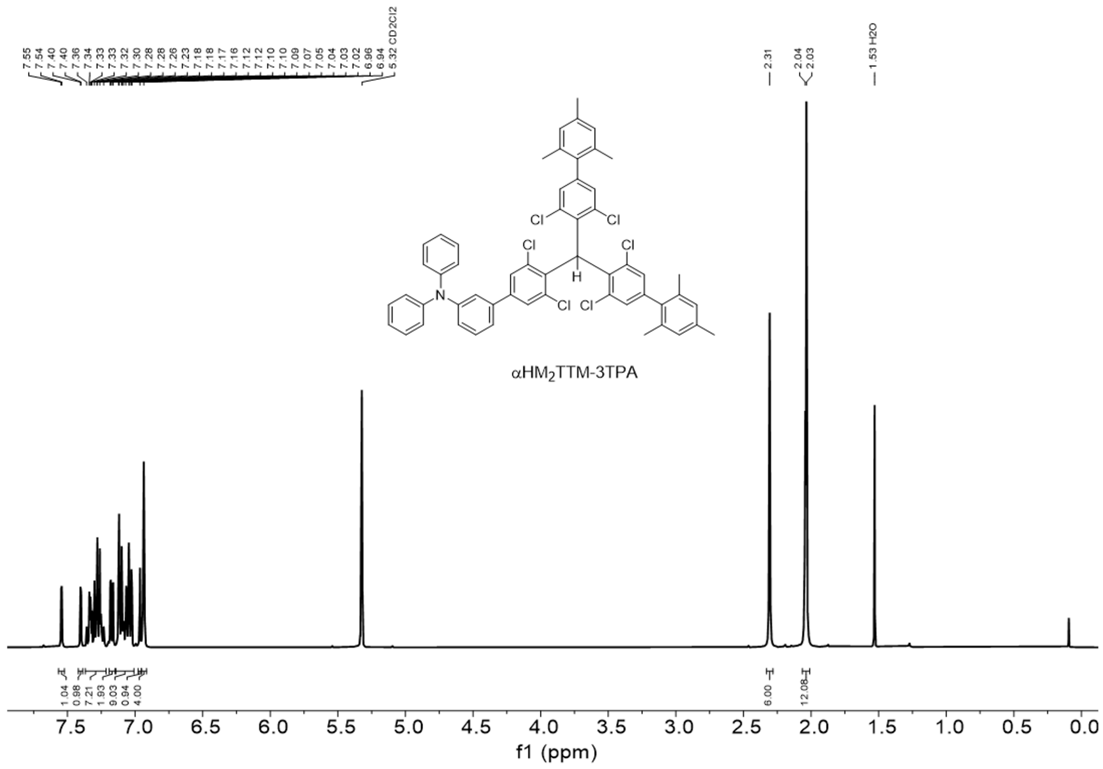
αHM2TTM-3TPA
f1 (ppm)

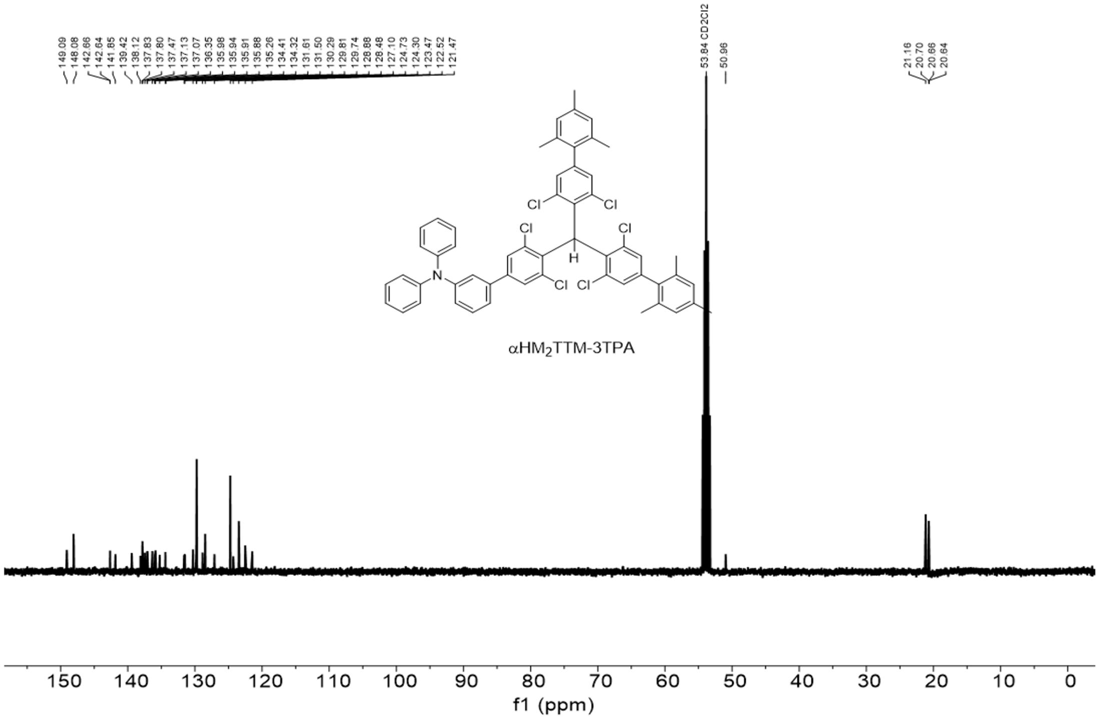

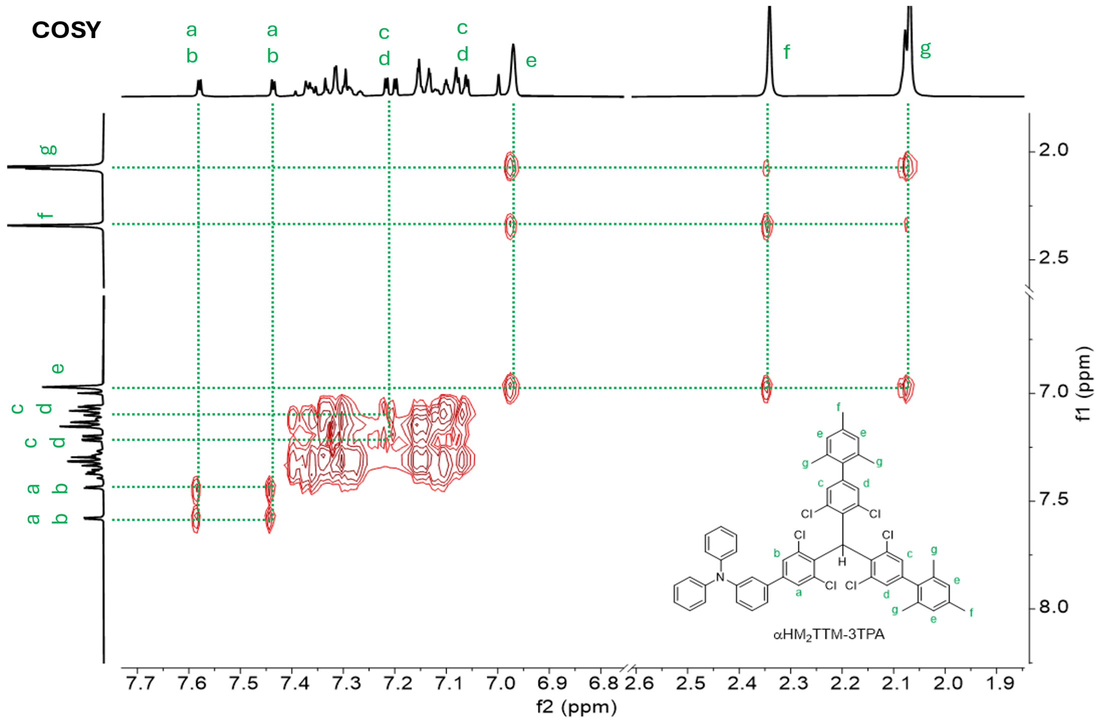

COSY
a
b
c
d
e
f
g
αHM2TTM-3TPA
Cl
H
N
f2 (ppm)
f1 (ppm)

HSQC

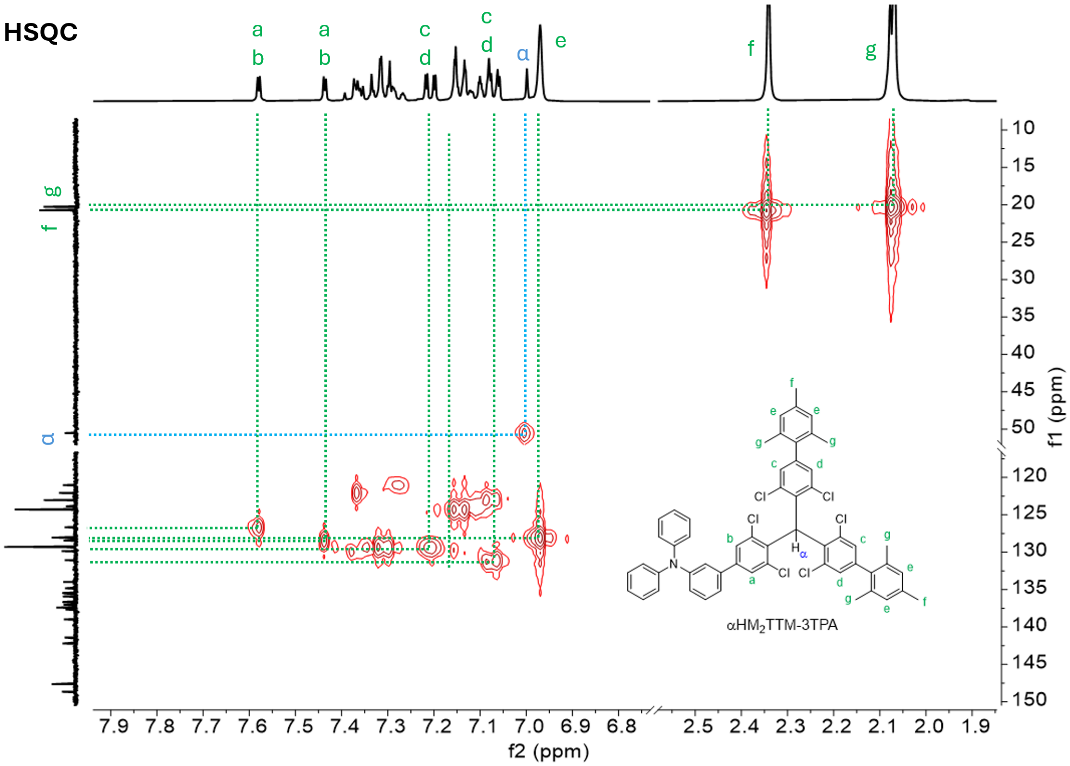

Radical

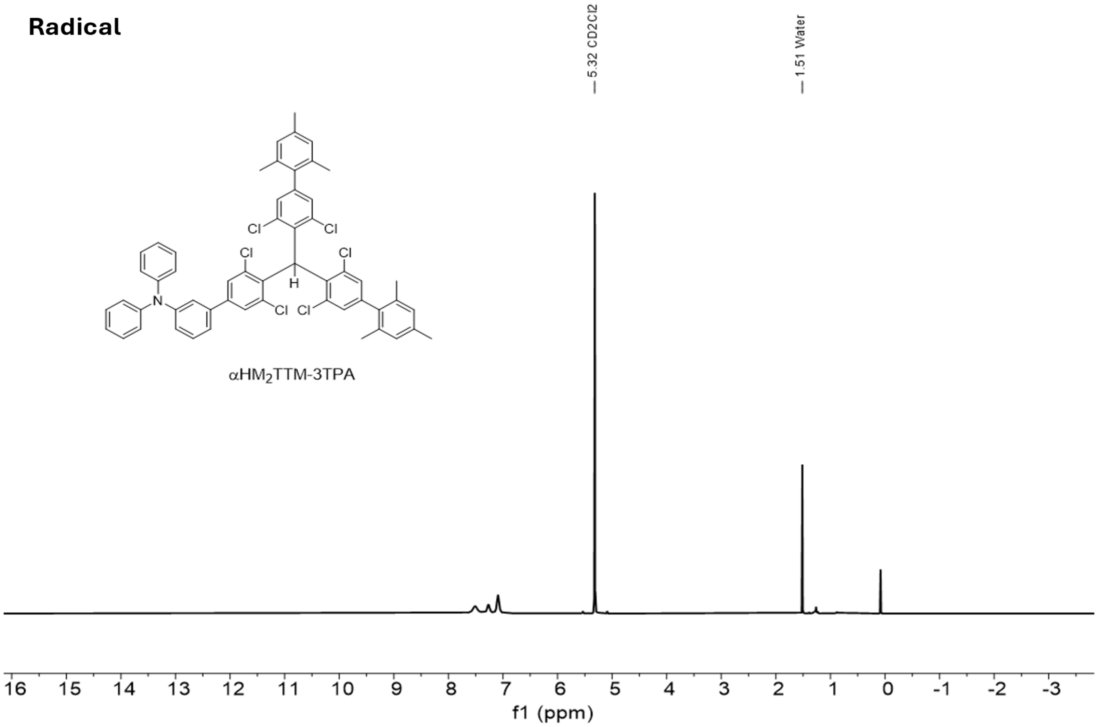

αHM$_2$TTM-3TPA

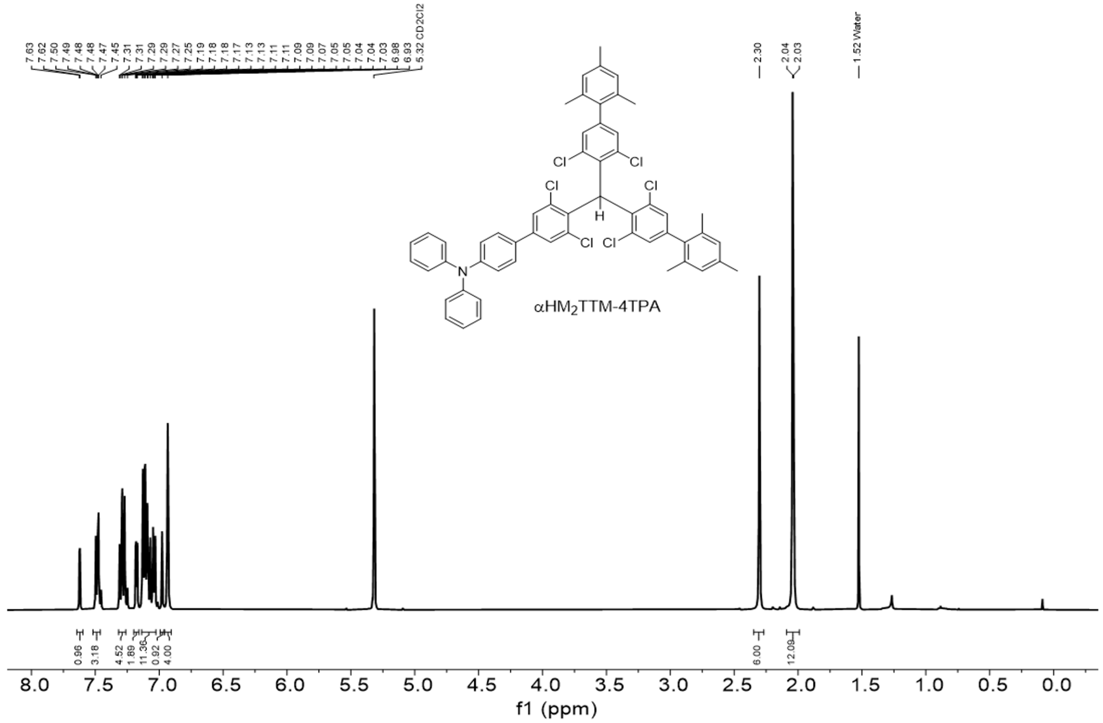
αHM2TTM-4TPA
f1 (ppm)
5.32 CD2Cl2
1.52 Water

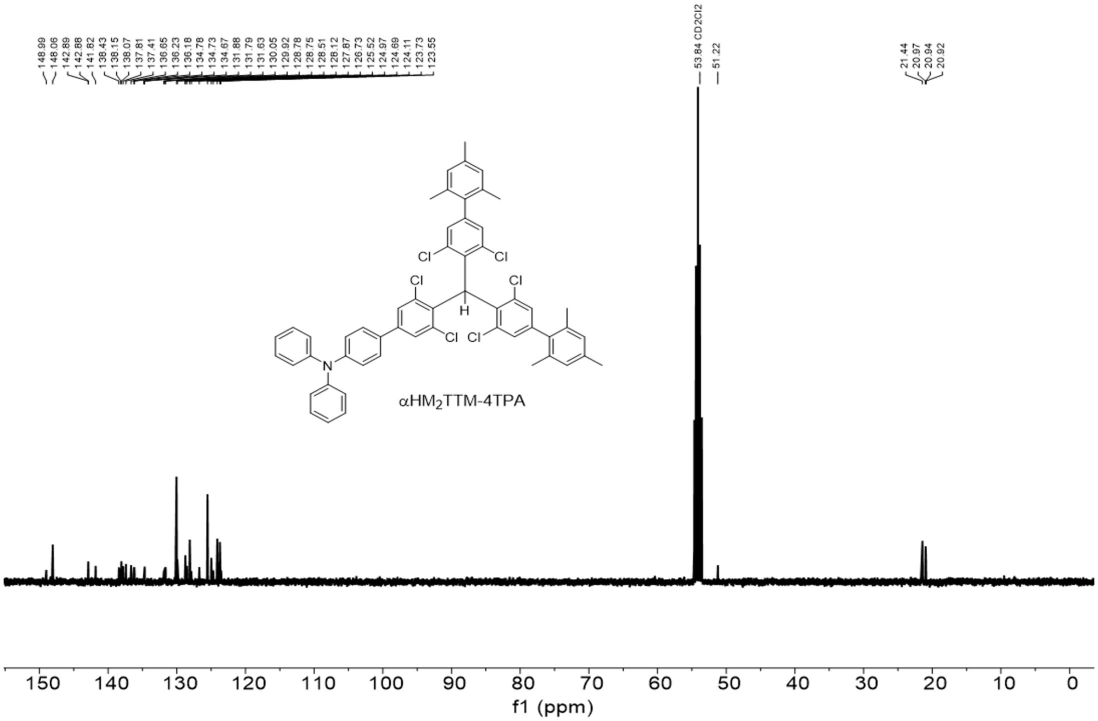
148.99
148.06
142.89
142.88
141.82
138.43
138.15
138.07
137.81
137.41
136.65
136.23
136.18
134.78
134.73
134.67
131.88
131.79
131.63
130.05
129.92
128.78
128.75
128.51
128.12
127.87
126.73
125.52
124.97
124.69
124.11
123.73
123.55
53.84 CD2Cl2
51.22
21.44
20.97
20.94
20.92
Cl
H
N
αHM2TTM-4TPA
150 140 130 120 110 100 90 80 70 60 50 40 30 20 10 0
f1 (ppm)

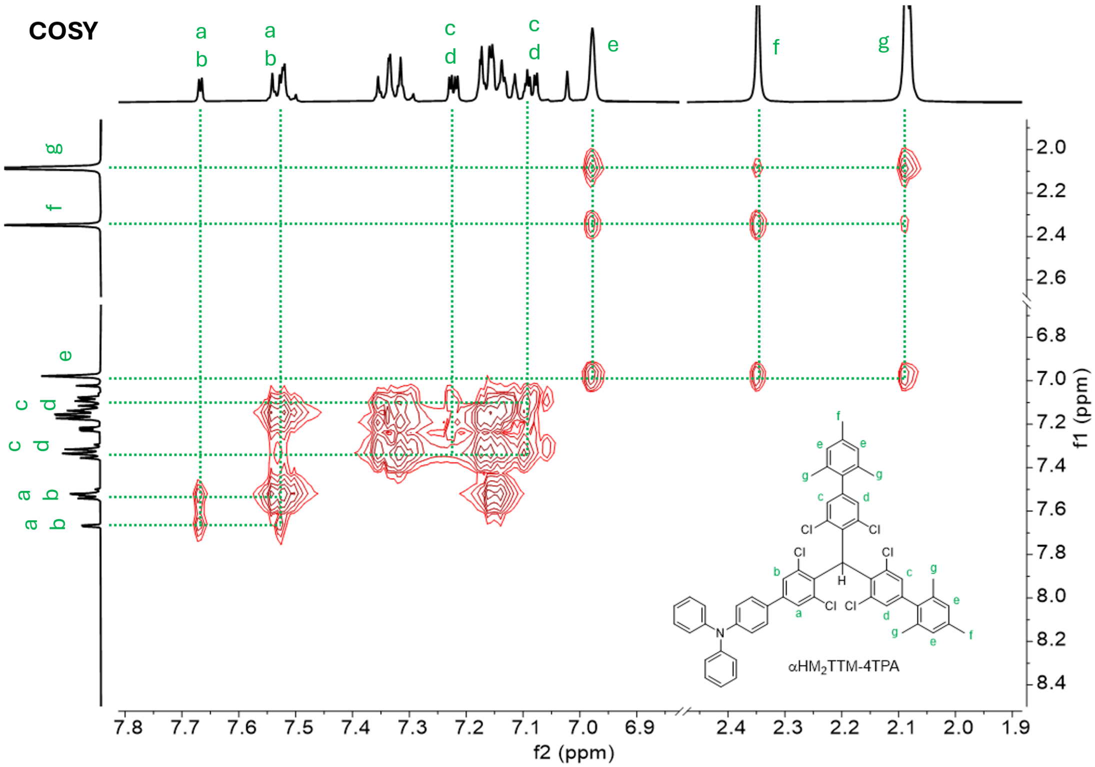
COSY
f2 (ppm)
f1 (ppm)
αHM2TTM-4TPA

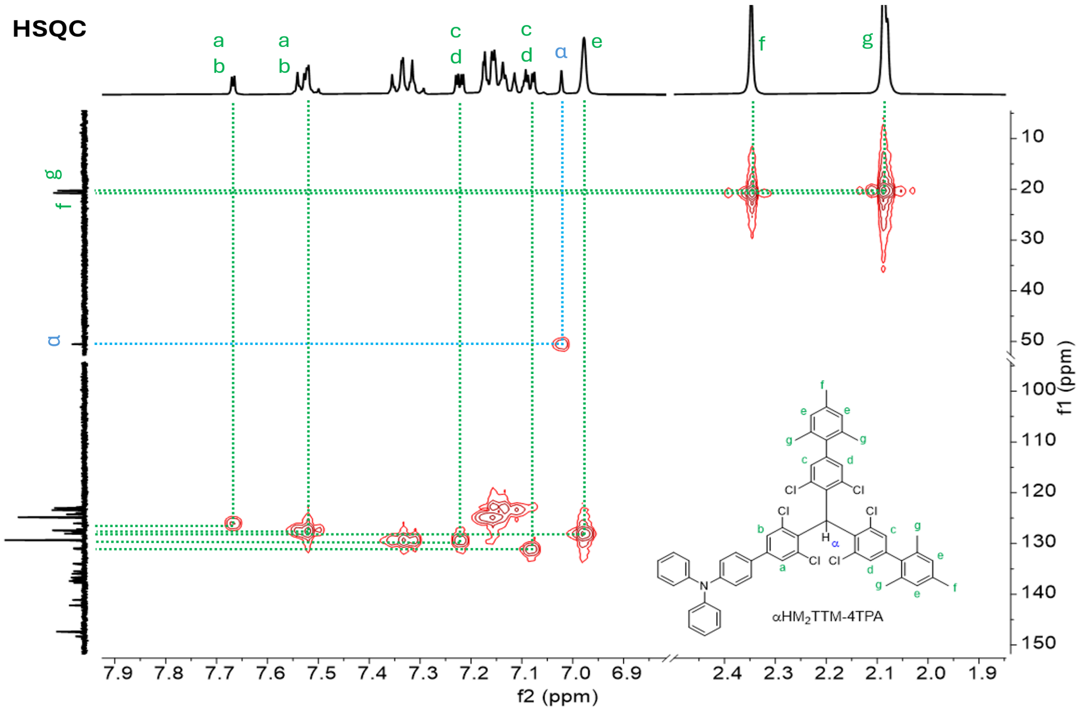
HSQC
a
b
a
b
c
d
c
d
α
e
f
g
f2 (ppm)
f1 (ppm)
αHM2TTM-4TPA

Radical

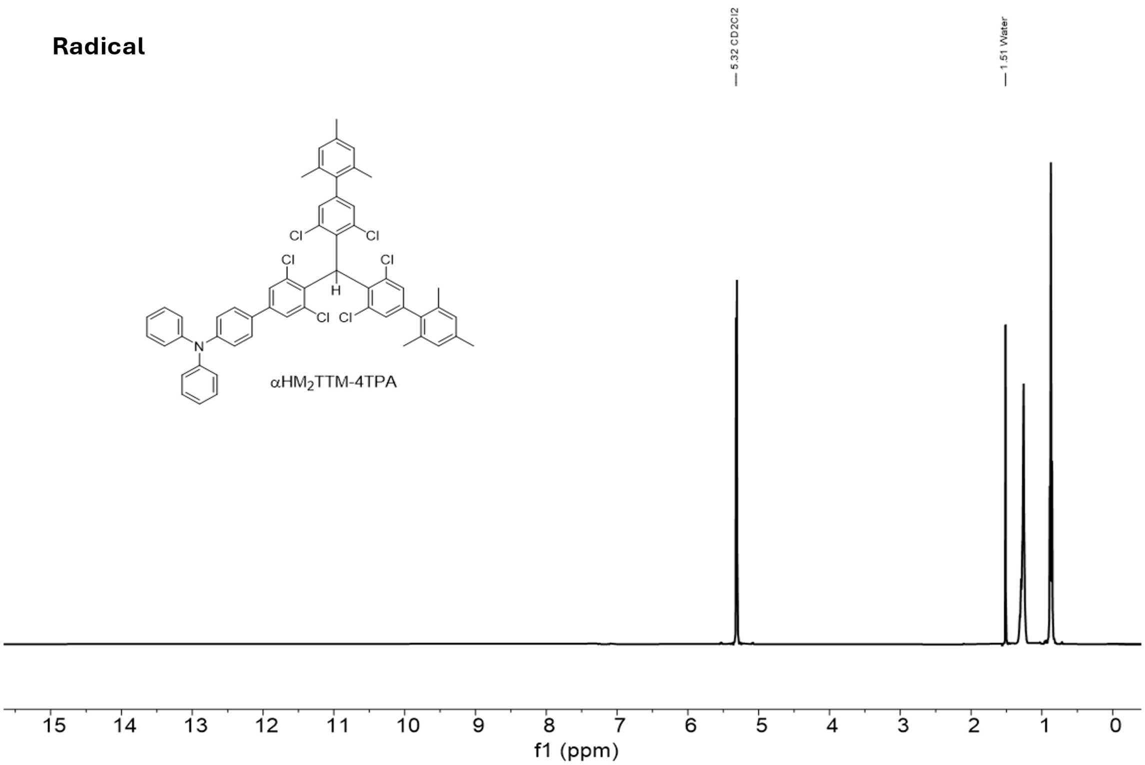
5.32 CD2Cl2
1.51 Water
Cl
Cl
Cl
Cl
Cl
Cl
H
N
αHM2TTM-4TPA
15 14 13 12 11 10 9 8 7 6 5 4 3 2 1 0
f1 (ppm)

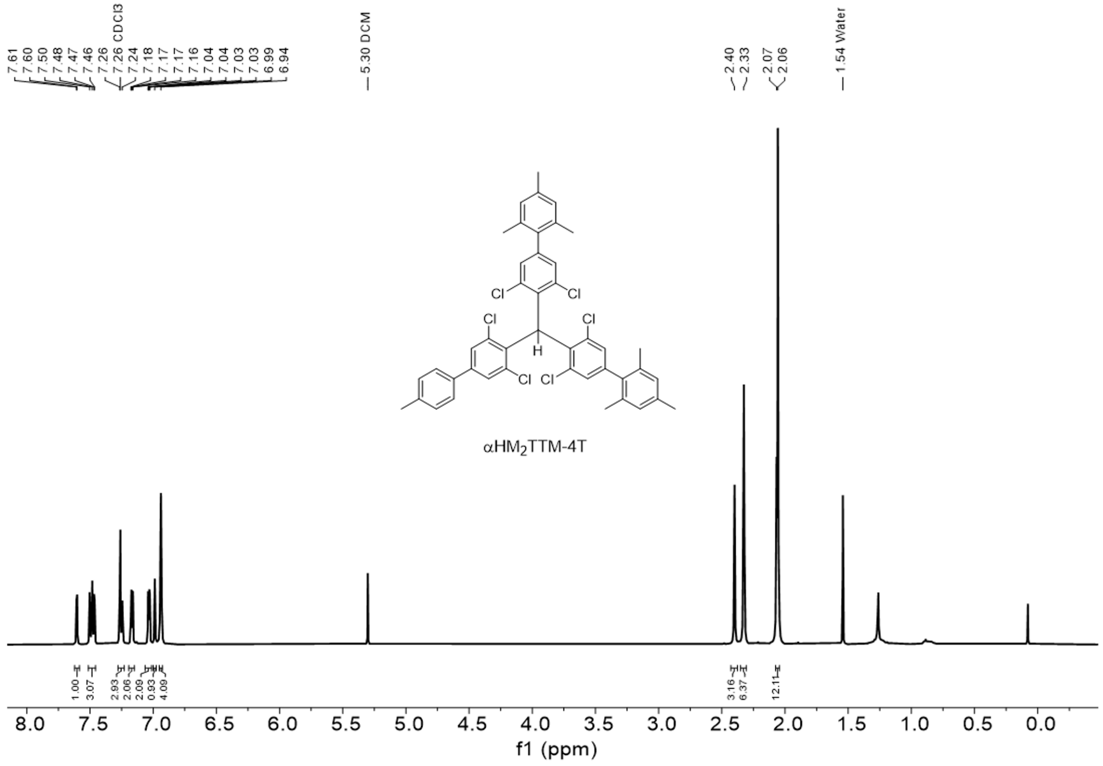
7.61
7.60
7.50
7.48
7.47
7.46
7.26
7.26 CDCl3
7.24
7.18
7.17
7.17
7.16
7.04
7.04
7.03
7.03
6.99
6.94
5.30 DCM
2.40
2.33
2.07
2.06
1.54 Water
Cl
H
αHM2TTM-4T
1.00
3.07
2.93
2.06
2.09
0.93
4.09
3.16
6.37
12.11
8.0 7.5 7.0 6.5 6.0 5.5 5.0 4.5 4.0 3.5 3.0 2.5 2.0 1.5 1.0 0.5 0.0
f1 (ppm)

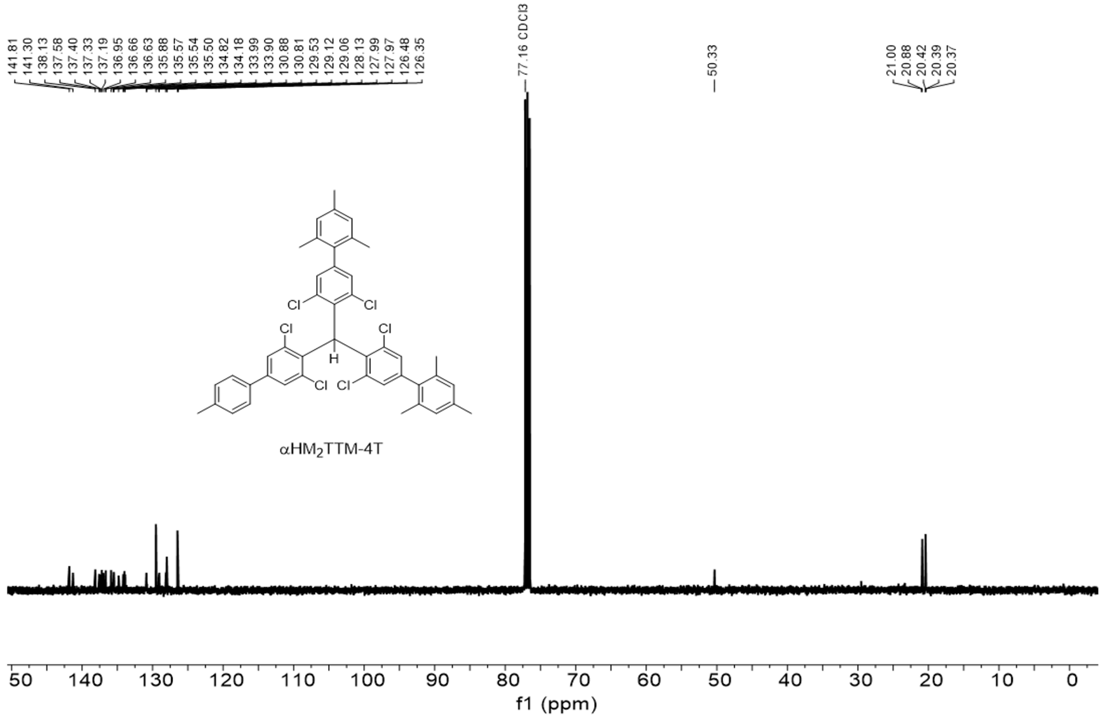
141.81
141.30
138.13
137.58
137.40
137.33
137.19
136.95
136.66
136.63
135.88
135.57
135.54
135.50
134.82
134.18
133.99
133.90
130.88
130.81
129.53
129.12
129.06
128.13
127.99
127.97
126.48
126.35
77.16 CDCl3
50.33
21.00
20.88
20.42
20.39
20.37
Cl
H
αHM2TTM-4T
50 140 130 120 110 100 90 80 70 60 50 40 30 20 10 0
f1 (ppm)

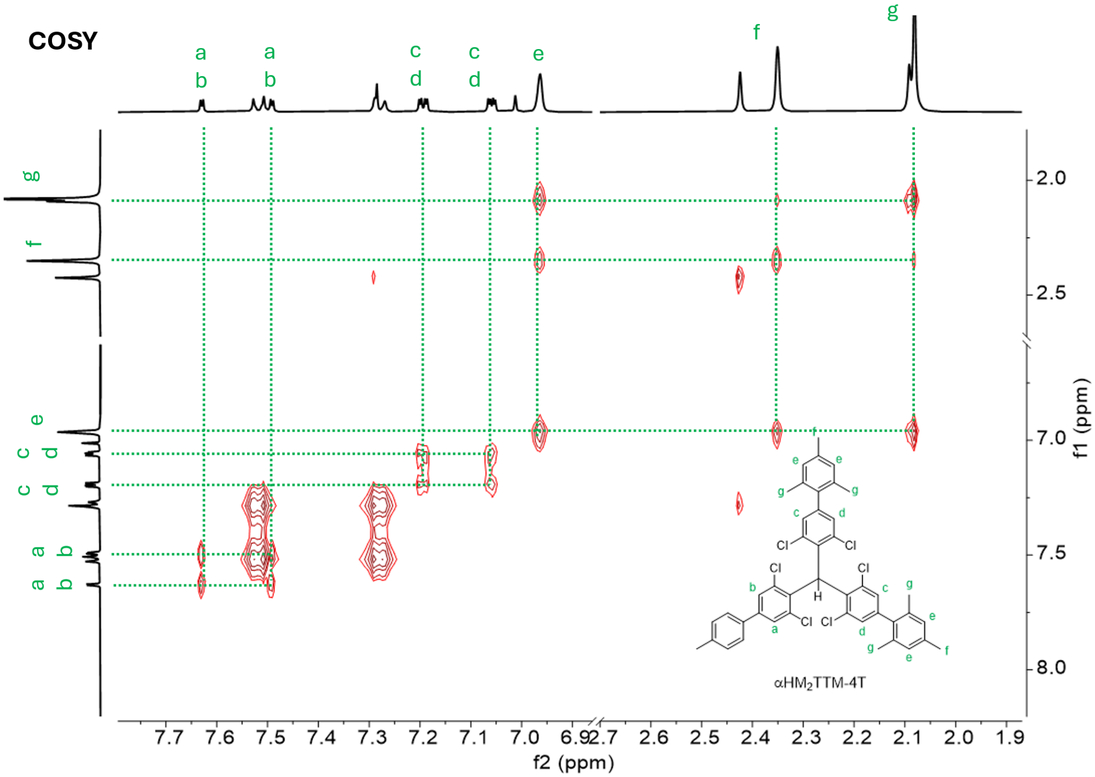
COSY
a
b
c
d
e
f
g
f2 (ppm)
f1 (ppm)
αHM₂TTM-4T
Cl
H

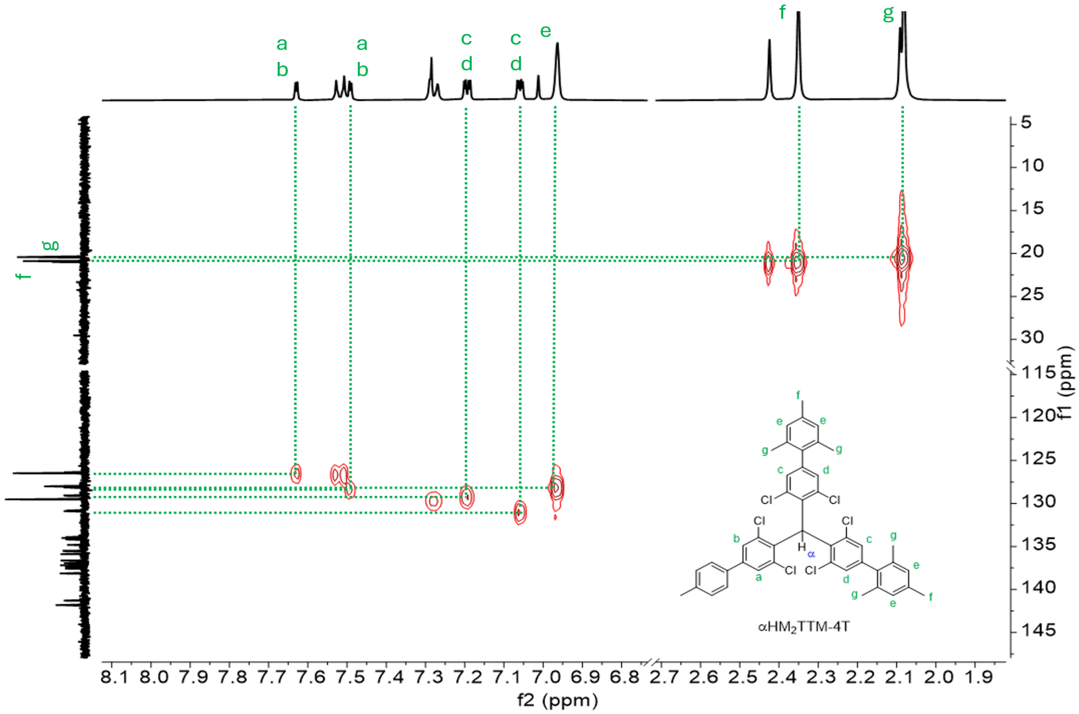
a
b
a
b
c
d
c
d
e
f
g
f
g
5
10
15
20
25
30
115
120
125
130
135
140
145
f1 (ppm)
8.1 8.0 7.9 7.8 7.7 7.6 7.5 7.4 7.3 7.2 7.1 7.0 6.9 6.8 2.7 2.6 2.5 2.4 2.3 2.2 2.1 2.0 1.9
f2 (ppm)
Cl
H
α
αHM2TTM-4T

Radical

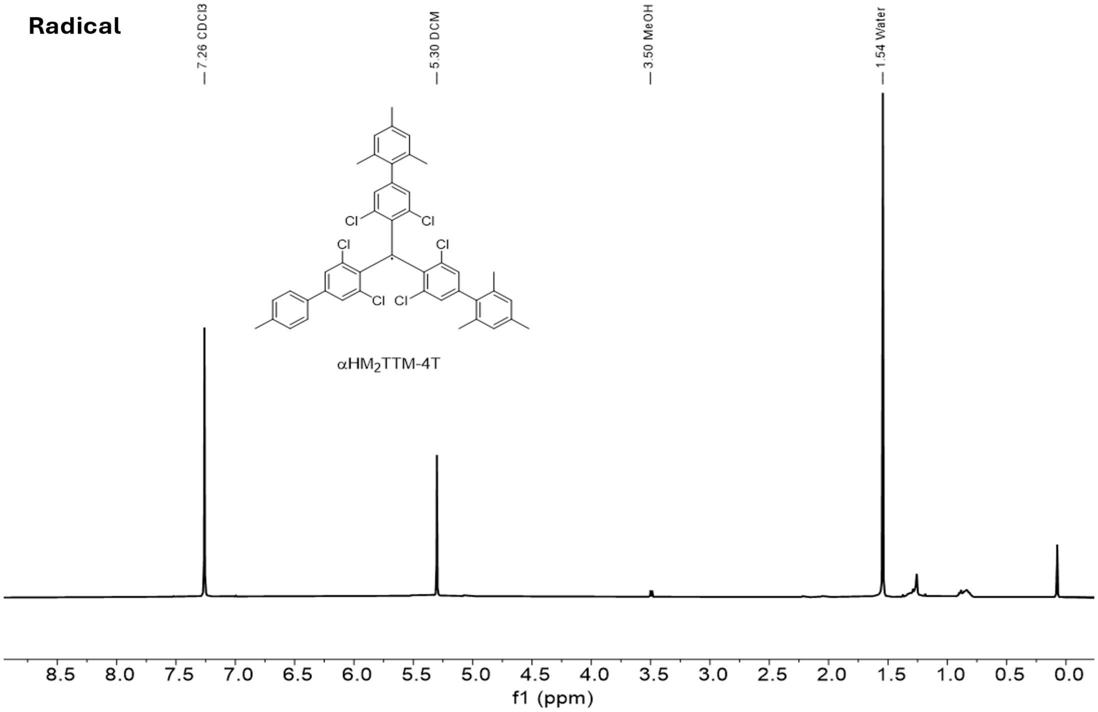
7.26 CDCl3
5.30 DCM
3.50 MeOH
1.54 Water
Cl
Cl
Cl
Cl
Cl
Cl
αHM2TTM-4T
8.5 8.0 7.5 7.0 6.5 6.0 5.5 5.0 4.5 4.0 3.5 3.0 2.5 2.0 1.5 1.0 0.5 0.0
f1 (ppm)

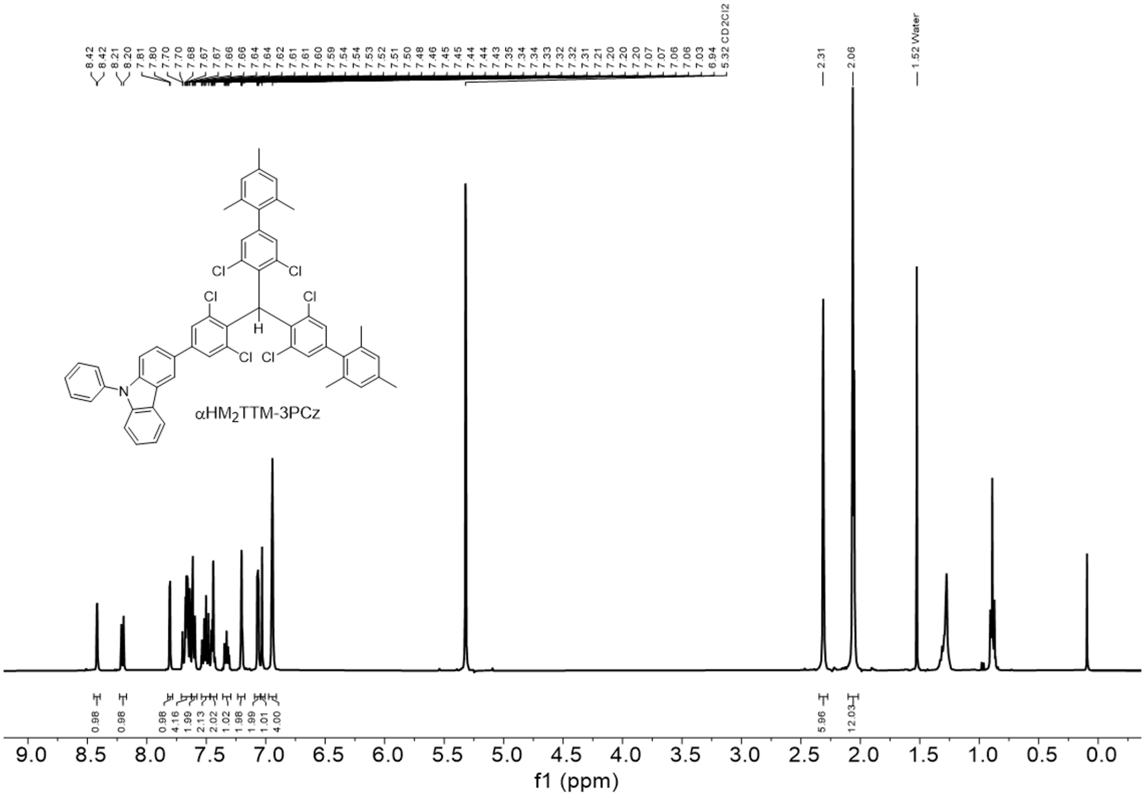
8.42
8.42
8.21
8.20
7.81
7.80
7.70
7.68
7.67
7.67
7.66
7.66
7.64
7.64
7.62
7.61
7.61
7.60
7.59
7.54
7.54
7.53
7.52
7.51
7.50
7.48
7.46
7.45
7.45
7.44
7.44
7.43
7.35
7.34
7.34
7.33
7.32
7.32
7.31
7.21
7.20
7.20
7.20
7.07
7.07
7.06
7.05
7.03
6.94
5.32 CD2Cl2
2.31
2.06
1.52 Water
Cl
H
N
αHM2TTM-3PCz
0.98
0.98
0.98
4.16
1.99
2.13
2.02
1.02
1.98
1.99
1.01
4.00
5.96
12.03
9.0 8.5 8.0 7.5 7.0 6.5 6.0 5.5 5.0 4.5 4.0 3.5 3.0 2.5 2.0 1.5 1.0 0.5 0.0
f1 (ppm)

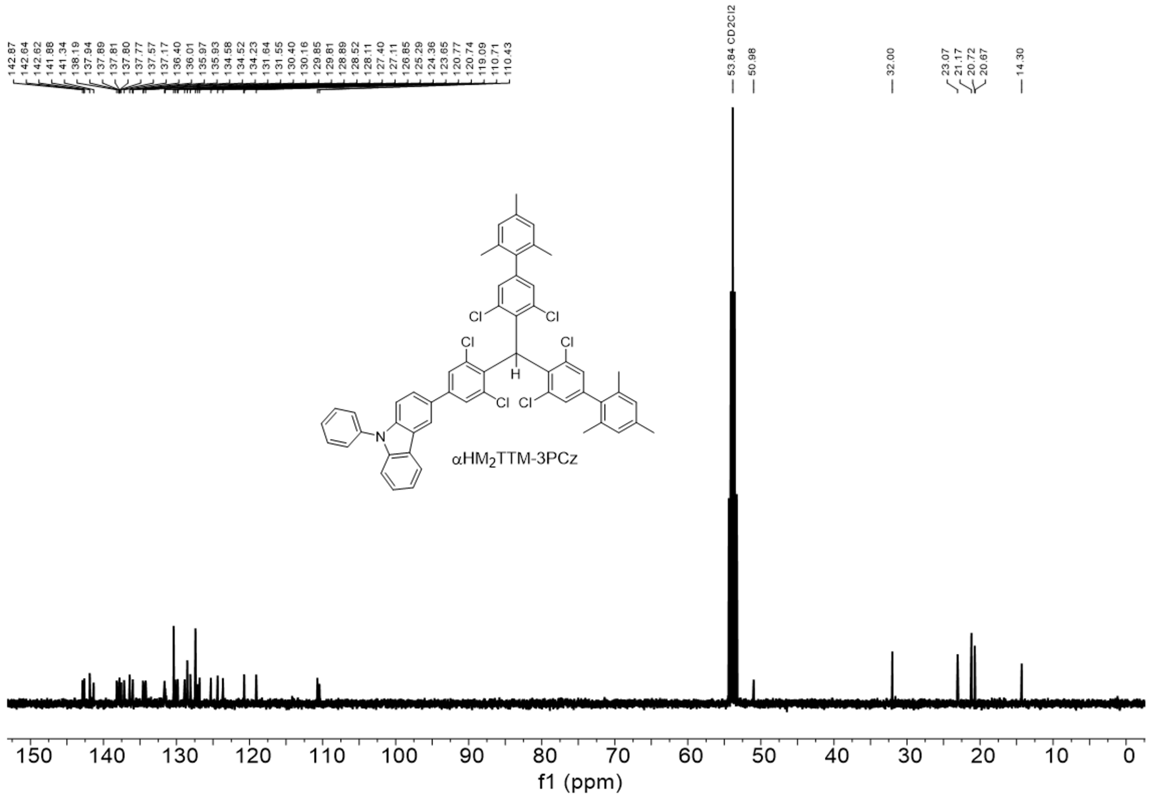

142.87
142.64
142.62
141.88
141.34
138.19
137.94
137.89
137.81
137.80
137.77
137.57
137.17
136.40
136.01
135.97
135.93
134.58
134.52
134.23
131.64
131.55
130.40
130.16
129.85
129.81
128.89
128.52
128.11
127.40
127.11
126.85
125.29
124.36
123.65
120.77
120.74
119.09
110.71
110.43
53.84 CD2Cl2
50.98
32.00
23.07
21.17
20.72
20.67
14.30
Cl
Cl
Cl
Cl
Cl
Cl
H
N
αHM2TTM-3PCz
f1 (ppm)
150 140 130 120 110 100 90 80 70 60 50 40 30 20 10 0

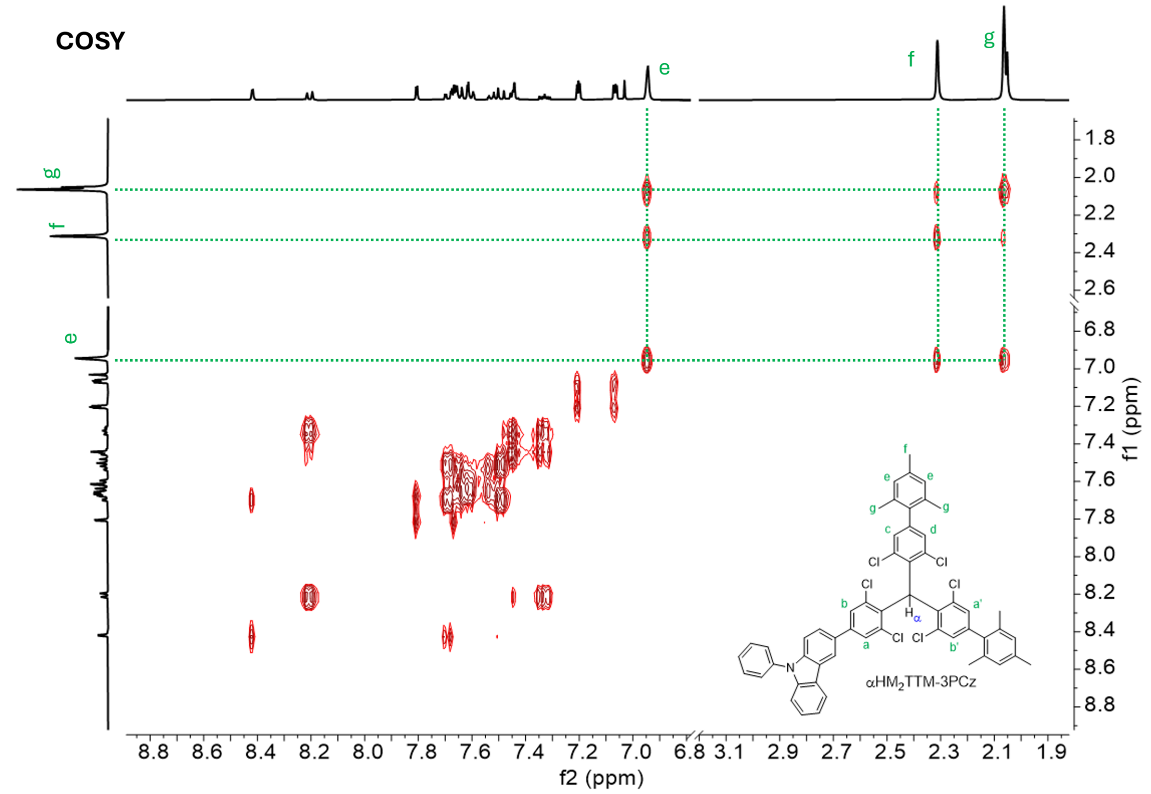

COSY
e
f
g
αHM2TTM-3PCz
f2 (ppm)
f1 (ppm)

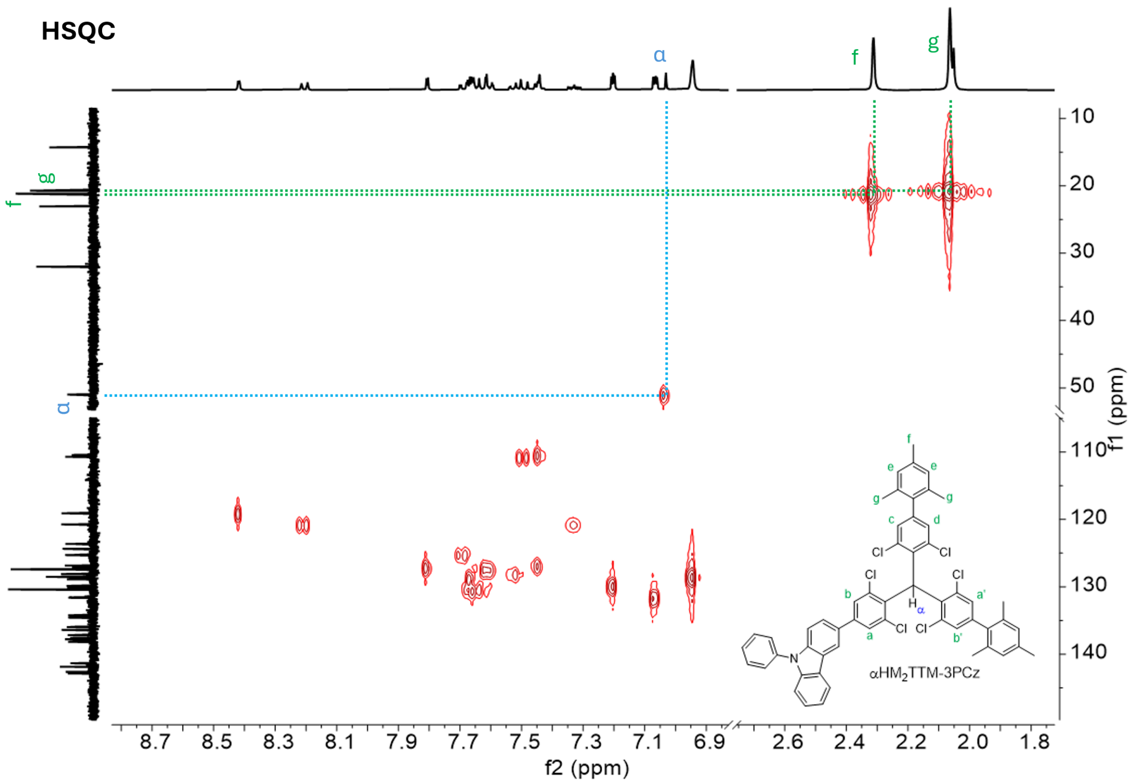
HSQC
α
f
g
f2 (ppm)
f1 (ppm)
αHM2TTM-3PCz

Radical

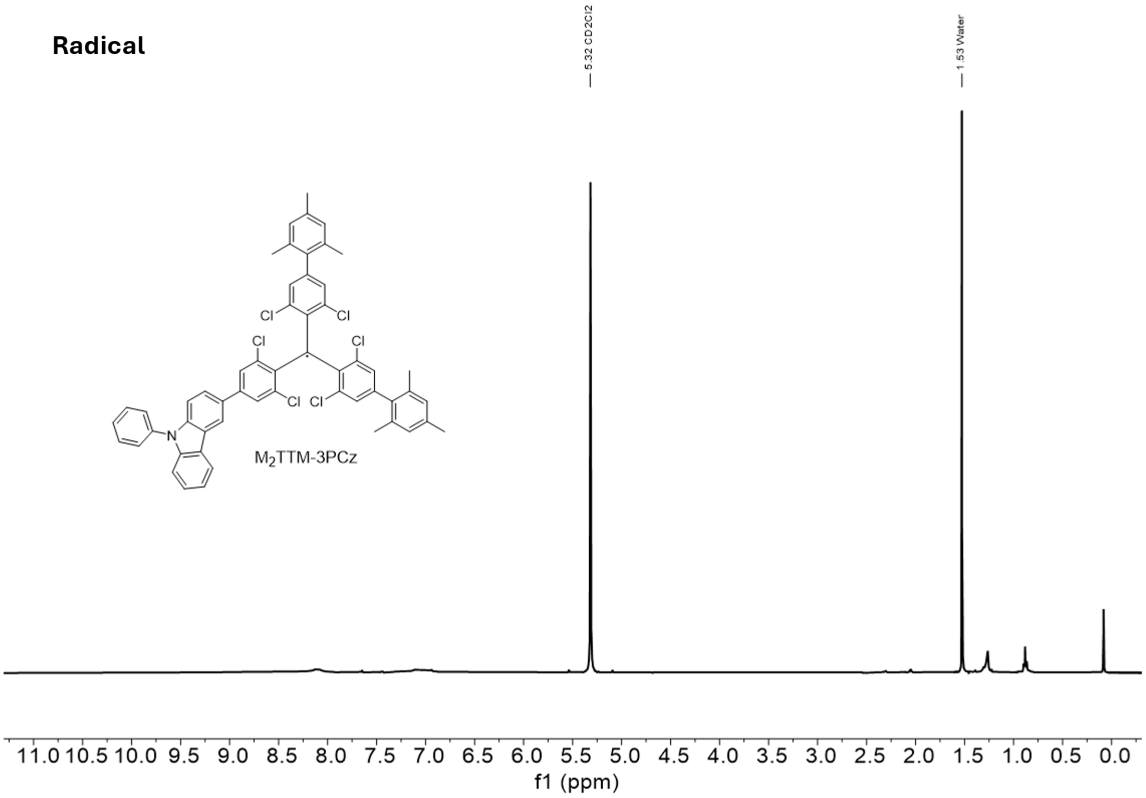

REFERENCES

[1]A. Mizuno, R. Matsuoka, T. Mibu and T. Kusamoto, *Chemical Reviews* **124** (2024), 1034, pMID: 38230673.

[2]C.-W. Ju, Y. Shen, E. J. French, J. Yi, H. Bi, A. Tian and Z. Lin, *The Journal of Physical Chemistry A* **128** (2024), 2457, pMID: 38382058.

[3]J. D. Green and T. J. H. Hele, *The Journal of Chemical Physics* **160** (2024), 164110.

[4]P. Murto, R. Chowdhury, S. Gorgon, E. Guo, W. Zeng, B. Li, Y. Sun, H. Francis,

R. H. Friend and H. Bronstein, *Nature Communications* **14** (2023), 4147.

[5]X. Ai, E. W. Evans, S. Dong, A. J. Gillett, H. Guo, Y. Chen, T. J. H. Hele, R. H. Friend and F. Li, *Nature* **563** (2018), 536.

[6]S. Gorgon, K. Lv, J. Grüne, B. H. Drummond, W. K. Myers, G. Londi, G. Ricci, D. Valverde, C. Tonnelé, P. Murto, A. S. Romanov, D. Casanova, V. Dyakonov, A. Sperlich, D. Beljonne, Y. Olivier, F. Li, R. H. Friend and E. W. Evans, *Nature* **620** (2023), 538.

[7]A. Abdurahman, T. J. Hele, Q. Gu, J. Zhang, Q. Peng, M. Zhang, R. H. Friend, F. Li and E. W. Evans, *Nature materials* **19** (2020), 1224.

[8]X. Ai, Y. Chen, Y. Feng and F. Li, *Angewandte Chemie International Edition* **57** (2018), 2869.

[9]S. R. Ruberu and M. A. Fox, *The Journal of Physical Chemistry* **97** (1993), 143.

[10]F. Li, A. J. Gillett, Q. Gu, J. Ding, Z. Chen, T. J. Hele, W. K. Myers, R. H. Friend and E. W. Evans, *Nature Communications* **13** (2022), 2744.

[11]K. Matsuda, R. Xiaotian, K. Nakamura, M. Furukori, T. Hosokai, K. Anraku, K. Nakao and K. Albrecht, *Chem. Commun.* **58** (2022), 13443.

[12]Y. Gao, A. Obolda, M. Zhang and F. Li, *Dyes and Pigments* **139** (2017), 644.

[13]S. Dong, A. Obolda, Q. Peng, Y. Zhang, S. Marder and F. Li, *Mater. Chem. Front.* **1** (2017), 2132.

[14]H. Guo, Q. Peng, X.-K. Chen, Q. Gu, S. Dong, E. W. Evans, A. J. Gillett, X. Ai, M. Zhang, D. Credgington *et al.*, *Nature materials* **18** (2019), 977.

[15]S. Dong, W. Xu, H. Guo, W. Yan, M. Zhang and F. Li, *Phys. Chem. Chem. Phys.* **20** (2018), 18657.

[16]C. Yan, D. An, W. Chen, N. Zhang, Y. Qiao, J. Fang, X. Lu, G. Zhou and Y. Liu, *CCS Chemistry* **4** (2022), 3190.

[17]L.-r. Fei, J. Wang, F.-q. Bai, S.-p. Wang, B. Hu, C.-p. Kong and H.-x. Zhang, *Phys. Chem. Chem. Phys.* **25** (2023), 25871.

[18]G. M. J. Barca, C. Bertoni, L. Carrington, D. Datta, N. De Silva, J. E. Deustua, D. G. Fedorov, J. R. Gour, A. O. Gunina, E. Guidez, T. Harville, S. Irle, J. Ivanic, K. Kowalski, S. S. Leang, H. Li, W. Li, J. J. Lutz, I. Magoulas, J. Mato, V. Mironov, H. Nakata, B. Q. Pham, P. Piecuch, D. Poole, S. R. Pruitt, A. P. Rendell, L. B. Roskop, K. Ruedenberg, T. Sattasathuchana, M. W. Schmidt, J. Shen, L. Slipchenko, M. Sosonkina, V. Sundriyal, A. Tiwari, J. L. Galvez Vallejo, B. Westheimer, M. Włoch, P. Xu, F. Zahariev and M. S. Gordon, *The Journal of Chemical Physics* **152** (2020), 154102.

[19]J. D. Green, E. G. Fuemmeler and T. J. H. Hele, *The Journal of Chemical Physics* **156** (2022), 180901.

[20]J. Hinze and D. L. Beveridge, *Journal of the American Chemical Society* **93** (1971), 3107.

[21]N. Mataga and K. Nishimoto, *Zeitschrift für Physikalische Chemie* **13** (1957), 140.

[22]D. Zhang, Z. Qu, C. Liu and Y. Jiang, *The Journal of Chemical Physics* **134** (2011), 024114.

[23]F. Van-Catledge, *The Journal of Organic Chemistry* **45** (1980), 4801.

[24]K. P. Huber and G. H. Herzberg, Constants of diatomic molecules, (data prepared by Jean W. Gallagher and Russell D. Johnson, III) in NIST Chemistry WebBook, NIST Standard Reference Database Number 69, Eds. P.J. Linstrom and W.G. Mallard, National Institute of Standards and Technology, Gaithersburg MD, 20899, https://doi.org/10.18434/T4D303, (retrieved November 14, 2024).

[25]P. W. Atkins and R. S. Friedman, *Molecular quantum mechanics*, Oxford university press (2011).

[26]H. C. Longuet-Higgins and J. A. Pople, *Proceedings of the Physical Society. Section A* **68** (1955), 591.

[27]D. Maurice and M. Head-Gordon, *The Journal of Physical Chemistry* **100** (1996), 6131.

[28]T. J. H. Hele, *Physical Chemistry of Semiconductor Materials and Interfaces XX*, International Society for Optics and Photonics, SPIE (2021), volume 11799, 117991A.

[29]K. Nishimoto and L. S. Forster, *Theoretica chimica acta* **3** (1965), 407.

[30]T. G. Schmalz, L. Serrano-Andrés, V. Sauri, M. Merchán and J. M. Oliva, *The Journal of chemical physics* **135** (2011).

[31]J. A. Pople, D. P. Santry and G. A. Segal, *The Journal of Chemical Physics* **43** (1965), S129.

[32]R. Pariser and R. G. Parr, *The Journal of Chemical Physics* **21** (1953), 767.

[33]S. D. Bruijn, *Theoretical Chemistry Account* **18** (1970), 34.
